# History of the terrestrial isopod genus *Ligidium* in Japan based on phylogeographic analysis

**DOI:** 10.1186/s12862-023-02144-8

**Published:** 2023-08-07

**Authors:** Wakana Harigai, Aya Saito, Chika Zemmoto, Shigenori Karasawa, Touta Yokoi, Atsushi J. Nagano, Hitoshi Suzuki, Masanobu Yamamoto

**Affiliations:** 1 Graduate School of Environmental Science, Hokkaido University, Kita-Ku, Sapporo, 060-0810 Japan; 2 Present address: Department of Child Development and Molecular Brain Science, United Graduate School of Child Development, Osaka University, Suita, 565-0871 Japan; 3 Department of Earth and Planetary Sciences, Faculty of Science, Hokkaido University, Kita-Ku, Sapporo, 060-0810 Japan; 4 Faculty of Agriculture, Tottori University, 4-101, Koyama-Cho Minami, Tottori, 680-8553 Japan; 5 Faculty of Agriculture, Ryukoku University, Otsu, Shiga 520-2194 Japan; 6 Institute for Advanced Biosciences, Keio University, Tsuruoka, Yamagata 997-0017 Japan; 7 Faculty of Environmental Earth Science, Hokkaido University, Kita-Ku, Sapporo, 060-0810 Japan

**Keywords:** Genome-wide analysis, Geology, *Ligidium*, Phylogeography, Quaternary glacial cycle, RAD-seq

## Abstract

**Background:**

Phylogeographical approaches explain the genetic diversity of local organisms in the context of their geological and geographic environments. Thus, genetic diversity can be a proxy for geological history. Here we propose a genus of woodland isopod, *Ligidium*, as a marker of geological history in relation to orogeny and the Quaternary glacial cycle.

**Results:**

Mitochondrial analysis of 721 individuals from 97 sites across Japan revealed phylogenetic divergence between the northeastern and southwestern Japan arcs. It also showed repeated population expansions in northeastern Japan in response to Quaternary glacial and interglacial cycles. Genome-wide analysis of 83 selected individuals revealed multiple genetic nuclear clusters. The genomic groupings were consistent with the local geographic distribution, indicating that the *Ligidium* phylogeny reflects its regional history.

**Conclusion:**

*Ligidium* DNA sequence analysis can provide insight into the geological, geographical, and paleoenvironmental history of the studied region.

**Supplementary Information:**

The online version contains supplementary material available at 10.1186/s12862-023-02144-8.

## Background

The geographic distribution of genetic diversity results from biological history and reflects fragmentation, bottlenecks, sudden population expansions, and migration routes. Studies of phylogeography explain such genetic diversity in the context of past events [1]. An early phylogeographic study identified the refugia and post-warming dispersal routes of various animals, from grasshoppers to bears, in the Quaternary ice ages [2]. The formation of islands following plate movements explains the dramatically different faunas bordering Wallace’s Line in the Indo-Australian Archipelago [3]. South American species divergence was affected by Pleistocene climate change and orogenic events in the Miocene and Pliocene [4]. These results suggest that phylogeographic analysis can provide insight into past geological and environmental events (e.g., [5, 6]). However, the usefulness of the approach has not been evaluated.

**Fig. 1:**
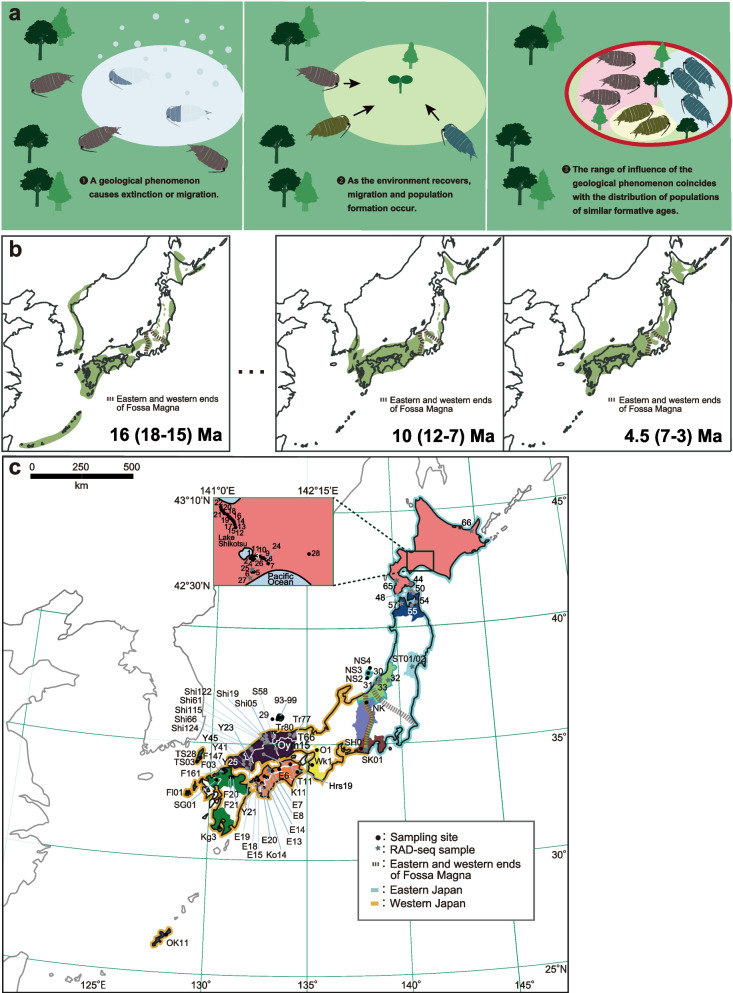
Conceptual diagram of this study and paleogeographic and sampling maps. **a **Conceptual diagram of the method used to analyze *Ligidium* as a paleogeological marker. **b** Paleogeographic map of the Japanese Archipelago. Colored areas are land. Map constructed based on a geological survey of Japan [7]. **c** Map of *Ligidium* sampling sites. Stars, locations where individuals used for restriction site-associated DNA sequencing (RAD-seq) were collected. Illustrations were created in Adobe Illustrator

Conventional paleoenvironmental reconstruction using geological records can generate continuous time-series records at specific sites but is at a disadvantage when reconstructing the spatial distribution of the paleoenvironment. A phylogeographical approach would compensate for this weakness. Such an approach can increase the spatial resolution of the reconstructed paleoenvironment by increasing the spatial sampling density of the target species (Fig. 1a). We selected terrestrial isopods of the genus *Ligidium* as a case study for a phylogeographical analysis with the aim of assessing regional environmental changes in Japan’s paleohistory. *Ligidium* fulfills requirements for a marker organism, including high sensitivity to environmental disturbances (such as climate change or volcanic eruptions), low mobility, marked regional genetic differentiation, and ease of collecting populations.

The following six species have been reported in Japan: *Ligidium japonicum* Verhoeff, 1918; *L. koreanum* Flasarova, 1972; *L. paulum* Nunomura, 1976; *L. ryukyuense* Nunomura, 1983; *L. kiyosumiense* Nunomura, 1983; and *L. iyoense* Nunomura, 1983 [8]. All species except *L. koreanum* are endemic to Japan [8]. The selected genus *Ligidium* exhibits a strong forest preference [9, 10], dislikes dryness, and is not highly mobile [11]. Furthermore, it is easy to collect a sufficient number of samples. Because a large area in the Japanese Archipelago is forested, this genus has a wide distribution. Studies of *Ligidium* have been conducted in Greece, China, and Japan [12–15]. For example, in the Aegean Islands of Greece, *Ligidium* can be sorted into genetic clusters corresponding to their paleogeographic histories, suggesting that the islands’ isolation was a major determinant of their genetic diversity [12]. In China, the uplift of the Qinghai–Tibet Plateau likely affected *Ligidium* species divergence [13]. Our preliminary analysis showed that the Hokkaido population in northern Japan experienced a bottleneck caused by the Last Glacial Maximum (LGM) [14], suggesting that this genus is sensitive to glacial cycles. Furthermore, the phylogeography of independent lineages of *L. japonicum* reflects its dispersal history from the mountains to the plains and peninsulas in the Kanto Plain of Japan [15].

We analyzed the cytochrome oxidase I (COI) region of mitochondrial DNA (mtDNA) and performed genome-wide analysis via restriction site-associated DNA sequencing (RAD-seq) to examine the regional distribution of the genetic diversity of *Ligidium* throughout Japan (Fig. 1c, Table 1). The Honshu arc of the Japanese Archipelago was separated near the Fossa Magna 22–7 million years ago (Ma) (Fig. 1b). The southwestern Japan arc remained relatively stable and exposed, whereas the northeastern Japan arc was submerged in the sea with scattered islands until 3 Ma (Fig. 1b) [7, 16]. Northeastern Japan experienced repeated changes between glacial coniferous and interglacial broadleaf vegetation in response to glacial–interglacial cycles after 2.5 Ma [17]. Based on knowledge of the biology of *Ligidium*, we formulated the following two hypotheses. First, due to its low mobility, *Ligidium* may exhibit east–west divergence reflecting the formation history of the Japanese Archipelago, as seen in insects with low dispersal ability [18].

**Table 1:** Sampling information for *Ligidium* in Japan

Site ID	Locality	N	Latitude	Longitude	Hight	Collection Date
1	Central Hokkaido	4	42°44′42.48"N	141°24′45.02"E	336	2017/7/28
2	Central Hokkaido	3	42°44′10.03"N	141°24′40.14"E	360	2017/7/28
3	Central Hokkaido	3	42°44′39.98"N	141°24′50.37"E	393	2017/7/28
4	Central Hokkaido	1	42°42′51.68"N	141°24′12.72"E	467	2017/7/28
5	Central Hokkaido	2	42°37′30.79"N	141°27′21.81"E	92	2017/7/28
6	Central Hokkaido	3	42°37′06.36"N	141°24′56.01"E	66	2017/7/28
7	Central Hokkaido	3	42°41′19.77"N	141°32′50.01"E	93	2017/7/28
8	Central Hokkaido	3	42°42′04.63"N	141°31′21.25"E	114	2017/7/28
9	Central Hokkaido	3	42°43′45.18"N	141°28′04.87"E	197	2017/7/28
10	Central Hokkaido	2	42°44′50.33"N	141°26′59.00"E	236	2017/7/28
11	Central Hokkaido	4	42°45′20.08"N	141°26′22.84"E	242	2017/7/28
12	Central Hokkaido	2	42°58′19.13"N	141°08′54.48"E	318	2017/8/9
13	Central Hokkaido	3	43°00′10.44"N	141°08′59.91"E	402	2017/8/9
14	Central Hokkaido	3	43°01′21.95"N	141°08′01.87"E	399	2017/8/9
15	Central Hokkaido	3	43°01′58.99"N	141°07′41.63"E	409	2017/8/9
16	Central Hokkaido	3	43°03′07.95"N	141°06′51.08"E	456	2017/8/9
17	Central Hokkaido	3	43°04′11.69"N	141°06′35.54"E	507	2017/8/9
18	Central Hokkaido	3	43°05′07.39"N	141°04′57.35"E	660	2017/8/9
19	Central Hokkaido	3	43°05′30.80"N	141°03′54.01"E	654	2017/8/9
20	Central Hokkaido	3	43°05′53.36"N	141°03′05.81"E	494	2017/8/9
21	Central Hokkaido	3	43°06′27.49"N	141°02′12.00"E	373	2017/8/9
22	Central Hokkaido	5	43°07′11.01"N	141°01′58.97"E	241	2017/8/9
24	Central Hokkaido	16	42°43′15.75"N	141°29′04.62"E	173	2018/5/8
25	Central Hokkaido	16	42°37′50.59"N	141°24′05.92"E	144	2018/5/8
26	Central Hokkaido	16	42°43′01.34"N	141°24′28.63"E	437	2018/9/26
27	Central Hokkaido	20	42°34′02.52"N	141°22′02.02"E	66	2018/9/26
28	Central Hokkaido	19	42°44′44.80"N	141°58′02.29"E	61	2018/9/26
30	Niigata coastline	14	37°54′25.85"N	139°00′5.72"E	19	2019/5/2
31	Mt. Kakuda	22	37°47′27.635"N	138°50′42.971"E	111	2019/5/3
32	Niigata inland	18	37°45′54.143"N	139°25′28.127"E	59	2019/5/4
33	Echigo plain	40	37°50′4.74"N	138°59′58.87"E	4	2019/5/5
44	South Hokkaido	20	41°49′27.95"N	141°07′43.54"E	77	2019/6/30
48	Aomori	13	41°31′39.88"N	140°56′13.98"E	12	2019/7/1
50	Aomori	13	41°26′53.80"N	141°06′52.66"E	76	2019/7/1
54	Aomori	13	41°09′22.21"N	141°17′29.87"E	66	2019/7/2
55	Aomori	19	40°55′38.62"N	140°59′51.41"E	23	2019/7/2
57	Aomori	14	41°02′19.83"N	140°25′41.67"E	9	2019/7/3
65	South Hokkaido	20	42°08′44.66"N	140°02′06.28"E	42	2019/7/4
66	East Hokkaido	22	44°03′32.723"N	144°59′46.751"E	86	2019/7/9
ST01	Sendai	8	38°14′21.012"N	140°48′30.995"E	122	2020/3/20
ST02	Sendai	8	38°14′03.983"N	140°48′15.011"E	192	2020/3/20
NS2	Sado	14	37°51′29.073"N	138°19′56.082"E	59	2020/8/17
NS3	Sado	20	38°03′40.021"N	138°21′24.059"E	180	2020/8/18
NS4	Sado	21	38°19′18.092"N	138°30′50.078"E	54	2020/8/19
NK	Nagano	17	36°49′31.943"N	138°09′12.600"E	954	2020/8/23
SK01	Shizuoka	18	34°44′14.712"N	138°04′45.155"E	28	2020/6/24
SH01	Shizuoka	5	34°46′25.273"N	137°44′46.751"E	26	2019/7/18
O1	Osaka	11	34°49′19.038"N	135°31′31.015"E	58	2020/9/19
Wk1	Wakayama	2	34°09′35.387"N	135°14′00.060"E	55	2007/6/5
T66	Tottori	10	35°15′20.535"N	134°18′41.410"E	732	2019/6/19
Tr77	Tottori	1	35°21′32.473"N	134°24′51.009"E	325	2019/6/25
Tr80	Tottori	4	35°28′19.983"N	134°08′30.564"E	103	2019/6/13
S58	Shimane	6	35°22′02.496"N	133°11′02.430"E	29	2019/7/2
Shi05	Shimane	1	35°26′10.222"N	132°52′46.819"E	1	2011/2/8
Shi19	Shimane	1	35°19′02.005"N	132°54′46.555"E	82	2011/3/24
Shi61	Shimane	3	35°14′42.757"N	132°42′41.024"E	218	2019/7/2
Shi66	Shimane	3	34°52′37.424"N	132°04′44.273"E	211	2019/7/3
Shi115	Shimane	2	35°05′47.491"N	132°50′17.790"E	557	2021/10/26
Shi122	Shimane	5	35°10′15.692"N	132°51′07.393"E	459	2021/10/27
Shi124	Shimane	3	34°45′13.348"N	132°03′11.846"E	232	2021/10/28
Oki29	Oki	1	36°06′07.167"N	133°07′08.198"E	155	2012/4/28
Oki93	Oki	1	36°16′15.216"N	133°19′18.700"E	322	2020/11/9
Oki94	Oki	1	36°16′46.773"N	133°20′10.283"E	40	2020/11/9
Oki95	Oki	2	36°16′30.320"N	133°19′59.059"E	87	2020/11/9
Oki98	Oki	2	36°13′10.191"N	133°11′54.473"E	79	2020/11/10
Oki99	Oki	1	36°15′57.053"N	133°16′16.343"E	351	2020/11/10
Oym15	Okayama	2	34°44′16.196"N	133°24′23.565"E	579	2019/7/10
Hrs19	Hiroshima	2	34°33′26.751"N	132°45′55.904"E	440	2019/7/10
Y21	Yamaguchi	6	34°09′44.356"N	132°03′24.612"E	164	2019/7/3
Y23	Yamaguchi	11	34°21′06.389"N	132°03′12.958"E	818	2019/7/9
Y25	Yamaguchi	1	34°11′23.850"N	131°45′53.735"E	384	2019/9/29
Y41	Yamaguchi	4	34°00′30.675"N	131°49′07.186"E	361	2015/12/1
Y45	Yamaguchi	2	34°16′57.320"N	131°38′59.413"E	222	2012/3/21
K11	Kagawa	14	34°07′21.572"N	134°06′08.099"E	482	2020/6/6
T11	Tokushima	20	33°47′57.617"N	134°25′50.688"E	141	2020/6/8
E6	Ehime	2	34°00′04.577"N	133°36′26.421"E	72	2019/10/26
E7	Ehime	1	33°52′20.516"N	133°09′46.280"E	75	2019/10/26
E8	Ehime	10	33°53′16.994"N	133°00′40.686"E	235	2019/10/26
E13	Ehime	1	33°35′12.645"N	132°46′30.000"E	146	2019/10/27
E14	Ehime	6	33°32′15.268"N	132°53′27.232"E	835	2019/10/27
E15	Ehime	13	33°29′36.996"N	132°32′14.629"E	80	2019/10/27
E18	Ehime	1	33°34′01.264"N	132°27′06.817"E	97	2019/10/28
E19	Ehime	3	33°28′43.280"N	132°18′01.548"E	189	2019/10/28
E20	Ehime	1	33°21′15.486"N	132°38′26.832"E	180	2019/10/28
Ko14	Kouchi	3	33°12′55.674"N	132°50′57.625"E	84	2019/10/30
F03	Fukuoka	8	33°47′11.903"N	130°35′17.664"E	53	2014/3/28
F20	Ohita	2	33°34′51.527"N	130°59′55.248"E	179	2010/10/10
F21	Fukuoka	2	33°30′01.728"N	131°09′44.100"E	144	2010/10/11
F147	Fukuoka	2	33°48′40.535"N	130°35′45.419"E	42	2015/5/5
F161	Fukuoka	2	33°47′46.211"N	130°36′21.492"E	56	2015/5/5
SG01	Saga	15	33°26′01.247"N	130°22′10.991"E	991	2009/11/20
Kg3	Kagoshima	1	31°18′43.379"N	130°31′39.791"E	572	2008/1/5
TS28	Tsushima	2	34°19′20.907"N	129°21′23.036"E	52	2016/3/8
TS03	Tsushima	6	34°09′06.487"N	129°12′36.710"E	62	2016/3/5
FI01	Fukue	11	32°40′28.676"N	128°42′31.031"E	183	2016/2/5
OK11	Okinawa	12	26°43′52.680"N	128°12′34.452"E	321	2010/2/12
	Niigata coastline (*Ligia* sp.)	4	37°54′27.251"N	138°59′58.559"E	4	2020/8/24

Site ID, sampling locality, sample size (n), site coordinates, elevation, and collection dates

If tectonic history had influenced *Ligidium*, we would expect that populations would exhibit divergence in relatively stable southwestern Japan since ancient times. In northeastern Japan, submerged until approximately 7 Ma, *Ligidium* would exhibit increased genetic differentiation after the uplifting of land from 3 to 7 Ma [16]. Second, the Quaternary glacial cycles might have caused the recent population size increase of *Ligidium* in cold northeastern Japan, as on Hokkaido. We evaluated these hypotheses.

## Results

### Phylogeny based on mtDNA

**Fig. 2:**
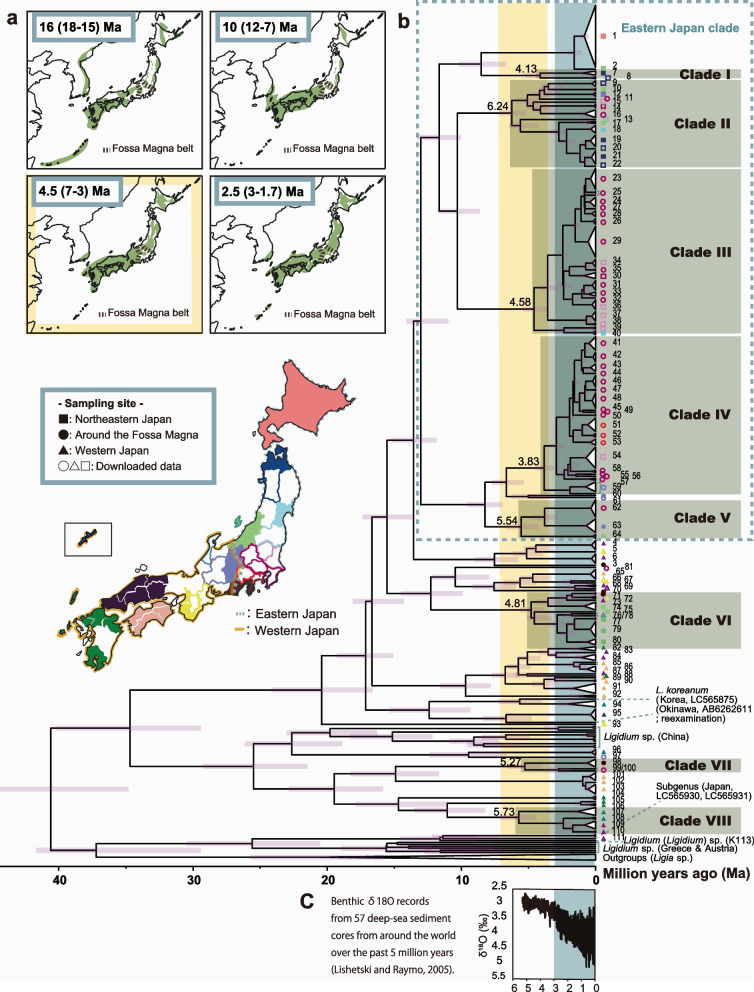
Bayesian phylogenetic tree of Japanese *Ligidium* with the geological history of the Japanese Islands and global climate change. **a** Paleogeographic map of the Japanese Archipelago. Colored areas are land. The map was constructed based on a geological survey of Japan [7]. **b** Bayesian phylogenetic tree. Colors for the operational taxonomic units (OTUs) match those of their sampling sites on the Japan map. See Table 2 for mitochondrial DNA (mtDNA)-OTU details. Colored borders and symbols indicate areas for which data were downloaded and downloaded data, respectively. We used the evolutionary rate of 1.68% per million years (3.36% divergence rate) for time estimation [19]. Clades I–VIII (dark boxes) have high posterior probabilities (> 0.99) and high bootstrap values (> 85%) in the maximum-likelihood (ML) tree (Fig. 3) and show characteristic branching. The numbers shown at tree nodes are ages. Horizontal lines are the node age ranges with 95% highest posterior density values. **c** Benthic δ^18^O records from 57 deep-sea sediment cores distributed worldwide over the past 5 million years [20]

**Fig. 3:**
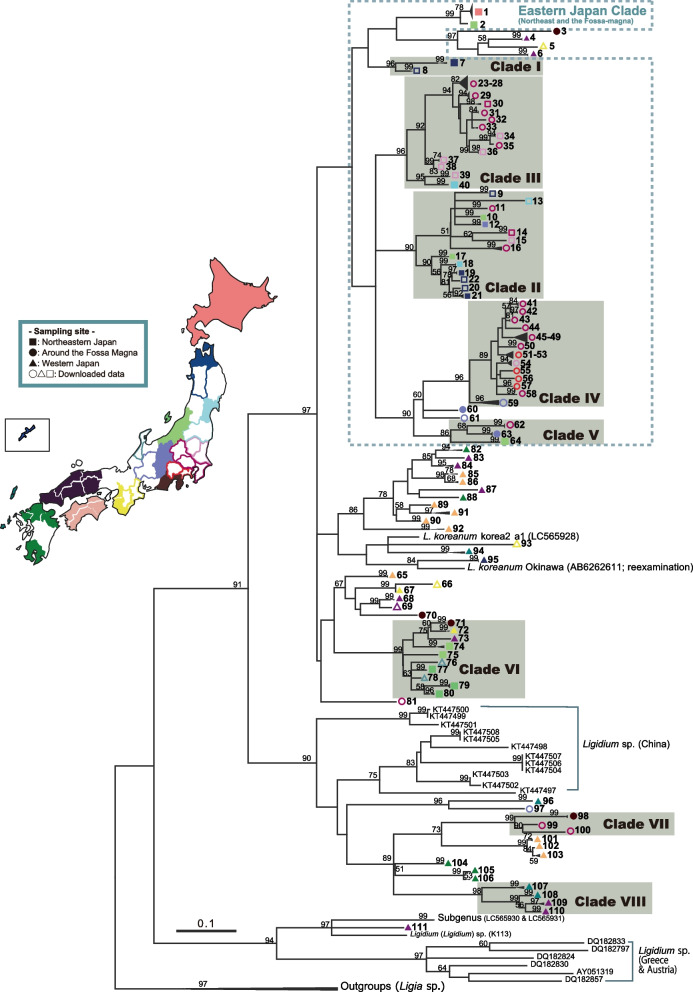
mtDNA-based ML tree. Numbers at tree nodes are bootstrap values from 1000 pseudo-replicates (≥ 50%). Number on horizontal line is branch length measured as the number of substitutions per site. Colors correspond to the archipelago map and OTUs. Colored borders and symbols indicate areas for which data were downloaded and downloaded data, respectively. See Table 2 for details on the OTUs

The aligned COI sequences in the dataset were each 496 bases long. Phylogenetic trees for the mtDNA sequences were constructed using Bayesian analysis and maximum likelihood (ML) methods (Figs. 2 and 3). We identified 111 mtDNA-operational taxonomic units (OTUs) (Table 2) in the phylogenetic trees, which were divided into eight monophyletic groups (Clades I–VIII) supported by high posterior probabilities (> 0.99) and bootstrap values (> 85%). Individuals of Aomori at the northern tip of Honshu constituted Clade I. Clades II and III are distributed over a wide area from the Fossa Magna belt to northeastern Japan. Clades IV and V live in the Fossa Magna belt and eastern Japan, and Clade VI inhabits western and eastern Japan beyond the Fossa Magna. Clade VII is distributed from the west to the east of the Fossa Magna. Individuals from western Japan and the islands constitute Clade VIII. The monophyletic groups were consistent with four morphological species (*Ligidium japonicum*, *L. koreanum, L.* sp. NIIGATA1, and *L.* sp. EHIME1), excluding some lineages in the clade of *L. koreanum* (Fig. S1). Most of the specimens in northeastern Japan and the Fossa Magna areas constituted a clade (eastern Japan clade; Fig. 2b). However, some specimens from the eastern areas were closely related to the western samples (e.g., Clades VI and VII). By contrast, western samples included deeper-branching lineages compared to the eastern ones (Fig. 2b) and did not constitute one western clade. Interestingly, the phylogenetic analyses showed that the divergence of multiple lineages (Clades I–VIII) occurred around 7–3.5 Ma and after 3 Ma throughout Japan (Fig. 2, Table 3). However, depth differed between eastern and western Japan.

**Table 2:** List of mitochondrial DNA (mtDNA)-operational taxonomic units (OTUs)

OTU ID	Sampling site ID	n	Reference	Haplotype (Accession number; n)	Population genetic analyses
1	Hokkaido (all sites), Aomori (48, 50, 54, 57), Niigata (32, 33)	Hokkaido (216), Aomori (16), Niigata (10)	this study and Harigai et al., 2020 [14]	See datails in Table S4	
2	32	1	Harigai et al., 2020 [14]	Niigata2 (LC534809; 1)	
3	Shizuoka	1	DataBank	SHIZU2 (LC496507; 1)	
4	Y23, T66, S58	20	this study	S5807 (LC711255; 1), S5809 (LC711256; 1), STY233 (LC711286; 5), STY231 (LC711285; 12), S5816 (LC711257; 1)	✓
5	Gose-shi, Nara	1	Yoshino & Kubota, 2022 [15]	Gose-shi_Nara1567_a1 (LC565854; 1)	
6	Shi61, Shi66, Shi122, Shi124, Tr80, Y21, Hrs19, Y41, Oym15	23	this study	Shi66-Che2084 (LC711271; 1), Tr-Sh-Ym-Hr (LC711296, 16), Ym21-Che2088 (LC712390; 1), Shi124_Che2298 (LC711267; 1), Shi122_Che2275 (LC711266; 1), Oym15_Che2182 (LC711252; 1) Shi112_Che2276 (LC711264; 2)	✓
7	50, 57	10	this study	A1^a^ (LC711144; 1), A2a (LC711145; 6), A2b (LC711146; 3)	✓
8	Aomori	2	Yoshino & Kubota, 2022 [15]	Fukaura-cho_a1(LC565849; 1), Fukaura-cho_Akita_b2 (LC565850; 1)	
9	ST01, 54, 57	4	this study and Yoshino & Kubota, 2022 [15]	ST0101 (LC711275; 1), A5a (LC711161; 1), A5b (LC711162; 1), Yuzawa-shi_a1 (LC566056; 1)	
10	32	6	Harigai et al., 2020 [14]	Niigata6a (LC534816; 5), Niigata6b (LC534815; 1)	
11	Saitama-Shi, Saitama	2	Yoshino & Kubota, 2022 [15]	Saitamashi1 (LC565995, LC565996; 2)	
12	NK	3	this study	NK0121 (LC711218; 1), NK0118 (LC711217; 1), NK0109 (LC711213; 1)	
13	Miharu-cho, Fukushima	1	Yoshino & Kubota, 2022 [15]	Miharu-cho_2 (LC565933; 1)	
14	Tsutsumi-cho, Gunma	3	Yoshino & Kubota, 2022 [15]	Tsutsumi-cho1 (LC566050, LC566051, LC566052; 3)	
15	Hitachi-shi, Ibaraki	1	Yoshino & Kubota, 2022 [15]	Hitachi-shi2 (LC565863; 1)	
16	Chiba/Saitama/Tokyo	5	Yoshino & Kubota, 2022 [15]	Itabashi-ku_b2 (LC565887; 1), Saitama1 (LC565912, LC565913; 2), Mt. Kanozan_h8 (LC565946; 1), Owasi_i9 (LC565990; 1)	✓
17	33	34	Harigai et al., 2020 [14]	Niigata4 (LC534813; 33), Niigata5 (LC534814; 1)	
18	ST02	8	this study	ST021 (LC711283; 4), ST0202 (LC711279; 1), ST0204 (LC711280; 1), ST0206 (LC711281; 1), ST0208 (LC711282; 1)	
19	55, 57, Amigasamori-Forest, Aomori	12	this study and Yoshino & Kubota, 2022 [15]	A4a (LC711155; 1), A4b (LC711156; 3), A4c (LC711157, 2), A4d (LC711158; 1), A4e (LC711159, 3), A4f (LC711160; 1), Amigasamori-Forest_a1 (LC565844; 1)	✓
20	Yuzawa-shi, Akita	1	Yoshino & Kubota, 2022 [15]	Yuzawa-shi_b2 (LC566057; 1)	
21	48, 50, 54, 55	33	this study	A3a (LC711147; 17), A3b (LC711148; 1), A3c (LC711149; 1), A3d (LC711150; 1), A3e (LC711151; 10), A3f (LC711152; 1), A3g (LC711153; 1), A3h (LC711154; 1)	✓
22	Dakigaeri-Valley, Akita	1	Yoshino & Kubota, 2022 [15]	Dakigaeri-Valley_a1 (LC565848; 1)	
23	Boso peninsula, Chiba	12	Yoshino & Kubota, 2022 [15]	Owasi_d4 (LC565985; 1), Chiba_Owasi1 (LC565982, LC565983, LC565988, LC565989, LC565991; 5), Owasi_c3 (LC565984; 1), Owasi_e5 (LC565987; 1), Owasi_Kanozan1 (LC565941, LC565944, LC565945; 3), Tozaki_3 (LC566043; 1)	✓
24	Boso peninsula, Chiba	6	Yoshino & Kubota, 2022 [15]	Naraihara_1 (LC565964; 1), Higashinosawa_4 (LC565857; 1), Okawazura1 (LC565976, LC565974; 2), Minamiboso-shi_a1 (LC565934; 1), Okawazura_d6 (LC565978; 1)	✓
25	Boso peninsula, Chiba	4	Yoshino & Kubota, 2022 [15]	Kiwadahata_i9 (LC565925; 1), Toyoka3 (LC566041, LC566039; 2)	
26	Hiroka, Chiba	4	Yoshino & Kubota, 2022 [15]	Hiroka1 (LC565860, LC565861, LC565862; 3), Hiroka_Chiba_2 (LC565859; 1)	
27	Tozaki, Chiba	1	Yoshino & Kubota, 2022 [15]	Tozaki_c5 (LC566046; 1)	
28	Mt. Nokogiriyama, Chiba	4	Yoshino & Kubota, 2022 [15]	Nokogiriyama_Chiba_1 (LC565948; 1), Nokogiriyama1 (LC565949, LC565951, LC565950; 3),	
29	Boso peninsula, Chiba	17	Yoshino & Kubota, 2022 [15]	Takataki_i9 (LC566033; 1), Kawayatu_k11^a^ (LC565909; 1), Kiwadahata2 (LC565927, LC565919, LC565920, LC565922; 4), Ishigami_Chiba_h8 (LC565883; 1), Kiwadahata_h8 (LC565924; 1), Kawayatu_f6 (LC565906; 1), Kiwadahata_e5 (LC565921; 1), Kiwadahata1 (LC565917, LC565918, LC565926; 3), Kiwadahata_g7 (LC565923; 1), Ishigami_c3 (LC565878; 1), Ishigami_k11 (LC565886; 1), Ishigami_i9 (LC565884; 1)	✓
30	Katori-shi, Chiba	3	Yoshino & Kubota, 2022 [15]	Katori1 (LC565904; 1), Katori-shi_e5 (LC565905; 1), Katori-shi_a1 (LC565903; 1)	
31	Boso peninsula, Chiba	6	Yoshino & Kubota, 2022 [15]	Sanmu-shi1 (LC565999, LC566000, LC566003, LC566004; 4), Sanmu-shi_e5 (LC566001; 1), Sanmu-shi_f6 (LC566002; 1)	✓
32	Sosa-shi, Chiba	1	Yoshino & Kubota, 2022 [15]	Chiba-Sosa c3 (LC566020; 1)	
33	Boso peninsula, Chiba	3	Yoshino & Kubota, 2022 [15]	Kawayatu_g7 (LC565907; 1), Kawayatu_h8 (LC565908; 1), Mutsuzawa-cho_b2 (LC565960; 1)	
34	Tochigi/Ibaraki	5	Yoshino & Kubota, 2022 [15]	Ii_Tochigi_2 (LC565865, LC565866; 2), Ii_Tochigi_1 (LC566011, LC566013; 2), Ii_Tochigi_a1 (LC565864; 1)	✓
35	Shimohino, Gunma	2	Yoshino & Kubota, 2022 [15]	Gunma_Shimohino2 (LC566007, LC566006; 2)	
36	Tsukuba-shi, Ibaraki	6	Yoshino & Kubota, 2022 [15]	Tsukuba1 (LC565952, LC565953, LC565954, LC565957, LC565958; 5), Tsukuba_c4 (LC565955; 1)	✓
37	Kashima-shi, Ibaraki	6	Yoshino & Kubota, 2022 [15]	Kashimajingu-Station1 (LC565897; 1), Kashima1 (LC565898, LC565901; 2), Kashima2 (LC565899, LC565902; 2), Kashimajingu-Station4 (LC565900; 1)	✓
38	Shimotsumashi, Ibaraki	2	Yoshino & Kubota, 2022 [15]	Shimotsuma-shi4 (LC566014; 1), Shimotsuma-shi2 (LC566012; 1)	
39	Kamiyakawauchi, Ibaraki	2	Yoshino & Kubota, 2022 [15]	Kamimiyakawauchi_a1 (LC565891; 1), Kamimiyakawauchi_b2 (LC565892; 1)	
40	ST01	7	this study	ST011 (LC711278; 5), ST0108 (LC711277; 1), ST0102 (LC711276;1)	✓
41	Boso peninsula, Chiba	8	Yoshino & Kubota, 2022 [15]	Tabina_3_b4 (LC566021, LC566022; 2), Okawazura1 (LC565973, LC565977, LC565979, LC565980; 4), Okawazura_a1 (LC565975; 1), Okawazura_g9 (LC565981; 1)	✓
42	Boso peninsula, Chiba	6	Yoshino & Kubota, 2022 [15]	Tozaki_f8 (LC566049; 1), Tozaki1 (LC566042, LC566044, LC566045, LC566048; 4), Tozaki_Chiba_d6 (LC566047; 1)	✓
43	Boso peninsula, Chiba	5	Yoshino & Kubota, 2022 [15]	Mt. Kanozan_a3 (LC565939; 1), Mt. Kanozan_d4 (LC565942; 1), Mt. Kanozan_b1 (LC565940; 1), Mt. Kanozan1 (LC565943, LC565947; 2)	✓
44	Kawayatu, Chiba	1	Yoshino & Kubota, 2022 [15]	Kawayatu_l12 (LC565910; 1)	
45	Midori-ku, Chiba	1	Yoshino & Kubota, 2022 [15]	Midori-ku_c3 (LC565932; 1)	
46	Boso peninsula, Chiba	7	Yoshino & Kubota, 2022 [15]	Ishigami_a1 (LC565876, LC565877, LC565880, LC565882, LC565885; 5), Ishigami_f6 (LC565881; 1), Ishigami_d4 (LC565879; 1)	✓
47	Takataki, Chiba	2	Yoshino & Kubota, 2022 [15]	Takataki3 (LC566025, LC566036; 2)	
48	Boso peninsula, Chiba	9	Yoshino & Kubota, 2022 [15]	Takataki1 (LC566026, LC566028, LC566030, LC566031, LC566032, LC566035; 6), Takataki2 (LC566027, LC566029; 2), Takataki_j10 (LC566034; 1)	✓
49	Sosa-shi, Chiba	1	Yoshino & Kubota, 2022 [15]	Sosa-shi_a1 (LC566019; 1)	
50	Tateyama, Chiba	2	Yoshino & Kubota, 2022 [15]	Tateyama1 (LC566037, LC566038; 2)	
51	Kawasaki-shi, Kanagawa	8	Yoshino & Kubota, 2022 [15]	Ikuta-Ryokuchi-Park_d4 (LC565870; 1), Kanagawa_Ikuta1 (LC565867, LC565868; 2), Kanagawa_h8 (LC5658734; 1), Ikuta-Kanagawa_e5 (LC565871; 1), Ikuta-Ryokuchi-Park_g7 (LC565873; 1), Ikuta2 (LC565869, LC565872; 2)	✓
52	Yokohama-shi, Kanagawa	3	Yoshino & Kubota, 2022 [15]	Yokohama1 (LC565969, LC565970; 2), Niharu_Yokohama_b2 (LC565971; 1)	
53	Fujisawa-shi, Kanagawa	4	Yoshino & Kubota, 2022 [15]	Shinbayashi-park_b2 (LC566015; 1), Shinbayashi-park_f6 (LC566016; 1), Shinbayashi-park_g7 (LC566017; 1), Shinbayashi-park_h8 (LC566018; 1)	
54	Chiba/Ibaraki	12	Yoshino & Kubota, 2022 [15]	Kitanakazuma_2b3 (LC565914, LC565916; 2), Kitanakazuma_a1 (LC565915; 1), Sakura-shi1 (LC565997, LC565998; 2), Narita-shi1 (LC565968, LC565967; 2), Narita-shi_a1 (LC565965; 1), Narita-shi_b2 (LC565966; 1), Funabashi1 (LC565851, LC565853, LC565852; 3)	✓
55	Akiruno-shi, Tokyo	1	Yoshino & Kubota, 2022 [15]	Akiruno-shi_b1 (LC565843; 1),	
56	Hamura-shi, Tokyo	1	Yoshino & Kubota, 2022 [15]	Hamura-shi_b1 (LC565856; 1)	
57	Takao-cho, Tokyo	2	Yoshino & Kubota, 2022 [15]	Takao-cho_2 (LC566023; 1), Takao-cho_a1 (LC566024; 1)	
58	Kanra-cho, Gunma	2	Yoshino & Kubota, 2022 [15]	Kanra-cho_Gunma_1 (LC565894; 1), Kanra-cho_Gunma_3 (LC565896; 1)	
59	Yamanashi/Shizuoka	7	Yoshino & Kubota, 2022 [15]	Kawazu-cho_1 (LC565911; 1), Yamanashi1 (LC566053, LC566054, LC566055; 3), Izukuni1 (LC565888, LC565889, LC565890; 3)	✓
60	NK	1	this study	NK0112 (LC711215; 1)	
61	Gifu	1	Yoshino & Kubota, 2022 [15]	Hirayu_Gifu_a1 (LC565858; 1)	
62	Gunma	10	Yoshino & Kubota, 2022 [15]	Gunma1 (LC565962, LC566008, LC565961, LC566010; 4), Nakanojo-machi_3 (LC565963; 1), Shimonita-cho_2 (LC566009; 1), Miyazawa-cho_1 (LC565935; 1), Miyazawa1 (LC565937, LC565938; 2), Miyazawa-cho_2 (LC565936; 1)	✓
63	NK	13	this study	NK0101 (LC711208; 1), NK0102 (LC711209; 1), NK0103 (LC711210; 1), NK0104 (LC711211; 1), NK011 (LC711214; 6), NK0106 (LC711212; 1), NK0114 (LC711216; 1), NK0122 (LC711219; 1)	✓
64	31	12	Harigai et al., 2020 [14]	Niigata3a (LC534811; 6), Niigata3b (LC534812; 5), Niigata3c (LC534810; 1)	
65	T11, S58	20	this study	S-T112^a^ (LC711284; 9), T111 (LC711289; 6), T113 (LC711292; 2), T1101 (LC711287; 1), T1102 (LC711288; 1), T1116 (LC711291; 1)	✓
66	Gose-shi, Nara	1	Yoshino & Kubota, 2022 [15]	Gose-shi_b2 (LC565855; 1)	
67	Wakayama	2	this study	Wakayama1_bu36 (LC711301; 2)	
68	Tr77, Tr80	2	this study	Tr77_Che2258 (LC711294; 1), Tr80_Che2293 (LC711295; 2)	
69	Okayama-Bizen 1	1	Yoshino & Kubota, 2022 [15]	Bizen-shi_Okayama_1 (LC565846; 1)	
70	SK01	1	this study	SK0119 (LC711274; 1)	
71	SH01	4	this study	SH011 (LC711262; 2), SH012 (LC711263; 2)	
72	O1	11	this study	O011 (LC711236; 9), O012 (LC711237; 2)	✓
73	Shi124	2	this study	Shi5-19_Che2199 (LC711270; 2)	
74	31, 32	18	Harigai et al., 2020 [14]	Niigata7a (LC534817; 14), Niigata7b (LC534820; 1), Niigata7c (LC534819; 1), Niigata7d (LC534818; 1), Niigata8 (LC534821; 1)	
75	30	14	Harigai et al., 2020 [14]	Niigata9 (LC534822; 14)	
76	Notojima, Ishikawa	1	Yoshino & Kubota, 2022 [15]	Notojima-Island_Ishikawa_a1 (LC565972; 1)	
77	NS2	13	this study	NS021 (LC711220; 12), NS0213 (LC711222; 1)	✓
78	Anamizu, Ishikawa	1	Yoshino & Kubota, 2022 [15]	Anamizu-cho_Ishikawa_a1 (LC565845; 1)	
79	NS3	20	this study	NS031 (LC711226; 7), NS032 (LC711231; 6), NS0301 (LC711223; 1), NS0306 (LC711224; 1). NS0309 (LC711225; 1), NS0314 (LC711227; 1), NS0316 (LC711228; 1), NS0317 (LC711229; 1), NS0318 (LC711230; 1)	✓
80	NS4	21	this study	NS041 (LC711233; 17), NS042 (LC711234; 2), NS0408 (LC711232; 1), NS0413 (LC711235; 1)	✓
81	Chichibu, Saitama	1	Yoshino & Kubota, 2022 [15]	Chichibu-shi_Saitama_3 (LC565847; 1)	
82	F03, SG01	16	this study	F031 (LC711187; 6), SG01 (LC711258; 5), SG03 (LC711260; 5)	✓
83	Shi124	1	this study	Shi124_Che2302 (LC711269; 1)	
84	Y23, Y25, Y41, Y45	5	this study	Y23-22–1 (LC711303; 2), Ym41_Che2229 (LC711307; 1), Ym41_Che2230 (LC711308; 1), Ym45_Che2244 (LC711309; 1)	✓
85	E18, E19	4	this study	Eh18_Che2314 (LC711181; 1), Eh19_Che2322 (LC711183; 2)	
86	E15, E20	13	this study	E151 (LC711173; 9), E152 (LC711175; 2), E1509 (LC711172; 1), Eh20_Che2313 (LC711184; 1)	✓
87	Oym15	1	this study	Oym15_Che2183 (LC711253; 1)	
88	F20	2	this study	Fuk21_Ag231 (LC711196; 2)	
89	E14	2	this study	Eh14_Che2320 (LC711180; 2)	
90	Ko14	3	this study	Ko14_Che2327 (LC711203; 1), Ko14_Che2315 (LC711202; 2)	
91	E6, E7, E8	7	this study	Eh6_Che2309 (LC711185; 2), Eh7_Che2310 (LC711186; 1), E0802 (LC711165; 1), E0803 (LC711166; 1), E0805 (LC711167; 1), E0806 (LC711168; 1)	✓
92	K11	11	this study	K1123 (LC711199; 1), K111 (LC711197; 8), K112 (LC711198; 2)	✓
93	Isato, Mie	1	Yoshino & Kubota, 2022 [15]	Isato-cho_Mie_a1 (LC565875; 1)	
94	TS03, TS28	10	this study	TS031 (LC711299; 5), TS0309 (LC711298; 1), TS0307 (LC711297; 1), TS031 (LC711299; 3), Tsm28_Che21^a^ (LC711300; 2)	✓
95	OK11	12	this study	OK02 (LC711241; 2), OK03 (LC711242; 3), OK04 (LC711243; 2), OK05 (LC711244; 2), OK0104 (LC711238; 1), OK0110 (LC711239; 1), OK0112 (LC711240; 1)	✓
96	FI01	11	this study	FI011 (LC711190; 3), FI012 (LC711191; 5), FI013 (LC711192; 2), FI0104 (LC711189; 1)	✓
97	Kamioka, Gifu	1	Yoshino & Kubota, 2022 [15]	Kamioka-cho-do_Gifu_a1 (LC565893; 1)	
98	SK01, SH01, Hamamatsu (Shizuoka)	20	this study and Yoshino & Kubota, 2022 [15]	Sahama-cho_Hamamatsu_1 (LC565994; 1), SHIZU1 (LC496506; 1), SK012 (LC711272; 5), SK011 (LC711273; 12), SH0102 (LC711261; 1)	✓
99	Kanra-cho, Gunma	1	Yoshino & Kubota, 2022 [15]	Kanra-cho_Gunma_2 (LC565895; 1)	
100	Toyoka, Chiba	1	Yoshino & Kubota, 2022 [15]	Toyoka_Chiba_b2 (LC566040; 1)	
101	E8	6	this study	E0801 (LC711164; 1), E0807 (LC711169; 1), E0810 (LC711170; 1), E0812 (LC711171; 1), E088 (LC711163; 2)	✓
102	T11	1	this study	T1115 (LC711290; 1)	
103	E13, E14, E15, E19, Kumakogen-cho (Ehime)	8	this study and Yoshino & Kubota, 2022 [15]	Eh14_Che2318 (LC565929,1; LC711179, 2), Eh14_Che2317 (LC711178; 1), Eh19_Che2312 (LC711182; 1), E1510 (LC711174; 1), Eh14_Che2080 (LC711177; 1), Eh13_Che2079 (LC711176; 1)	✓
104	F03, F161, F147	6	this study	F032 (LC711188; 4), Fuk147_Che11 (LC711193; 1), Fuk147_Che12 (LC711194; 1)	✓
105	SG01	5	this study	SG03 (LC711260; 5)	
106	Kg3, F20	3	this study	Kg3_bu51 (LC711201; 1), Fuk20_Ag229 (LC711195; 2)	
107	Oki93, Oki95, Oki98	6	this study	Oki93-Che2140 (LC711246; 1), Oki94-95_Che2143 (LC711247; 2), Oki95_Che2145 (LC711248; 1), Oki98_Che2147 (LC711249; 1), Oki98_Che2148 (LC711250; 1)	✓
108	Oki29, Oki99	2	this study	Oki99_Che2151 (LC711251; 1), Oki29_bu241 (LC711245; 1)	
109	Shi124, Shi05, Shi115, Y23, Y25, Sugiura (Shimane)	7	this study and Yoshino & Kubota, 2022 [15]	Shi124_Che2300 (LC711268; 1), Y232 (LC711302; 2), Ym25_Che778 (LC711304; 1), Shi115_Che2306 (LC711265; 2), Sagiura_Shimane_1^a^ (LC565992; 1)	✓
110	Y41	2	this study	Ym41_Che2227 (LC711305; 1), Ym41_Che2228 (LC711306; 1)	
111	T66	1	this study	T6613 (LC711293; 1)	

^a^Sequences removed from the population genetic analysis

**Table 3:** Divergence times of the major Clades (Clade I-VIII) and 95% HPD

Clade	Estimate	95% HPD
Clade I	4.13	2.96–5.31
Clade II	6.24	5.23–7.18
Clade III	4.58	3.63–5.46
Clade IV	3.83	3.06–4.53
Clade V	5.54	4.29–6.71
Clade VI	4.81	3.90–5.67
Clade VII	5.27	3.93–6.59
Clade VIII	5.73	4.40–7.09

**Fig. 4:**
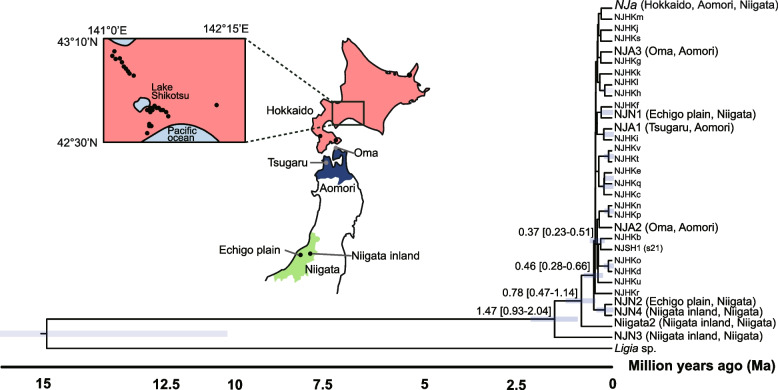
Bayesian tree of mtDNA-OTU 1 and 2 with divergence time estimation. Haplotype *NJ*a is shared among Hokkaido, Aomori, and Niigata. Haplotypes Niigata2 and NJN1, 3, and 4 are from Niigata (Niigata inland area [site ID 32] and Echigo Plain [site ID 33]) We used an evolutionary rate of 1.68% per million years (3.36% divergence rate) for time estimation [19]. Horizontal lines are node age ranges with 95% highest posterior density values

In Japan, mtDNA-OTU 1 and 2 diverged recently, although samples were found across the northern (Hokkaido and Aomori) and central (Niigata) areas of Japan (Fig. 2b). We constructed a Bayesian tree and estimated divergence time to investigate the phylogenetic relationships of these samples (Fig. 4). Some southerly Niigata haplotypes were older than the Hokkaido and Aomori haplotypes. The oldest Niigata haplotype diverged ~ 1.5 Ma, whereas the haplotypes from Hokkaido and Aomori, located in the northernmost part of Japan, diverged ~ 0.4 Ma.

### Estimated historical demographic patterns inferred from mtDNA

We conducted population genetic analyses on 44 mtDNA-OTUs (northeastern Japan, 8; Fossa Magna belt, 19; western Japan, 17) based on the following criteria: close distribution, the presence of multiple haplotypes, and more than five individuals (Table 2). Mismatch distribution analysis detected population expansion and an expansion parameter (τ) was estimated to identify the generations in which the population size expanded (see Table S1 for calculation results). Smooth and unimodal frequency distributions indicated a recent, sudden population expansion in 17 mtDNA-OTUs, distributed as follows: northeastern Japan, 5; Fossa Magna belt, 5; western Japan, 7 (Fig. S2). Bimodal distributions were produced for 14 mtDNA-OTUs (northeastern Japan, 2; Fossa Magna belt, 7; western Japan, 5), showing that they might have experienced two expansions. Tajima’s D [21] and Fu’s Fs [22] neutrality tests assess the degree of deviation from neutral evolution and the presence or absence of natural selection. We found negative values for 35 mtDNA-OTUs, indicating population expansion (northeastern Japan, 7; Fossa Magna belt, 16; western Japan, 12), for both or either of the tests (Table 4). For the mtDNA-OTUs that exhibited expansion trends based on the mismatch distribution graph and neutrality tests, the expansion dates were 60 ~ 30 ka for nine mtDNA-OTUs (northeastern Japan, 3; Fossa Magna belt, 3; western Japan, 3), and 20 ~ 10 ka for another 10 mtDNA-OTUs (northeastern Japan, 2; Fossa Magna belt, 2; western Japan, 6) (Table 4).

**Table 4:** Population genetic analysis results

MtDNA OTU ID	Sampling site ID	Site	Sample size	Number of base pairs	Fu's Fs test	Fu's Fs test *p*-value	Tajima's D	Tajima's D *p*-value	τ (under the sudden expansion model)	τ (under the spatial expansion model)	T (thousand years)
7	50, 57	■	9	615	0.85	0.548	0.99	0.90	0.76	0.76	7
19	55, 57, Amigasamori-Forest, Aomori	■	13	573	-2.33^*^	0.045	0.32	0.65	2.59	2.59	26
21	48, 50, 54, 55	■	33	573	-3.87^**^	0.008	-1.70*	0.02	0.98	0.99	10
40	ST01	■	7	600	-0.24	0.246	-1.36	0.08	1.93	1.41	13–19
18	ST02	■	8	600	-0.61	0.286	-0.67	0.29	4.96	2.74	26–47
77	NS2	■	13	616	-0.54^*^	0.020	-1.15	0.16	2.97	0.14	1–28
79	NS3	■	20	617	-1.97	0.138	-0.32	0.42	4.74	3.78	35–44
80	NS4	■	21	592	5.04	0.971	-0.15	0.49	3.00	13.8	29–134
34	Tochigi/Ibaraki	□	5	658	0.469	0.53	1.57	0.97	2.79	2.67	23–24
36	Tsukuba-shi, Ibaraki	□	6	658	6.50	0.99	-1.30	0.08	3.00	21.1	26–184
37	Kashima-shi, Ibaraki	□	6	658	-0.99	0.12	-0.68	0.33	1.74	2	15
62	Gunma	○	10	636	-3.34^*^	0.02	-0.79	0.23	4.06	3.02	27–37
54	Chiba/Ibaraki	○	12	606	0.28	0.55	-0.018	0.53	11.2	1.76	17–106
23	Boso peninsula, Chiba	○	12	599	-1.76	0.11	-0.510	0.33	2.86	2.73	26–27
24	Boso peninsula, Chiba	○	6	601	0.16	0.44	-0.24	0.45	8.07	8	77
29	Boso peninsula, Chiba	○	17	641	-3.66^*^	0.03	-0.93	0.18	4.85	3.15	28–44
31	Boso peninsula, Chiba	○	6	641	0.38	0.51	0.34	0.65	3.3	2.71	24–29
41	Boso peninsula, Chiba	○	8	604	2.09	0.85	0.17	0.60	11.2	9.99	95–106
42	Boso peninsula, Chiba	○	6	639	-0.19	0.26	-1.23	0.11	1.7	1.45	13–16
43	Boso peninsula, Chiba	○	5	627	-0.33	0.30	0.29	0.67	4.03	4.03	37
46	Boso peninsula, Chiba	○	7	646	-0.92	0.07	-1.24	0.12	0.73	0.73	6–7
48	Boso peninsula, Chiba	○	9	648	1.72	0.82	-0.58	0.29	0.67	7.57	6–67
16	Chiba/Saitama/Tokyo	○	5	611	1.82	0.74	-0.077	0.54	11.0	10.2	96–104
51	Kawasaki-shi, Kanagawa	○	8	614	-0.04	0.45	0.77	0.81	8.3	7.76	73–78
63	NK	●	13	561	-0.88	0.31	-0.60	0.30	0.0	9.5	0–97
98	SK01, SH01, Hamamatsu (Shizuoka)	●	20	563	3.56	0.93	0.58	0.76	10.5	10.8	107–110
59	Yamanashi/Shizuoka	○	7	658	2.78	0.88	-0.275	0.41	38.1	12.7	111–333
72	O1	▲	11	596	3.56	0.941	-0.16	0.45	3.00	5.63	29–54
4	Y23, T66, S58	▲	20	504	-1.13	0.164	-1.63^*^	0.04	0.87	0.58	7–10
6	Shi61, Shi66, Shi122, Shi124, Tr80, Y21, Hrs19, Y41, Oym15	▲	23	618	-3.14^**^	0.010	-2.09^**^	0.006	0.00	1.72	0–16
109	Shi124, Shi05, Shi115, Y23, Y25, Sugiura (Shimane)	▲	6	617	2.65	0.881	0.77	0.79	17.4	17.2	160–162
84	Y23, Y25, Y41, Y45	▲	5	614	2.11	0.785	1.34	0.92	23.3	23.2	217–218
107	Oki93, Oki95, Oki98	▲	6	673	1.42	0.683	1.07	0.89	1.84	24.6	16–210
65	T11, S58	▲	19	596	-1.28	0.173	-0.94	0.19	0.78	0.79	8
91	E6, E7, E8	▲	7	581	1.54	0.693	1.02	0.89	33.8	32.8	324–334
101	E8	▲	6	583	-0.28	0.337	0.48	0.67	7.16	7.03	69–72
103	E13, E14, E15, E19, Kumakogen-cho (Ehime)	▲	8	623	0.19	-1.143	-1.63^*^	0.03	0.63	0.63	6
86	E15, E20	▲	13	562	4.87	0.977	-0.54	0.32	0.00	12.10	0–124
92	K11	▲	11	606	9.08	0.998	-0.25	0.42	0.00	33.00	0–313
82	F03, SG01	▲	16	593	12.9	1.000	2.66	1.00	23.5	23.6	228
96	FI01	▲	11	584	-0.11	0.441	-0.05	0.51	1.47	1.44	14
104	F03, F161, F147	▲	6	592	0.76	0.63	-0.057	0.47	4.35	3	31–42
94	TS03, TS28	▲	8	579	-0.07	0.443	-1.06	0.16	6.14	4.26	42–61
95	OK11	▲	12	582	-2.63^*^	0.029	-0.61	0.29	2.15	2.15	21

To calculate the degree of deviation from neutral evolution and determine the presence or absence of natural selection, we used Tajima’s D [21] and Fu’s Fs [22] neutrality tests. For Tajima’s D and Fu’s Fs statistics, negative values indicate population expansion, a value of zero indicates a constant size, and positive values indicate a decreased population. Statistics were computed from 10,000 bootstrap pseudo-replicates within 95% confidence intervals (*P < 0.05, **P < 0.01). The estimated expansion parameter (τ) represents the generation in which the population size expanded. The time in millions of years (T) since demographic expansion was estimated for populations that exhibited an expansion tendency in the neutrality tests with the formula T = τ/2μk, where μ is the evolutionary rate per million years and k is the number of sequence base-pairs. This study used an evolutionary rate of 0.087 for *Ligidium* [14], calculated using the post-LGM warming as a calibration point. Site symbols indicate sampling areas as follows: ■, northeastern Japan; ●, around the Fossa Magna; and ▲, western Japan. Colored borders and symbols indicate areas for which data were downloaded and downloaded data, respectively. Column 1 lists the sampling site IDs corresponding with the Site IDs

### Results of genome-wide analyses

#### Single nucleotide polymorphisms (SNPs) and loci calling

After trimming the raw reads, we used the denovo_map.pl pipeline and the populations program in Stacks for assembly, aligning, and SNP calling of paired-end reads [23]. No SNPs were shared by > 70% of all individuals. Therefore, we selected likely phylogenetically close individuals based on the mtDNA results and conducted repeated analyses to determine how to divide them to maximize the SNPs and members (Fig. S3, Table S3). We were able to define two groups: western and northeastern Japan. We selected 67 individuals in the northeastern group and 10 in the western group (Table S3). SNP calling for the outgroup (*Ligia*) was not successful. After complete filtering, we identified 67 individuals sharing 79 loci and 961 SNPs in northeastern Japan and 10 individuals sharing 301 loci and 1567 SNPs in western Japan (Table S3A). SNP calling remained unsuccessful for all individuals even when we used ipyrad [24]; therefore, grouping was conducted based on the Stacks results. More SNPs were called in ipyrad than in Stacks: 134 loci and 3319 SNPs in northeastern Japan, and 747 loci and 12,866 SNPs in western Japan (Table S3B). However, with only 24 loci and 644 SNPs, there were insufficient SNPs to conduct analyses between the eastern and western populations.

### Nuclear DNA-based clusters

**Fig. 5:**
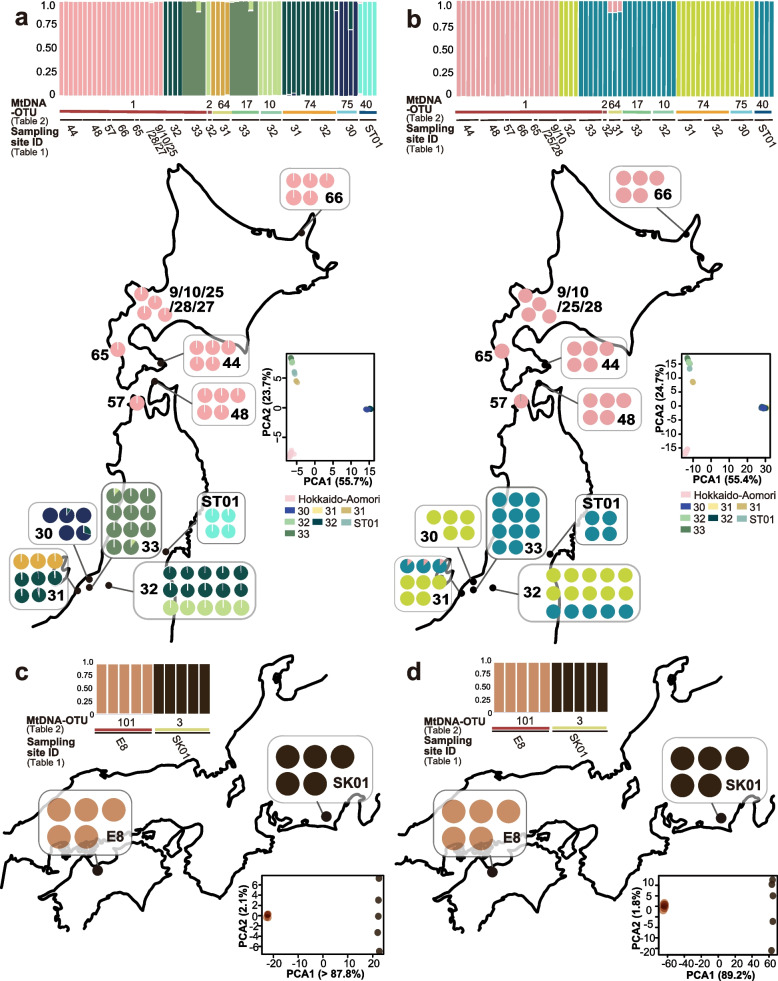
Genome-wide analysis results. Genetic structure of the populations determined using Bayesian cluster analysis: **a** Stacks for northeastern populations, **b** ipyrad for northeastern populations, **c** Stacks for western populations, and **d** ipyrad for western populations. For each result of the genome-wide single-nucleotide polymorphism (SNP) cluster analysis, we depict the structure (top), structure map (left), and principal component analysis (PCA) plot (right). PCA is a dimensionality reduction technique that identifies correlations and patterns in a dataset. ﻿Same-colored circles indicate membership of the same cluster in Structure and the same sampling site in PCA. Each vertical line represents an individual, and the length is the posterior probability of membership in each cluster. The lists below the genetic structure data contain mtDNA and sampling site information (see Table 1)

We performed Bayesian cluster analysis to assess population genetic structure for genome-wide data analyses. For the SNPs called by Stacks, the posterior probability values for the number of clusters (K) did not converge between K = 5 and 12, so the simulations were rerun 20 times for each K value from 5 to 12. After recalculating, because Ln(P) remained high for several values, the number of clusters was estimated based on the delta K method [25]. K was calculated to be 11, and the main nuclear clusters in each region were consistent with the geographical distribution in northeastern Japan (Fig. 5a). With the ipyrad SNPs, the nuclear clusters converged on 3 (Fig. 5b). Although the samples of mtDNA-OTU 1 exhibited slight genetic differences based on the mtDNA phylogenetic trees, they belonged to multiple nuclear clusters. Based on principal component analysis (PCA), the northeastern nuclear clusters were divided into three major clusters separated by distribution area. However, on Mt. Kakuda (Site ID 31) and in the Niigata inland area (Site ID 32), some clusters coexisted. This coexistence was also observed in the mtDNA-OTUs at the Niigata sites. We found only one nuclear cluster in Hokkaido, where there was only one mtDNA-OTU. In the western Japan populations, 10 samples were separated into two nuclear clusters based on their structure and the PCA results, which were consistent with the geographic distribution and mtDNA groups (Fig. 5c, d). However, this may be an effect of the small sample size.

Although all nuclear clusters were consistent with the geographic distributions, according to the mtDNA-OTU type, several individuals of the Niigata nuclear clusters possessed the same or genetically similar mtDNA haplotype as the Hokkaido individuals (Fig. 5a, b). Meanwhile, individuals from the Hokkaido–Aomori nuclear cluster did not have the Niigata mtDNA-OTU haplotype, indicating mtDNA introgression from the Hokkaido–Aomori population to the Niigata population in one direction. According to the morphological examination, the specimens from Hokkaido–Aomori were *L. japonicum*, but an undescribed species, *Ligidium* sp. NIIGATA1, was present in the Niigata specimens that had experienced introgression (Fig. S1). This suggests that intra-clade mtDNA introgression occurred within and between species.

### Pairwise Fst

Pairwise Fst values for the genome-wide SNPs revealed a population structure in the nuclear clusters (Table 5). Fst, which represents the degree of genetic differentiation among populations, ranges from 0 to 1, with an Fst > 0.25 indicating high genetic differentiation [26]. We obtained high and significant Fst values for northeastern and western Japan including the mtDNA-OTU and nuclear clusters in which gene introgression had occurred (Table5, Fig. 5).

**Table 5:** Fst values between populations of Japanese *Ligidium*

(A) (i)							
Fst in northeastern Japan from SNPs by stacks	Hokkaido-Aomori	Site ID 31 & 32	Site ID 33	Site ID 32	Site ID 31	Site ID 30	Site ID ST01
Hokkaido-Aomori	0						
Site ID 32 & 31	0.927^**^	0					
Site ID 33	0.854^**^	0.928^**^	0				
Site ID 32	0.832^**^	0.913^**^	0.463^**^	0			
Site ID 31	0.860^**^	0.933^**^	0.780^**^	0.720^**^	0		
Site ID 30	0.914^**^	0.452^**^	0.909^**^	0.872^**^	0.904^*^	0	
Site ID ST01	0.855^**^	0.930^**^	0.733^**^	0.626^**^	0.790^*^	0.898^**^	0
(A) (ii)							
Fst of northeastern Japan from SNPs by ipyrad	Hokkaido-Aomori	Site ID 31 & 32	Site ID 33	Site ID 32	Site ID 31	Site ID 30	Site ID ST01
Hokkaido-Aomori	0						
Site ID 32 & 31	0.952^**^	0					
Site ID 33	0.888^**^	0.940^**^	0				
Site ID 32	0.882^**^	0.935^**^	0.458^**^	0			
Site ID 31	0.905^**^	0.953^**^	0.800^**^	0.750^**^	0		
Site ID 30	0.945^**^	0.481^**^	0.922^**^	0.904^**^	0.931^**^	0	
Site ID ST01	0.899^**^	0.947^**^	0.717^**^	0.661^**^	0.797^**^	0.922^**^	0
(B) (i)							
Fst of western Japan from SNPs by stacks		Site ID E8			Site ID SK01		
Site ID E8		0					
Site ID SK01		0.910^*^			0		
(B) (ii)							
Fst of western Japan from SNPs by ipyrad		Site ID E8			Site ID SK01		
Site ID E8		0					
Site ID SK01		0.935^**^			0		

Pairwise Fst values were calculated for the RAD-seq dataset to test for the presence of population structures supported by the cluster analysis. SNPs were called using (i) Stacks and (ii) ipyrad. (A) Fst values for comparisons among the seven populations of northeastern Japan. (B) Fst values between the two populations of western Japan. Statistical significance was determined from 1000 restored extractions

^*^P < 0.05

^**^P < 0.01. Values between groups with mtDNA introgression are shown in bold

## Discussion

### Influence of the tectonic history of the Japanese Archipelago on the phylogenetic divergence of domestic *Ligidium*

We found high phylogenetic diversity in *Ligidium* in Japan, indicating that this genus is highly diversified. In particular, the phylogenetic divergences of several clades occurred around 7–3.5 Ma (Fig. 2, Table 3). Between 12 and 7 Ma, large areas of the northeastern part of the Japanese Archipelago and the Fossa Magna belt sank below sea level, but at 7–3 Ma, tectonic movement raised the land (Fig. 2a) [7, 27]. The northeastern and southwestern Japan arcs meet at the Fossa Magna region. Thus, the divergence seen at 7–3.5 Ma might have been triggered by uplift and exposure of the northeastern Japan arc.

Divergence of Clades II–VII (Fig. 2b) suggested that uplift and land exposure enabled the dispersal of Clades II–VII and their phylogenetic differentiation. Although *Ligidium* is distributed nationwide, only these six lineages spread on (Clades IV, V, and VII) or beyond (Clades II, III, and VI) the Fossa Magna and diverged at the time of the uplift and exposure. Clade I bifurcated in Aomori at the northern tip of Honshu, far from the Fossa Magna zone, but its bifurcation may also be attributed to the tectonic uplift of northeastern Japan. It is also possible that the phylogenetic divergence of Clades II, III, and V was due to the uplift of northeastern Japan and global cooling of the area 7 ~ 5.4 Ma. The divergence suggests a westward migration of *Ligidium*. Global ocean surface temperature records indicate that cooling was more marked at higher latitudes [28]. Thus, it is likely that migration occurred from high-latitude northeastern Japan to the Fossa Magna belt.

The nuclear and mtDNA results showed that Clade VI diverged from the western Japan lineage after 4.8 Ma (Table 3) and then dispersed to northeastern Japan with uplift of the northeastern Japan arc. Clade VI contained individuals from the nuclear cluster of the Niigata area (Site IDs 30, 31, and 32) in the nuclear ML tree (Fig. S1h). This nuclear cluster diverged from its ancestors earlier, but its internal clusters are immature, suggesting more recent dispersal to its current habitat.

Clade VIII, which encompassed *Ligidium* sp. CHUGOKU1, was found in an area far from the Fossa Magna zone (Figs. 2 and 3), suggesting that its divergence was not related to the uplift event. Clade VIII represents the divergence of *Ligidium* populations on Oki Island and Honshu. Oki Island repeatedly sank to the bottom of the sea but became terrestrial by 7–3 Ma, which corresponds to the time of this divergence [29].

Similarly, the formation history of the Japanese Archipelago may underlie why many species and genetic lineages in southwestern Japan diverged earlier from their ancestors than those in northwestern Japan. During the middle Miocene (16–10 Ma), the northeastern Japan arc comprised an archipelagic sea due to the expansion and large-scale subsidence of the Japan Sea [16, 29]. By contrast, the southwestern Japan arc west of the Fossa Magna was already elevated and had been mountainous for 15 million years [16, 29]. Older lineages are presumed to have inhabited southwestern Japan due to the ancient stable land areas. The clade belonging to the Chinese lineage might have differentiated when the Japanese Archipelago was connected to the continent and inhabited the southwestern Japan arc when it was separated from the Eurasian continent by the formation of the Japan Sea. This historical background may also explain the divergence depth similarities between the western Japanese lineage and continental Chinese *Ligidium*. According to genome-wide SNPs, genetic segregation of the western and eastern Japanese populations likely had the same cause. Similar segregations have been seen in other taxa [18, 30, 31].

### Influence of quaternary glacial cycles on domestic *Ligidium*

The Northern Hemisphere ice sheets expanded and glacial–interglacial contrasts were enhanced after 2.5 Ma [20]. Our phylogenetic tree suggested that mtDNA-OTU 1 and 2 divergences between Hokkaido–Aomori and Niigata occurred repeatedly after the beginning of the Quaternary glacial cycle around 2.5 Ma (Fig. 4). Our data indicated a southward migration of *L. japonicum* from northeastern populations during the glacial cycle. Additionally, the conservation of the nuclear cluster indicated that the migration began recently.

Because warming is assumed to have increased the genetic diversity of *L. japonicum* in Hokkaido [14] after the LGM, it is possible that climate oscillations during the glacial cycle led to the diversification of Japanese *Ligidium*. Population genetic analyses of mtDNA sequences re-aligned according to their geographic distribution and mtDNA-OTU type showed that 34 of 45 OTUs underwent population expansions (Table 4). Although only nine groups were significant in neutrality tests, the mismatch distribution graphs and mtDNA phylogenetic tree results showed trends of population expansions (Fig. 2, Fig. S2, Table 4), so we discussed 34 groups. The northeastern Japanese lineages could be attributed to the Quaternary glacial cycle. In the northeastern populations, seven of eight groups had negative values in neutrality tests (Tajima’s D [21]; Fu’s Fs [22]). The estimated expansion dates of almost all these groups are within the last 50,000 years. The LGM period was the most recent major climatic event. As in the Hokkaido population [14], expansions between 20 and 10 ka might have been influenced by post-LGM warming. In addition, marine isotope stage (MIS) 4 (71–57 ka) was as cold as the LGM. The expansions between 30 and 60 ka correspond to MIS 3, the interval between the LGM and MIS 4. Analysis of pollen fossils in the sediments of Lake Nojiri, located in the middle of the Japanese Archipelago, indicated that MIS 3 was warmer than MIS 4 [32]. The mtDNA phylogenetic trees also suggested that *Ligidium* was affected by the glacial cycle. Multiple synchronous divergences in geographically distant regions occurred after 3 Ma (Fig. 2).

In contrast to northeastern Japan, many western mtDNA-OTUs, such as *L. koreanum* and *Ligidium* sp. CHUGOKU1, yielded positive values in neutrality tests. However, the following groups had negative values and expansions within the last 100 ka: OTUs from islands (mtDNA-OTU 94, 95, and 96), two OTUs from the Chugoku region of western Honshu (mtDNA-OTU 4 and 6), one OTU from the southernmost of the four main islands of Japan, Kyushu (mtDNA-OTU 104), and two OTUs from the smallest of the four main islands of Japan, Shikoku (mtDNA-OTU 65 and 103).

The LGM and MIS 4 glacial periods may have affected the Chugoku groups as in northeastern Japan. These Chugoku groups of *L. japonicum* (Fig. S1, Table S4) suggest past migrations from the Fossa Magna. *L. japonicum* was affected by the LGM cooling in Hokkaido [14]. Moreover, the Hokkaido–Aomori population moved southward with the glacial cycle (Fig. 4). *L. japonicum* populations in northeastern Japan, including Hokkaido, were influenced by the glacial periods and might have been sensitive to climate change during those periods.

A population expansion of the mtDNA-OTU 4 clade occurred 10–7 ka. This population might have escaped from the sea level rise caused by the Jomon transgression. The Jomon transgression is associated with the combination of rapid glacial retreat and the slow uplift of the continental margin by hydro isostasy after the LGM, which peaked ca. 6 ka [33]. The mtDNA-OTU 103 population expansion corresponded to this time. The mtDNA-OTU 65 expansion date was slightly earlier than that of the Jomon transgression but coincided with the postglacial period. The population expansion in Kyushu occurred after MIS 4, suggesting the influence of warming after a cold period. Alternatively, the population might have been affected by the arrival of humans—archaeological evidence indicates that paleolithic people arrived in Japan from the continent 40–30 ka, after which their population expanded [34]. Kyushu faces Tsushima Island, a route of human expansion.

### Discrepancies between the mtDNA and genome-wide analysis results

Classification often uses mtDNA barcodes as molecular tools. However, we identified mtDNA introgressions in individuals belonging to different nuclear clusters. Our results suggest that in *Ligidium*, hybridization could occur among individuals with a high degree of genetic differentiation (Fst > 0.88; Fig. 5, Fig. S1, Table 5). Therefore, species identification based on mtDNA may lead to misidentification. However, the mtDNA-OTU and nuclear clusters in which no gene introgression had occurred were consistent (Fig. S1). Also, genetic differences between the *Ligidium* sp. EHIME1 and the northeastern Japan populations were so large that SNP sampling was unsuccessful, again agreeing with the molecular data and morphological observations.

## Conclusions

The modern phylogeography of Japanese *Ligidium* was determined by archipelago formation and climate change, and indicated highly endemic genomic evolution in each habitat. Although genetic structures affected by old events were conserved in the nuclear genome, mtDNA recorded changes due to relatively recent events such as glacial cycles. Thus, different timescale reconstructions may be possible depending on the molecular markers selected. Slowly evolving gene regions may enable us to trace back thousands of years of history. Because *Ligidium* lives throughout the Northern Hemisphere [8], its nuclear genome may provide insight into the history of Northern Hemispheric tectonic dispersal. Although the oldest fossil of *Ligidium* is Paleogenic [35], its DNA sequence may enable evaluation of older eras. Fossils of Oniscidea are rarely preserved because of the biochemical properties of the exoskeleton. Thus, they have likely lived since more ancient times than indicated by the fossil record [35]. Furthermore, as demonstrated in this study, a rapidly evolving genetic region may signal much younger geological events, such as volcanic eruptions.

Our results revealed high genetic diversity in domestic *Ligidium*. The intrageneric diversity of this taxon may exceed the number of species currently reported, and further work is needed to combine molecular data and morphological characters. Finally, evolution of the genome in the populations examined in this study highlights the importance of considering genetic structure in biological conservation taking also into account that most of the populations represent endemic taxa.

## Materials and methods

### Sample collection

We surveyed *Ligidium* populations in Japan. We collected 828 *Ligidium* specimens from 97 sites and sequenced 721 samples (Fig. 1, Table 1). Samples were preserved in 70–99.5% ethanol in 2-mL microtubes at 4 °C or room temperature.

### DNA extraction, amplification, and sequencing

Genomic DNA was isolated from the muscles of the abdomen and legs with a DNA Mini Kit (Qiagen, Germantown, MD, USA). PCR amplification was conducted using the primer pair LCO-1490 and HCO-2198 [36]. Amplification and cycling conditions were as in a previous work [14]. Sequencing was conducted with an ABI 3130 Genetic Analyzer (Applied Biosystems, Waltham, MA, USA). Sequences were checked and assembled using MEGA7 [37]. Mitochondrial gene locus (CO1) sequences for 721 individuals were determined and aligned using ClustalW [38].

### Phylogenetic analyses using mtDNA

Phylogenetic trees were constructed with the Bayesian analysis tool BEAST2 [39] and the ML method in MEGA7 [37]. The Bayesian tree analysis conditions were the strict clock model and a constant population size for 50 million step runs with sampling every 100 steps. The results were checked with Tracer v1.7.1 [40] after discarding a 10% burn-in, summarized with TreeAnnotator v1.10.4 (http://beast.community/index.html), and visualized with FigTree v1. 4.4 [41]. Because we focused on the events of the exposure of the northeastern Japan arc and convergence of eastern and western Japanese islands uniting until 3 Ma, we used the evolutionary rate calibrated by the events before this date. Previous studies using such events as calibration points showed that arthropods have a divergence rate of 3.0% [6, 19]. Thus, we used the rate of tenebrionid beetles (divergence rate 3.36%, clock rate 1.68%) used in many phylogenetic studies. Its rate estimation was performed with a calibration point of the formation of the mid-Aegean trench at 9–12 Ma. We selected the Tamura 3-parameter [42] + G + I evolutionary model in MEGA 7 for the ML tree. The ML tree’s nodal support values were obtained with 1000 bootstrap pseudo-replicates. We used sequences of subgenus *Ligidium* (Austria, Greece, and Japan) and, as an outgroup, relatively closely related *Ligia* spp. (Japan, Hawaii, unknown area) belonging to the family Ligiidae of the order Isopoda in which the genus *Ligidium* was also assigned until recently when the family was found to be polyphyletic [43]. Samples of the subgenus *Ligidium* (*Ligidium*) spp. came from Kagawa, Shikoku, Japan. We also downloaded data for the following *Ligidium* species: *L. ryukyuense* (AB626261; this specimen was misidentified and closely related to *L. koreanum*, based on reexamination in this study) collected on Okinawa Island, *Ligidium* sp. SHIZU2 (LC496507) and *Ligidium* sp. SHIZU1 (LC496506) collected in Shizuoka, a prefecture central to the Pacific Ocean side of Honshu, 213 sequences of individuals sampled mainly in the regions around Tokyo, Japan, that were previously published by Yoshino and Kubota [15], *Ligidium* species from Europe, and Oniscidea sp. collected in Shanghai, China. Table S4 lists the accession numbers for the downloaded data. Data from Yoshino and Kubota [15] were also used in the population genetics analyses.

Historical demographic patterns were examined by mismatch distribution analysis [44] and neutrality tests with Arlequin 3.5.1.2 [45]. Mismatch distribution analysis was conducted with spatial and sudden expansion models to detect population expansions and estimate the expansion parameter (τ) representing the generation marking the start of the population expansion. In the analyses, smooth and unimodal frequency distributions indicate an expanding population [45]. The time since the expansion in generations (t) can be estimated with the formula t = τ/2uk, where u is the evolutionary rate per generation and k is the number of sequence base-pairs [46]. We estimated the time since the demographic expansion in millions of years (T) with the formula T = τ/2μk, where μ is the evolutionary rate per million years. We used an evolutionary rate of 0.087 for *Ligidium* [14]. We performed Tajima’s D [21] and Fu’s Fs [22] as neutrality tests to calculate the degree of deviation from neutral evolution and determine the presence or absence of natural selection. Negative values for Tajima’s D and Fu’s Fs statistics for a population indicate that the population experienced expansion. Statistics and 95% confidence intervals were computed with 10,000 bootstrap pseudo-replicates.

### RAD-seq

We performed RAD-seq analysis to search for SNPs in individuals from Niigata and Hokkaido obtained in previous studies [14], two regions in northern Japan (Aomori and Sendai), and two regions in western Japan (Shizuoka and Sendai). Tables 1 and S1 list the samples used, and the sampling sites are shown in Fig. 1c. Genomic DNA was isolated from almost whole-body tissues with a DNA Mini Kit (Qiagen). Libraries for RAD-seq were prepared with EcoRI and BglII restriction enzymes [47, 48]. The library was sequenced with 150 + 150 bp paired-end reads in one lane of an Illumina HiSeqX instrument (Illumina, San Diego, CA, USA) by Macrogen (Seoul, South Korea). Raw reads were trimmed using Trimmomatic-0.39 [49] with the following parameters: ILLUMINACLIP: adapter.fasta:2:30:10:keepBothReads, SLIDINGWINDOW: 4:15, CROP: 132, HEADCROP: 2, and MINLEN: 130. Sequences are available at the DNA Data Bank of Japan (DDBJ) Sequence Read Archive (DRA014204). We used two pipeline programs to call the SNPs: denovo_map.pl provided by Stacks [23] and ipyrad [24]. Following a previous work [50], we varied the combinations of the denovo_map.pl parameters as follows: (n, M) = (2, 1), (3, 2), (4, 3), (5, 6), and selected (n, M) = (2, 1), which called the most SNPs. We used Stacks’ populations program to analyze populations of individual samples, calculate population genetics statistics, and export data in various output formats for analysis. PLINK v1.90b6.18 [51] was used for data handling. Alleles with a frequency of < 1% and sites with > 50% heterozygosity were removed. Only SNPs shared by more than 80% of the individuals were retained. With ipyrad, loci with frequencies of > 50% heterozygosity were removed, and SNPs shared by more than 70% of the local populations were retained. We retained SNPs shared by at least two individuals and filtered out individuals that did not have 80% of all SNPs using TASSEL 5 [52]. After filtering with TASSEL 5, we used PGDSpider to convert the vcf files for other analyses [53].

### Genetic structure analysis

We tested the ability of Structure v. 2.3.4 [54] to determine the genetic structure of populations using Bayesian cluster analysis. Ten simulations were run, with the burn-in period and Markov chain Monte Carlo iterations set to 10^5^ and 10^6^, respectively. The maximum value of K was determined based on the mtDNA results and geographical distribution. For the Structure analysis, one SNP was randomly sampled from each locus to avoid the effect of linkage disequilibrium. The python script vcf_single_snp.py (radseq/vcf_single_snp.py at master · pimbongaerts/radseq · GitHub) was used to obtain the one SNP datum from ipyrad, and drawings were created using the R package pophelper [55]. In addition, PCA was performed to visualize the genetic differences among populations using the adegenet package in R [56, 57]. We obtained pairwise Fst values for the RAD-seq dataset using Arlequin 3.5.1.2. Fst values were used to test population structure, supported by cluster analysis, and statistical significance was based on 1000 restored extractions.

### Statistics and reproducibility

Tables 1, 2, and S1 list summaries of the sample size. Statistical analyses were conducted for distribution analysis, neutrality tests, phylogenetic tree constructions, structure analysis, and calculations of Fst. The methods used for reproducibility checks are described in the corresponding methods section. A *P*-value < 0.05 was considered indicative of statistical significance, and the results were reproducible through access to the genetic data (see “Availability of data and materials”).

### Supplementary Information

**Additional file 1.**

## Data Availability

mtDNA sequences were deposited in the DDBJ. RAD-seq sequences are available at the DDBJ Sequence Read Archive (DRA014204). The result outputs, scripts of SNP data (Software: https://doi.org/10.5281/zenodo.7393599), and a list of mtDNA accession numbers (Supplemental information: https://doi.org/10.5281/zenodo.7393601) can be downloaded from DRYAD (https://doi.org/10.5061/dryad.zkh1893bw) without any permissions.

## Abbreviation

ka: Thousand years ago
LGM: Last glacial maximum
Ma: Million years ago
MIS: Marine isotope stage
ML: Maximum likelihood
mtDNA: Mitochondrial DNA
OTU: Operational taxonomic unit
PCA: Principal component analysis
RAD-seq: Restriction-site-associated DNA sequencing
SNP: Single-nucleotide polymorphism
