# Supplementary material for: History of the terrestrial isopod genus Ligidium in Japan based on phylogeographic analysis
Source: BMC Ecol Evol. 2023 Aug 7;23:38. doi: 10.1186/s12862-023-02144-8 (PMC10405464; doi:10.1186/s12862-023-02144-8)
Supplement: Supplementary file 1 — Additional file 1. [file 12862_2023_2144_MOESM1_ESM.pdf]

Supplementary Information

Supplementary Figures 2

Fig. S1. Illustrations of six morphological species identified in this study from microscopic preparations of the male pleopod 2 endopod. 2

Fig. S2. Results of mismatch distribution analysis (Schneider and Excoffier, 1999). 5

Fig. S3. Grouping from SNP calling based on mitochondrial DNA (mtDNA) tree clades. 20

Supplementary Tables... 21

Table S1 All results of the population genetic analyses. 21

Table S2 List of Japanese *Ligidium* individuals in genome-wide analyses. 29

Table S3 Results of SNPs calling for each group. 30

Table S4 List of following data: accession numbers of all mitochondrial haplotypes including outgroups, RAD-seq data ID, morphology, sampling locations. 32

Supplementary Reference... 59

Supplementary Figures

Fig. S1. Illustrations of six morphological species identified in this study from microscopic preparations of the male pleopod 2 endopod.

Although the taxonomy of the genus *Ligidium* is confusing and requires further study, we made morphological observations of the male pleopod 2 endopod used in previous studies of the genus. Pleopod 2 endopodites were removed from 208 male specimens under a stereomicroscope (SZX16, Olympus, Tokyo, Japan). These were placed in Hoyer's mounting medium on slides, covered with a coverslip, and observed under a microscope (Eclipse E400, Nikon, Tokyo, Japan). The specimens were divided into six morphological groups. Two had morphological characteristics identical to those of *Ligidium japonicum* and *L. koreanum*, which have been reported in Japan (Nunomura, 1983). The other four species could be undescribed or unreported species in Japan. Given that a comprehensive study is needed to determine the taxonomic status of the species, these groups were tentatively treated as species to clarify the discussion. In addition, we reexamined a specimen of *L. ryukyuense* (AB626261) and found similar morphological characteristics to *L. koreanum*. The morphological characteristics of each group are described below:

Ligidium japonicum: Apex of the pleopod 2 exopodite is U-shaped with several denticles on the inner margin. A beak-shaped dent is located on the outer margin and located apart from the denticles.

Ligidium koreanum: Apex of the pleopod 2 exopodite is pointed. One to three denticles are located on the inner margin, and a beak-shaped dent is located on the outer margin. The denticles and dent are located close to each other.

***Ligidium* sp_NIIGATA1**: Several denticles are found on the inner margin, but there

is no beak-shaped dent at the apex of the pleopod 2 endopodite.

***Ligidium* sp_CHUGOKU1**: A beak-shaped dent with a small dent is located on the outer margin at the anterior part of the pleopod 1 endopodite. There is no denticle on the apex.

***Ligidium* sp_EHIME1**: Apex of the pleopod 1 endopodite is tapered. A beak-shaped dent is located on the outer margin, but there is no denticle at the apex.

***Ligidium* sp_FUKUE1**: The pleopod 2 exopodite is narrow with a large trapezoidal projection on the outer margin.

Male pleopod 2 endopodites. (a) *Ligidium japonicum,* (b) *L. koreanum,* (c) *Ligidium* sp_NIIGATA1, (d) *Ligidium* sp_CHUGOKU1, (e) *Ligidium* sp_EHIME1, (f) *Ligidium* sp_FUKUE1. (g) Phylogenetic relationships of each morphological group in the mitochondrial and nuclear maximum-likelihood (ML) tree. (h) ML phylogenetic tree based on single nucleotide polymorphisms (SNPs) called using ipyrad constructed using RAxML-NG (Kozlov et al., 2019). ModelTest-NG (Darriba et al., 2020) was used to select models for RAxML-NG. The python script ascbias.py (https://github.com/btmartin721/raxml_ascbias) removed invariant sites. Support values were obtained from bootstrapping with 1000 replicates. (i) Mitochondrial ML trees are shown in Fig. 3.

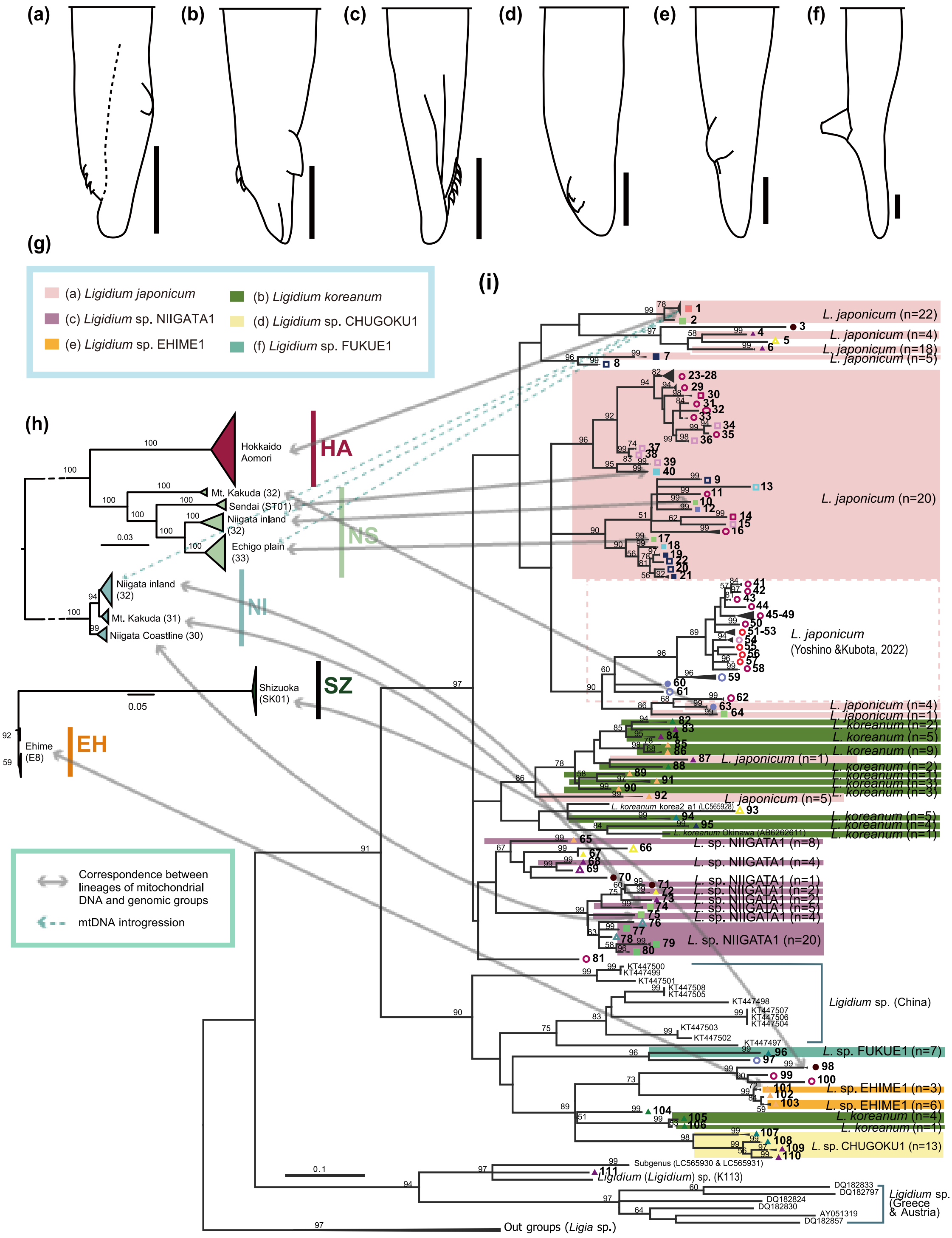

(a)
(b)
(c)
(d)
(e)
(f)
(g)
(a) Ligidium japonicum
(b) Ligidium koreanum
(c) Ligidium sp. NIIGATA1
(d) Ligidium sp. CHUGOKU1
(e) Ligidium sp. EHIME1
(f) Ligidium sp. FUKUE1
(h)
Hokkaido Aomori
HA
Mt. Kakuda (32)
Sendai (ST01)
Niigata inland (32)
Echigo plain (33)
NS
0.03
Niigata inland (32)
Mt. Kakuda (31)
Niigata Coastline (30)
NI
Shizuoka (SK01)
SZ
0.05
Ehime (E8)
EH
Correspondence between lineages of mitochondrial DNA and genomic groups
mtDNA introgression
(i)
L. japonicum (n=22)
L. japonicum (n=4)
L. japonicum (n=18)
L. japonicum (n=5)
L. japonicum (n=20)
L. japonicum (Yoshino &Kubota, 2022)
L. japonicum (n=4)
L. japonicum (n=1)
L. koreanum (n=2)
L. koreanum (n=5)
L. koreanum (n=9)
L. japonicum (n=1)
L. koreanum (n=2)
L. koreanum (n=1)
L. koreanum (n=3)
L. koreanum (n=3)
L. japonicum (n=5)
L. koreanum korea2 a1 (LC565928)
L. koreanum (n=5)
L. koreanum (n=4)
L. koreanum (n=1)
L. koreanum Okinawa (AB626281)
L. sp. NIIGATA1 (n=8)
L. sp. NIIGATA1 (n=4)
L. sp. NIIGATA1 (n=1)
L. sp. NIIGATA1 (n=2)
L. sp. NIIGATA1 (n=2)
L. sp. NIIGATA1 (n=5)
L. sp. NIIGATA1 (n=4)
L. sp. NIIGATA1 (n=20)
KT447500
KT447499
KT447501
KT447508
KT447505
KT447498
KT447507
KT447506
KT447504
KT447503
KT447502
KT447497
Ligidium sp. (China)
L. sp. FUKUE1 (n=7)
L. sp. EHIME1 (n=3)
L. sp. EHIME1 (n=6)
L. koreanum (n=4)
L. koreanum (n=1)
L. sp. CHUGOKU1 (n=13)
Subgenus (LC565930 & LC565931)
Ligidium (Ligidium) sp. (K113)
DQ182833
DQ182797
DQ182824
DQ182830
AY051319
DQ182857
Ligidium sp. (Greece & Austria)
0.1
Out groups (Ligia sp.)

Fig. S2. Results of mismatch distribution analysis (Schneider and Excoffier, 1999).

Mismatch distribution analysis was conducted with the spatial expansion model and the sudden expansion model to detect population expansions and estimate the expansion parameter (τ) representing the generation in which the population size had started to expand. In the analysis, smooth frequency distributions with a single mode indicate expanded populations. The histogram shows the frequency of each pairwise differences in the sample, and the line shows the expected frequency under the models.

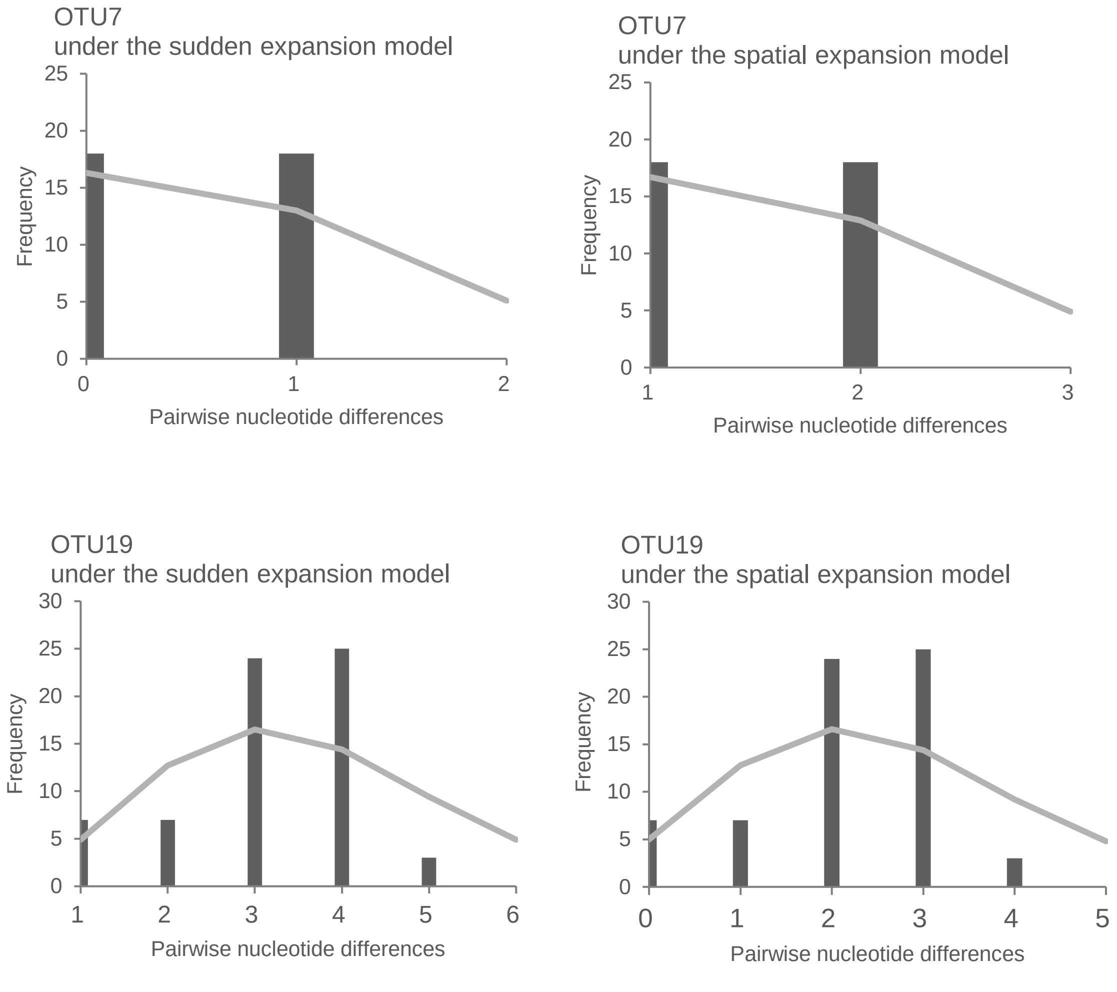

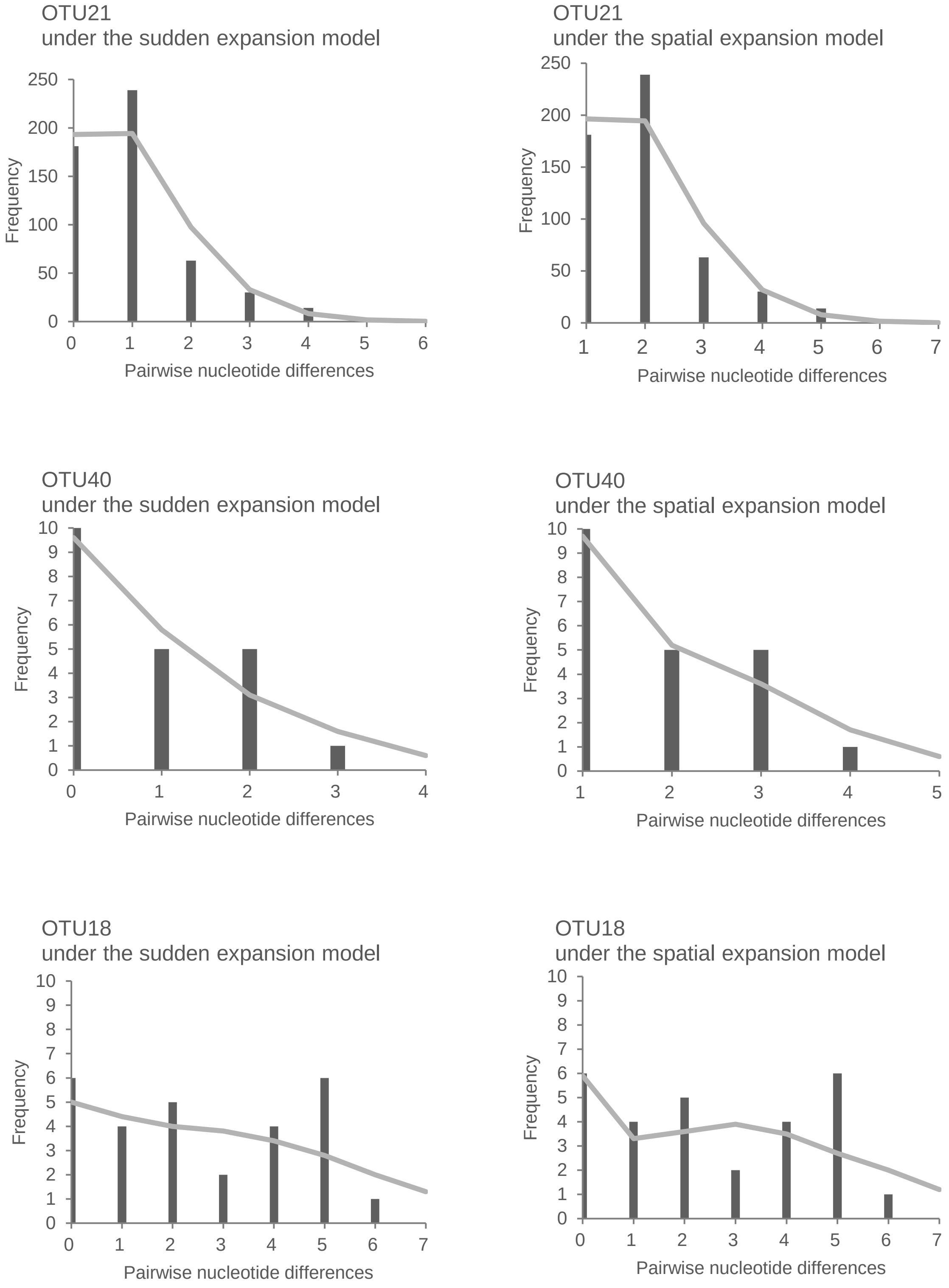
OTU21
under the sudden expansion model
Frequency
Pairwise nucleotide differences
OTU21
under the spatial expansion model
Frequency
Pairwise nucleotide differences
OTU40
under the sudden expansion model
Frequency
Pairwise nucleotide differences
OTU40
under the spatial expansion model
Frequency
Pairwise nucleotide differences
OTU18
under the sudden expansion model
Frequency
Pairwise nucleotide differences
OTU18
under the spatial expansion model
Frequency
Pairwise nucleotide differences

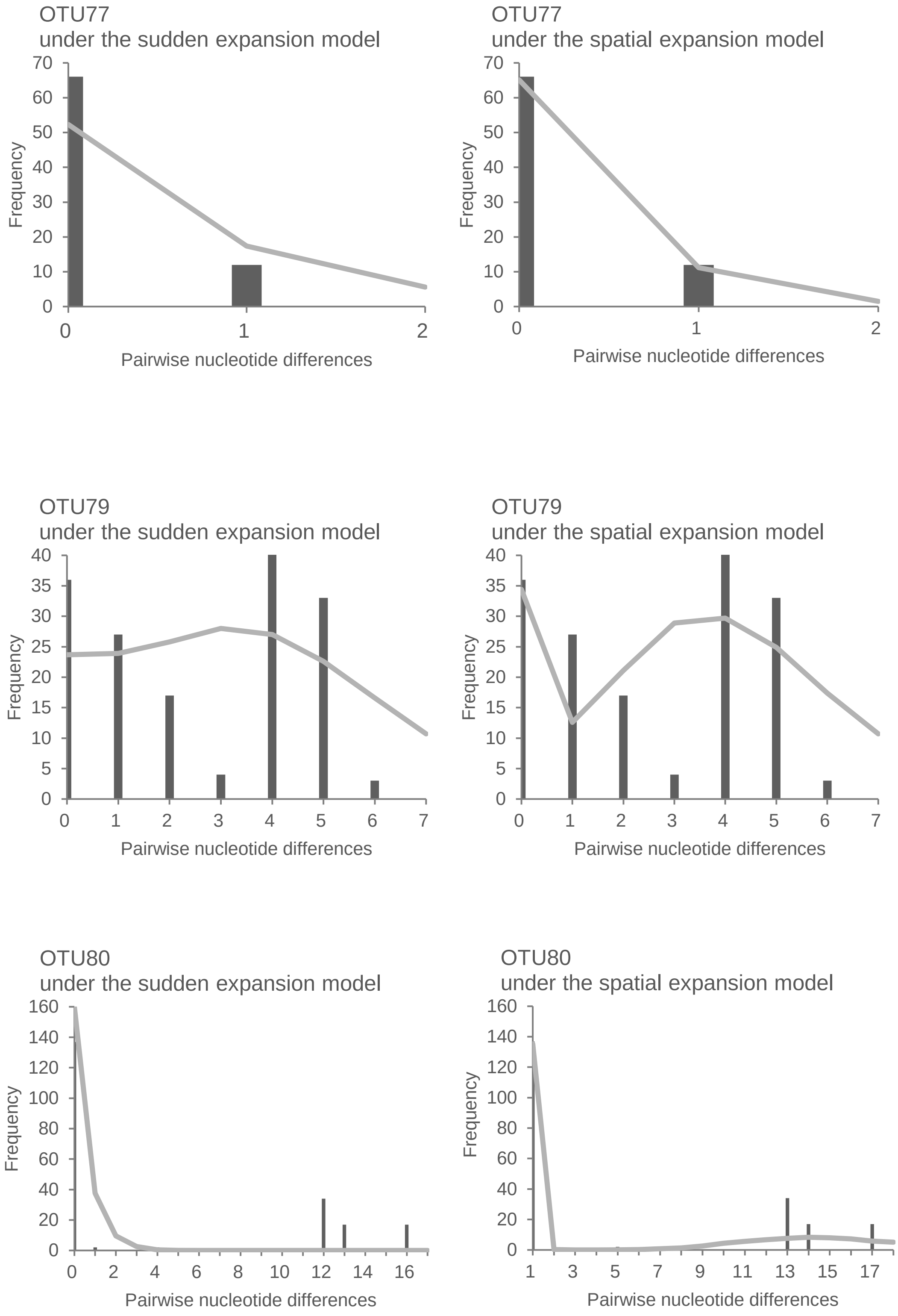
OTU77
under the sudden expansion model
Frequency
Pairwise nucleotide differences
OTU77
under the spatial expansion model
Frequency
Pairwise nucleotide differences
OTU79
under the sudden expansion model
Frequency
Pairwise nucleotide differences
OTU79
under the spatial expansion model
Frequency
Pairwise nucleotide differences
OTU80
under the sudden expansion model
Frequency
Pairwise nucleotide differences
OTU80
under the spatial expansion model
Frequency
Pairwise nucleotide differences

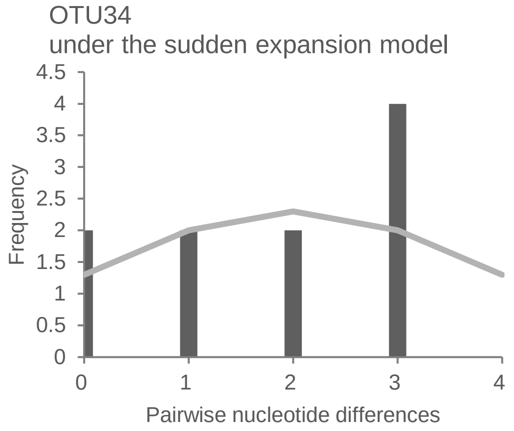
OTU34
under the sudden expansion model
Frequency
Pairwise nucleotide differences
0 0.5 1 1.5 2 2.5 3 3.5 4 4.5
0 1 2 3 4

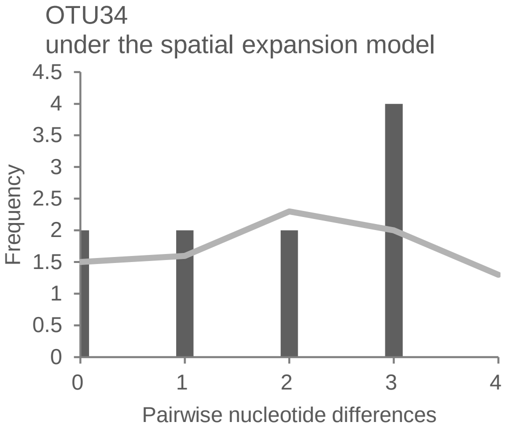
OTU34
under the spatial expansion model
Frequency
Pairwise nucleotide differences
0 0.5 1 1.5 2 2.5 3 3.5 4 4.5
0 1 2 3 4

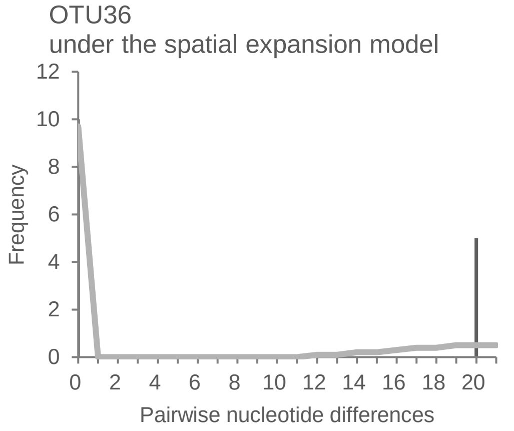
OTU36
under the spatial expansion model
Frequency
Pairwise nucleotide differences
0 2 4 6 8 10 12
0 2 4 6 8 10 12 14 16 18 20

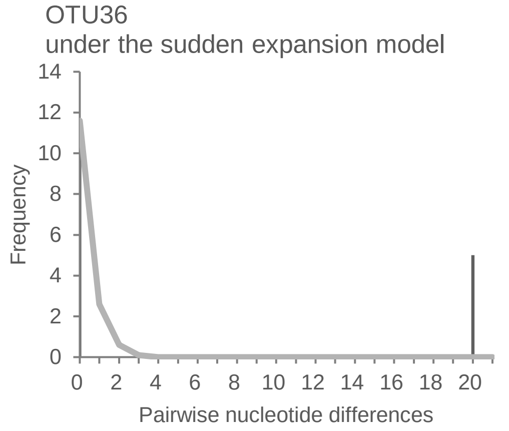
OTU36
under the sudden expansion model
Frequency
Pairwise nucleotide differences
0 2 4 6 8 10 12 14
0 2 4 6 8 10 12 14 16 18 20

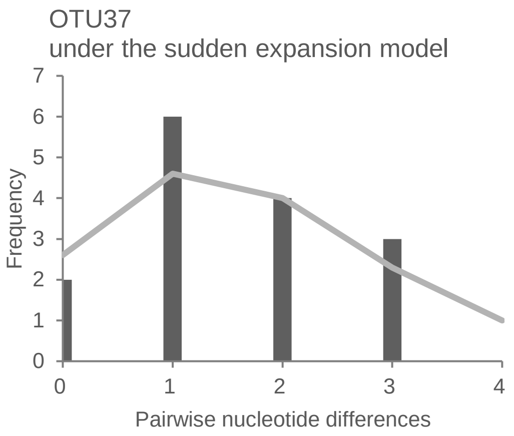
OTU37
under the sudden expansion model
Frequency
Pairwise nucleotide differences
0 1 2 3 4 5 6 7
0 1 2 3 4

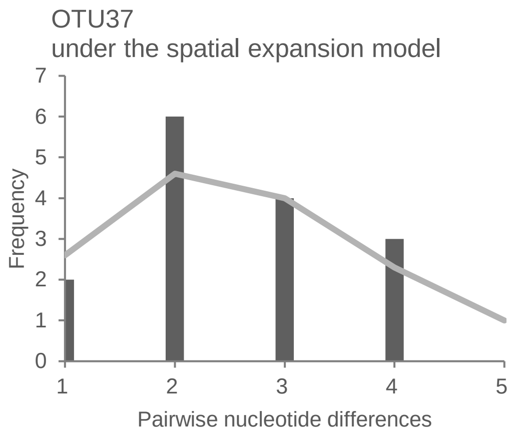
OTU37
under the spatial expansion model
Frequency
Pairwise nucleotide differences
0 1 2 3 4 5 6 7
1 2 3 4 5

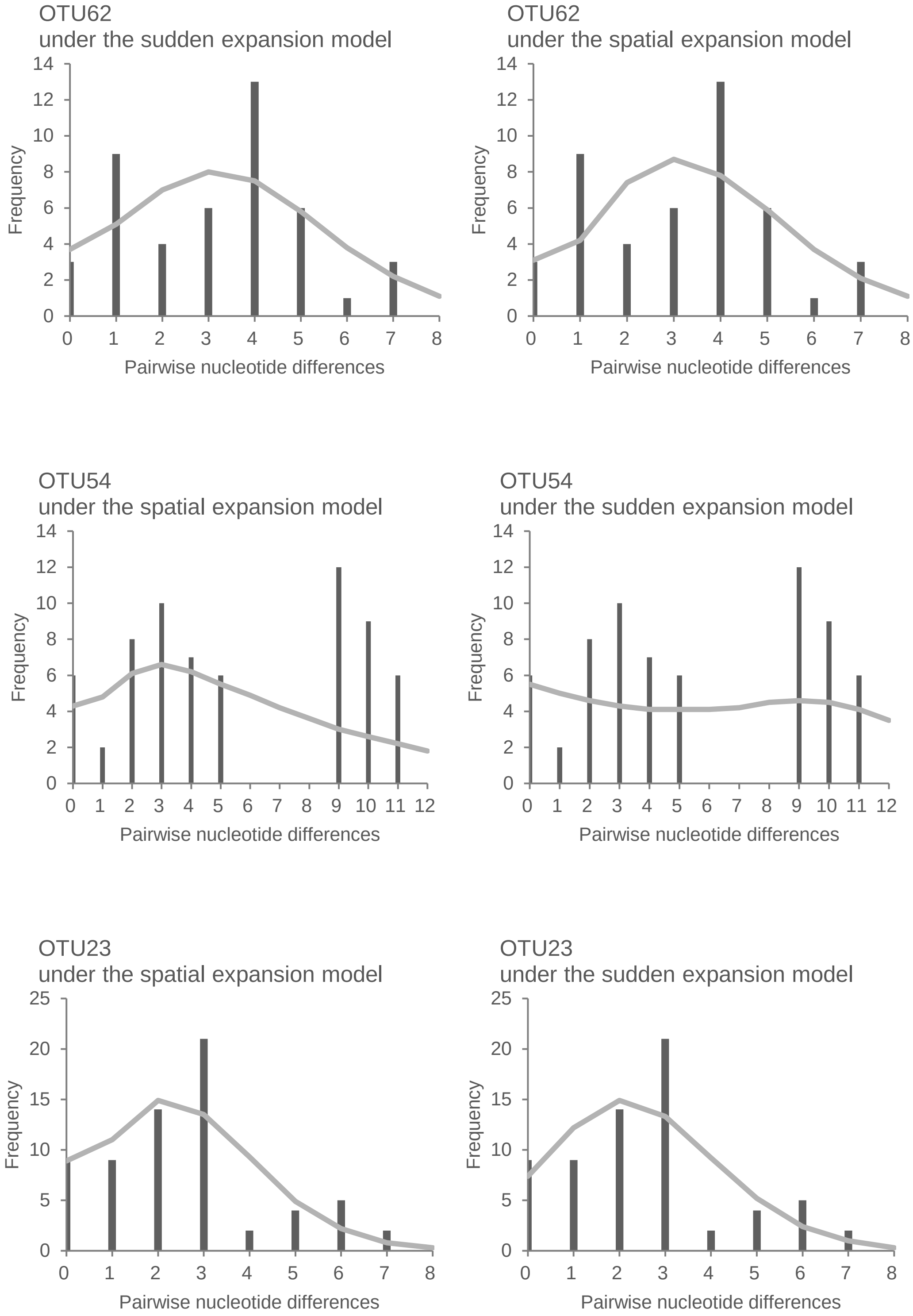
OTU62
under the sudden expansion model
OTU62
under the spatial expansion model
OTU54
under the spatial expansion model
OTU54
under the sudden expansion model
OTU23
under the spatial expansion model
OTU23
under the sudden expansion model
Frequency
Pairwise nucleotide differences

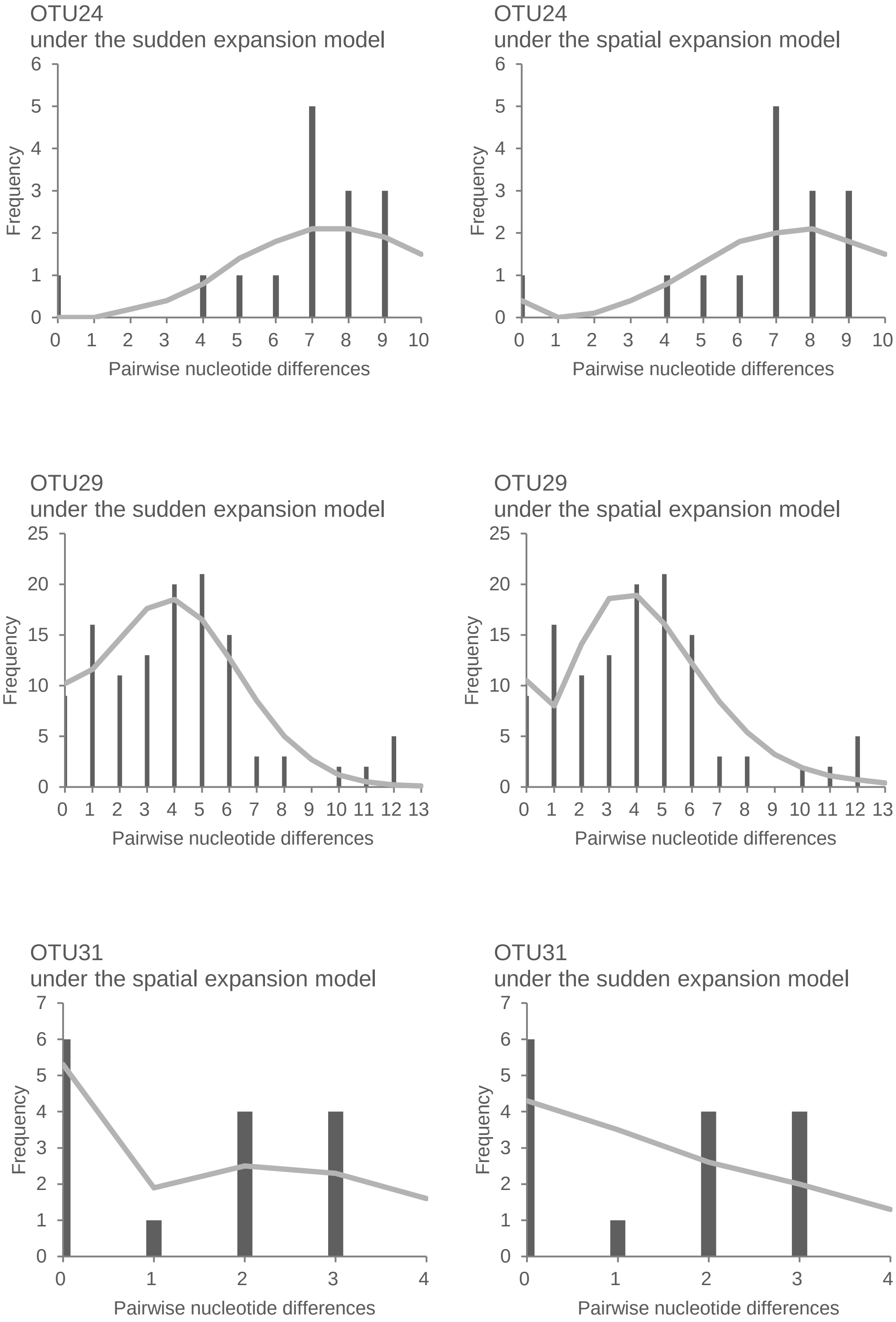
OTU24
under the sudden expansion model
Frequency
Pairwise nucleotide differences
OTU24
under the spatial expansion model
Frequency
Pairwise nucleotide differences
OTU29
under the sudden expansion model
Frequency
Pairwise nucleotide differences
OTU29
under the spatial expansion model
Frequency
Pairwise nucleotide differences
OTU31
under the spatial expansion model
Frequency
Pairwise nucleotide differences
OTU31
under the sudden expansion model
Frequency
Pairwise nucleotide differences

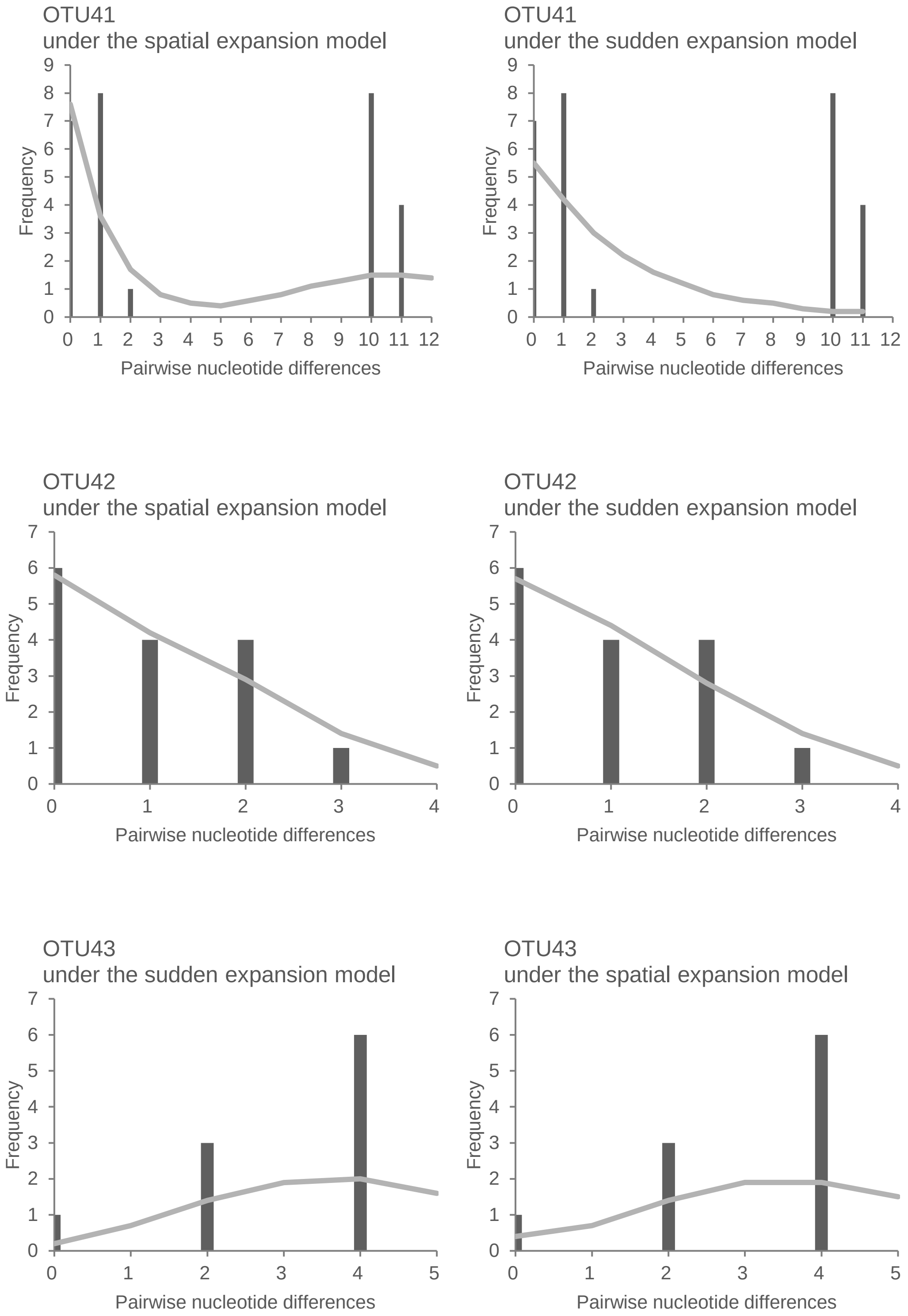
OTU41
under the spatial expansion model
Frequency
Pairwise nucleotide differences
OTU41
under the sudden expansion model
Frequency
Pairwise nucleotide differences
OTU42
under the spatial expansion model
Frequency
Pairwise nucleotide differences
OTU42
under the sudden expansion model
Frequency
Pairwise nucleotide differences
OTU43
under the sudden expansion model
Frequency
Pairwise nucleotide differences
OTU43
under the spatial expansion model
Frequency
Pairwise nucleotide differences

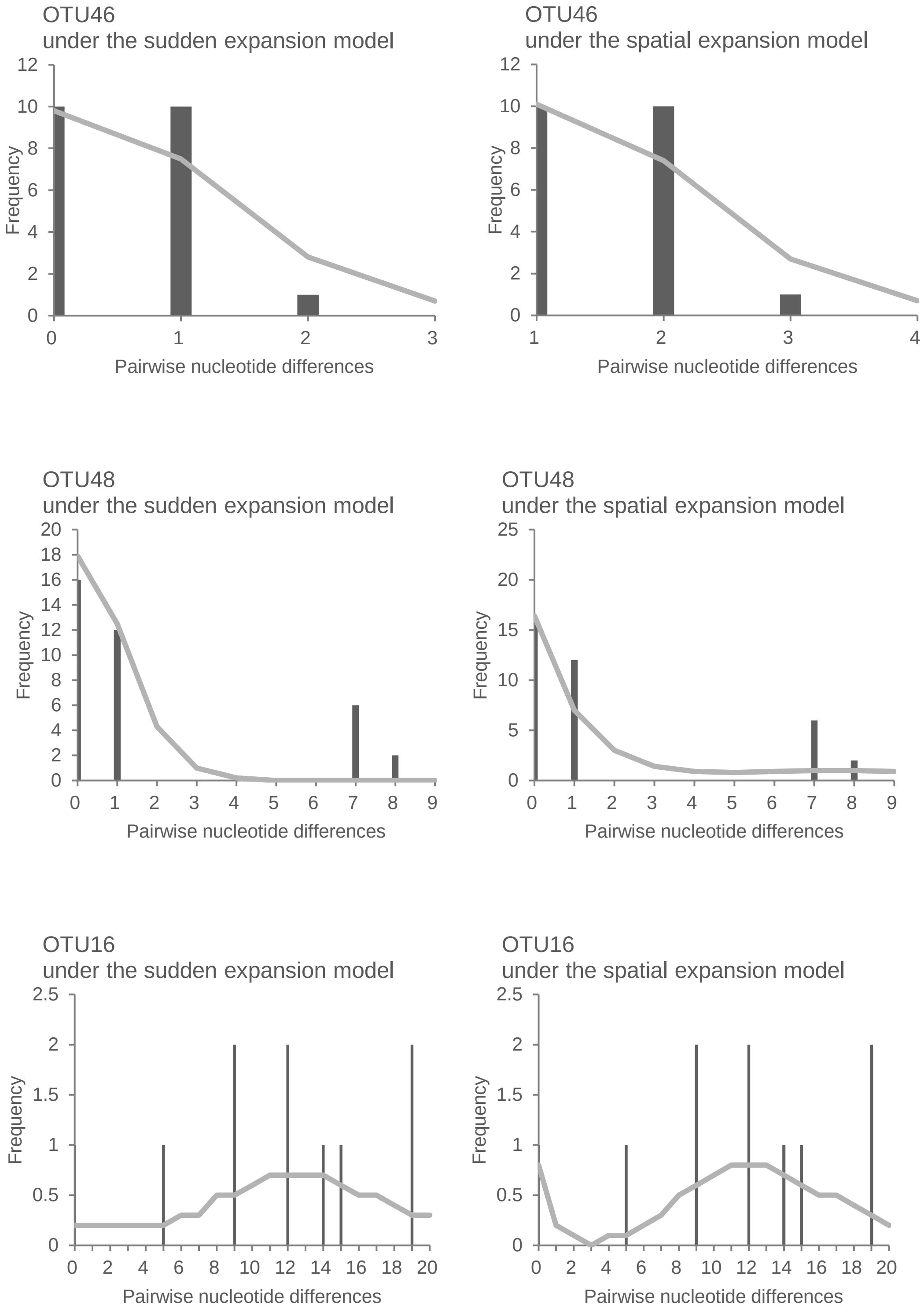
OTU46
under the sudden expansion model
Frequency
Pairwise nucleotide differences
OTU46
under the spatial expansion model
Frequency
Pairwise nucleotide differences
OTU48
under the sudden expansion model
Frequency
Pairwise nucleotide differences
OTU48
under the spatial expansion model
Frequency
Pairwise nucleotide differences
OTU16
under the sudden expansion model
Frequency
Pairwise nucleotide differences
OTU16
under the spatial expansion model
Frequency
Pairwise nucleotide differences

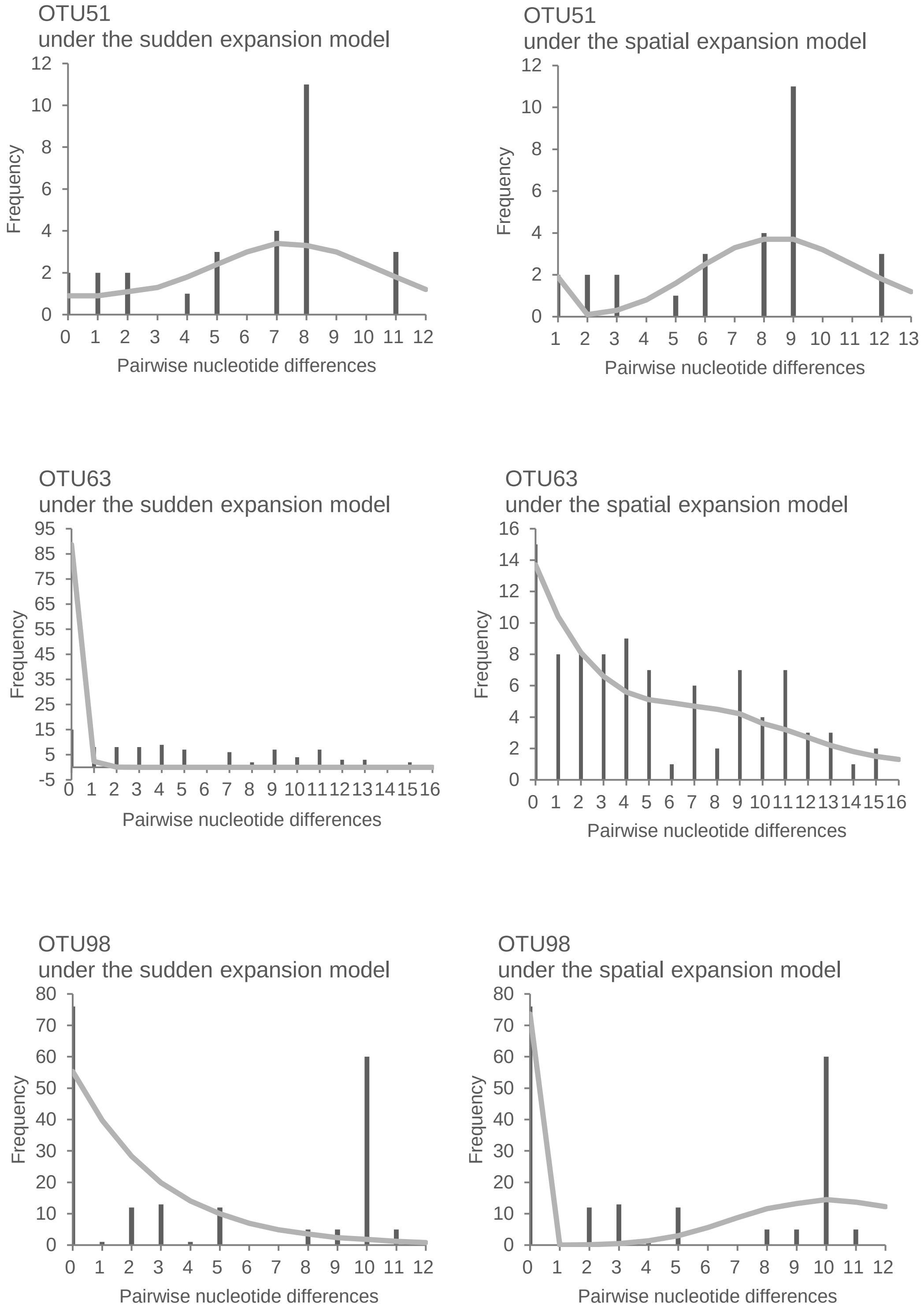
OTU51
under the sudden expansion model
Frequency
Pairwise nucleotide differences
OTU51
under the spatial expansion model
Frequency
Pairwise nucleotide differences
OTU63
under the sudden expansion model
Frequency
Pairwise nucleotide differences
OTU63
under the spatial expansion model
Frequency
Pairwise nucleotide differences
OTU98
under the sudden expansion model
Frequency
Pairwise nucleotide differences
OTU98
under the spatial expansion model
Frequency
Pairwise nucleotide differences

OTU59
under the sudden expansion model

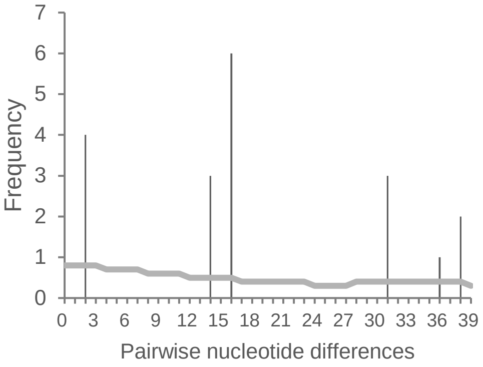

OTU59
under the spatial expansion model

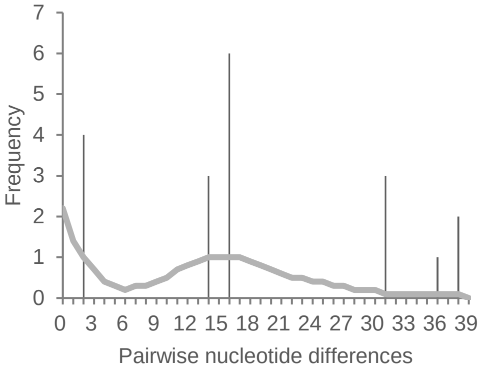

OTU72
under the sudden expansion model

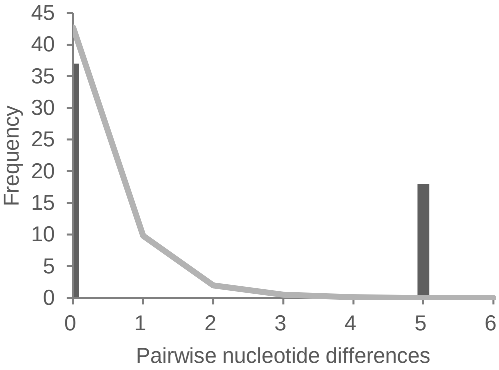

OTU72
under the spatial expansion model

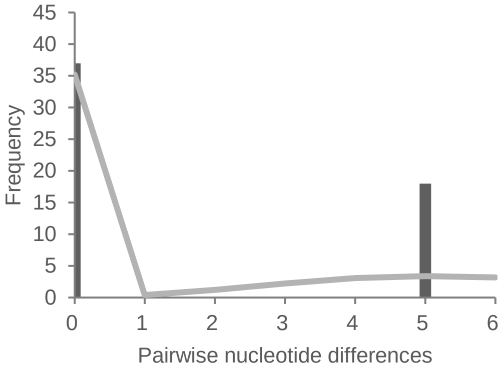

OTU4
under the sudden expansion model

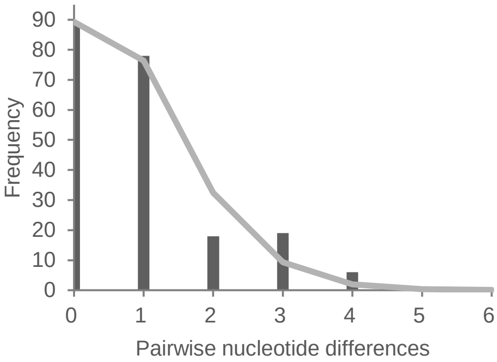

OTU4
under the spatial expansion model

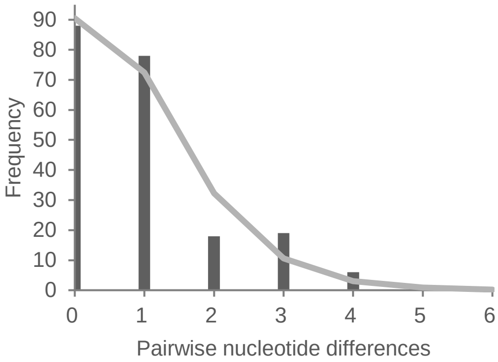

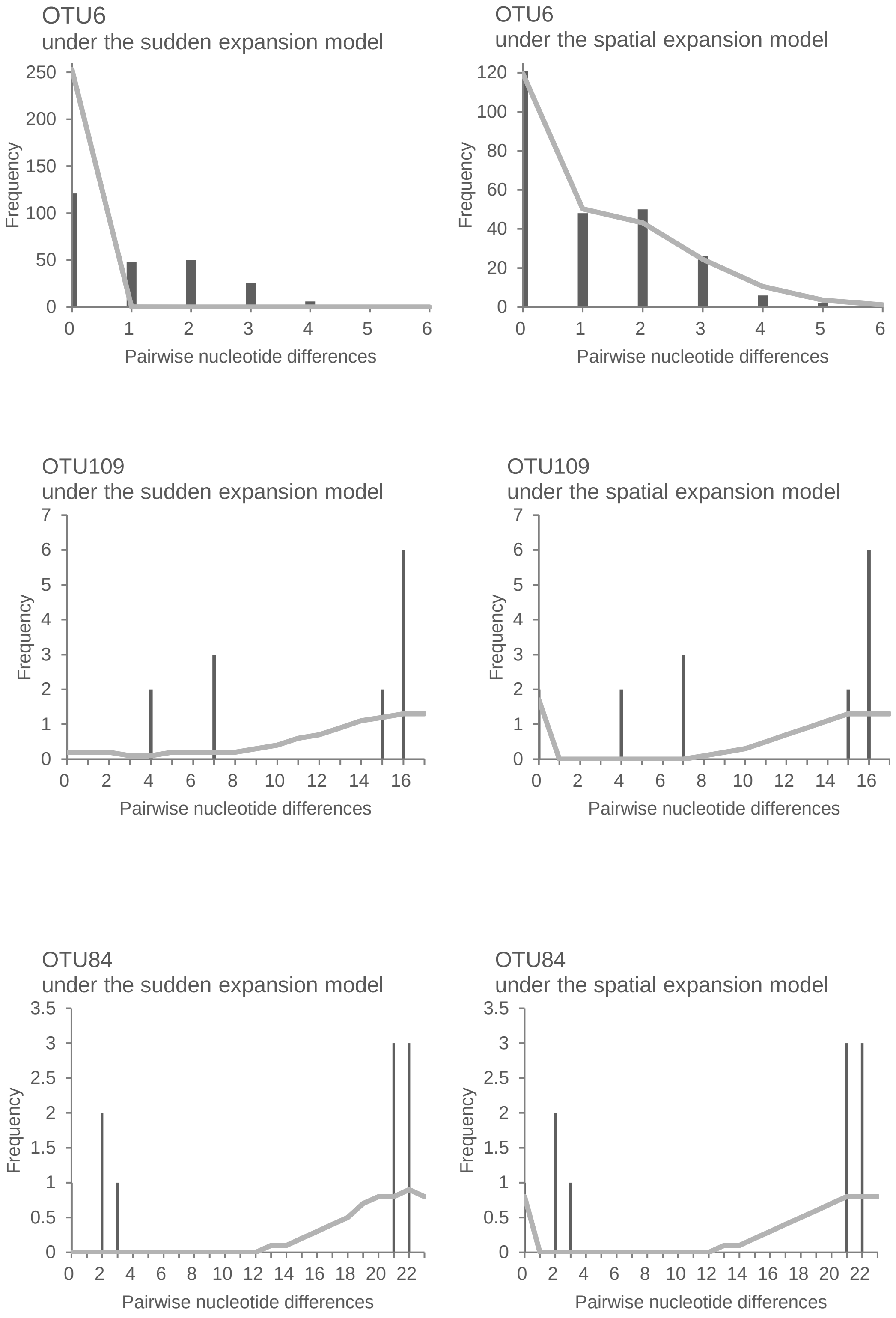
OTU6
under the sudden expansion model
Frequency
Pairwise nucleotide differences
OTU6
under the spatial expansion model
Frequency
Pairwise nucleotide differences
OTU109
under the sudden expansion model
Frequency
Pairwise nucleotide differences
OTU109
under the spatial expansion model
Frequency
Pairwise nucleotide differences
OTU84
under the sudden expansion model
Frequency
Pairwise nucleotide differences
OTU84
under the spatial expansion model
Frequency
Pairwise nucleotide differences

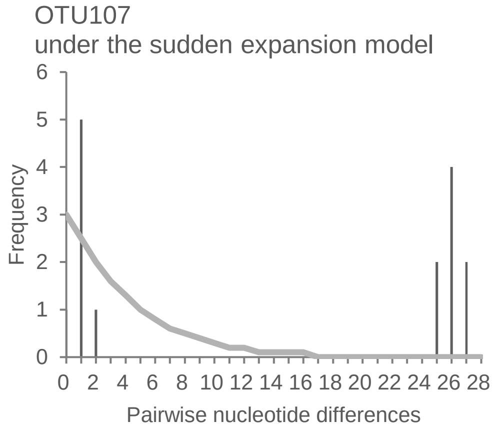
OTU107
under the sudden expansion model
Frequency
Pairwise nucleotide differences

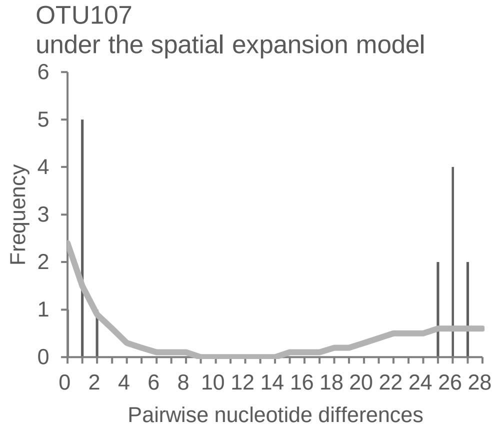
OTU107
under the spatial expansion model
Frequency
Pairwise nucleotide differences

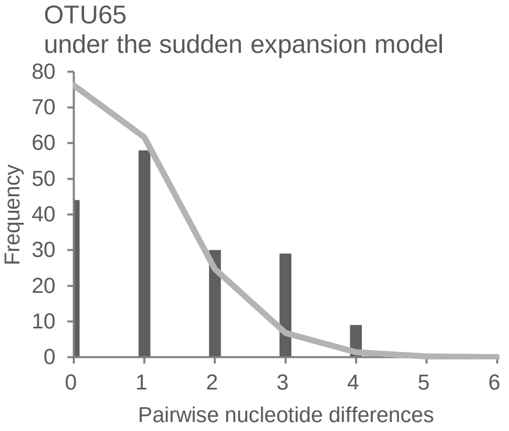
OTU65
under the sudden expansion model
Frequency
Pairwise nucleotide differences

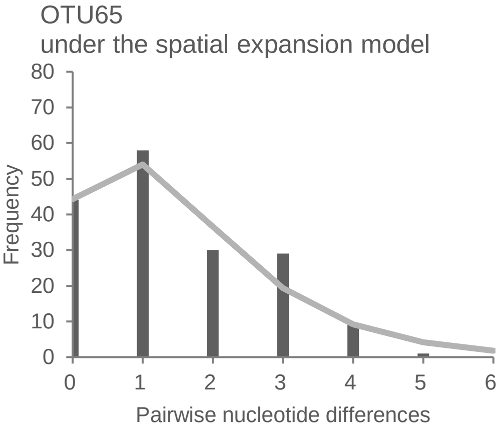
OTU65
under the spatial expansion model
Frequency
Pairwise nucleotide differences

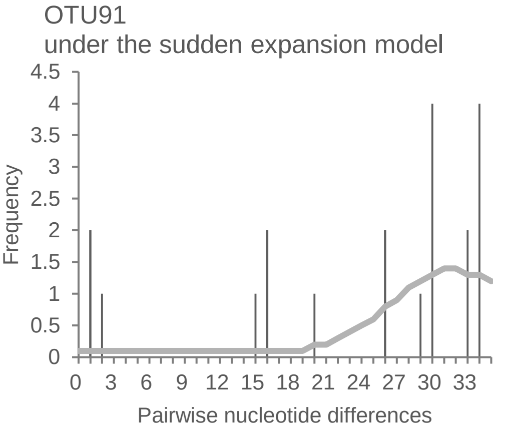
OTU91
under the sudden expansion model
Frequency
Pairwise nucleotide differences

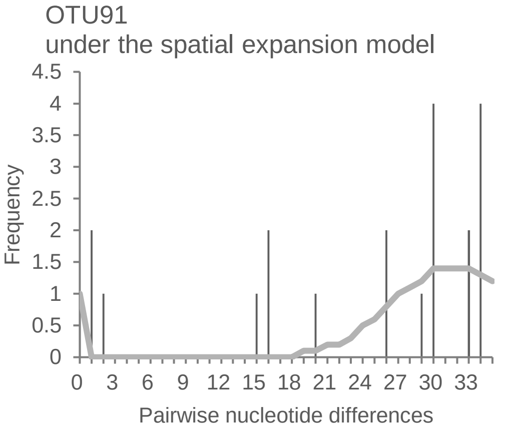
OTU91
under the spatial expansion model
Frequency
Pairwise nucleotide differences

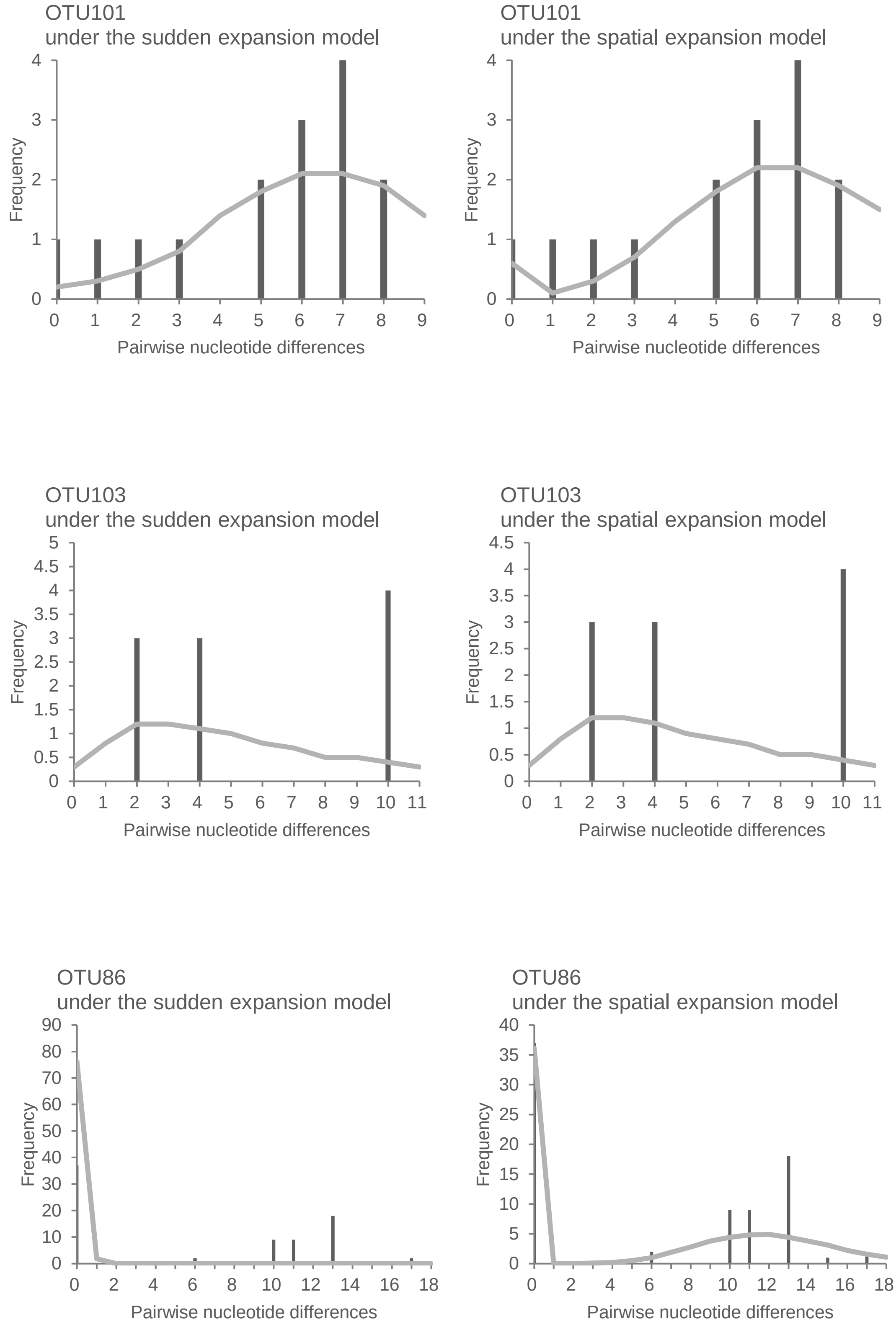
OTU101
under the sudden expansion model
Frequency
Pairwise nucleotide differences
OTU101
under the spatial expansion model
Frequency
Pairwise nucleotide differences
OTU103
under the sudden expansion model
Frequency
Pairwise nucleotide differences
OTU103
under the spatial expansion model
Frequency
Pairwise nucleotide differences
OTU86
under the sudden expansion model
Frequency
Pairwise nucleotide differences
OTU86
under the spatial expansion model
Frequency
Pairwise nucleotide differences

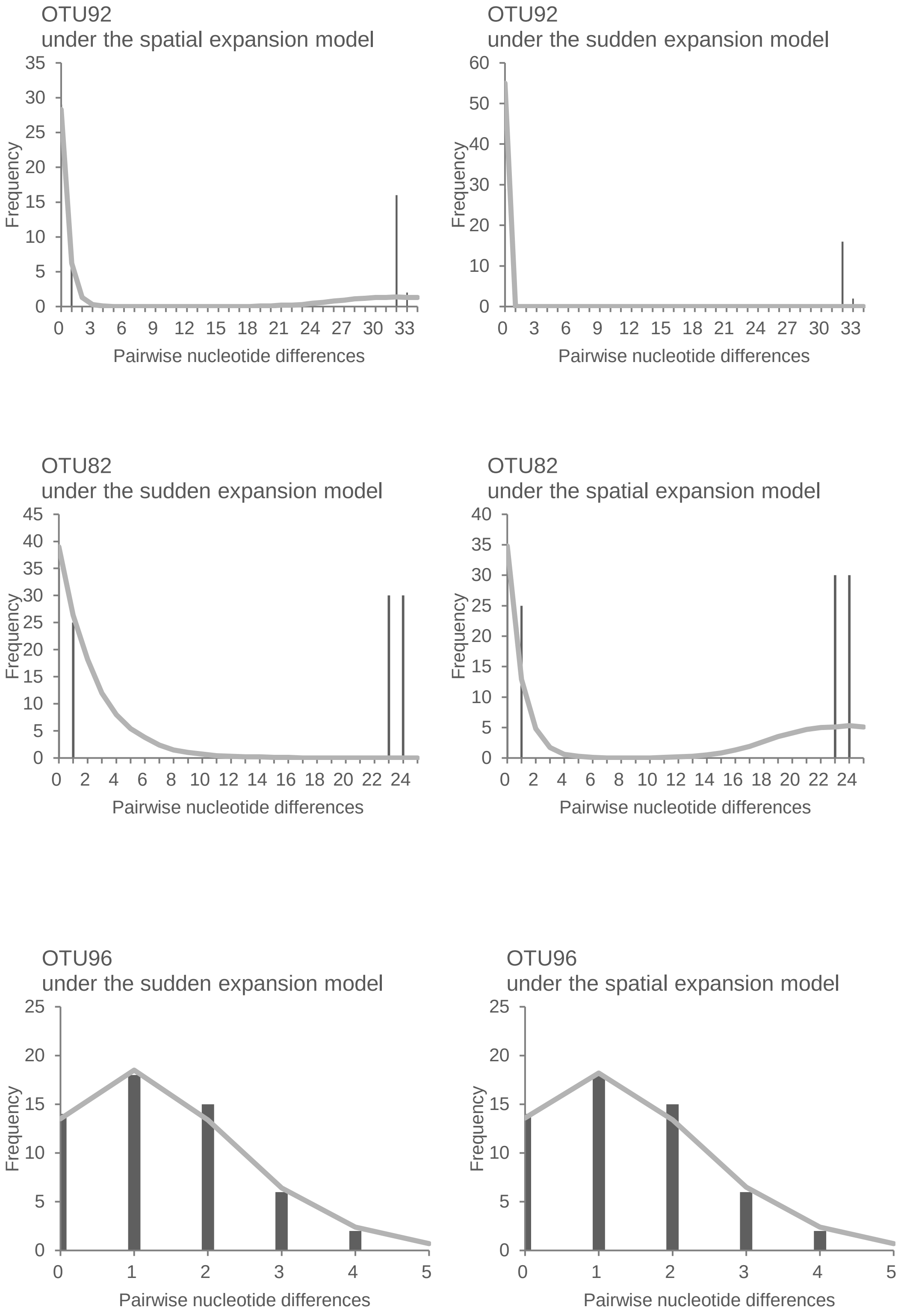
OTU92
under the spatial expansion model
Frequency
Pairwise nucleotide differences
OTU92
under the sudden expansion model
Frequency
Pairwise nucleotide differences
OTU82
under the sudden expansion model
Frequency
Pairwise nucleotide differences
OTU82
under the spatial expansion model
Frequency
Pairwise nucleotide differences
OTU96
under the sudden expansion model
Frequency
Pairwise nucleotide differences
OTU96
under the spatial expansion model
Frequency
Pairwise nucleotide differences

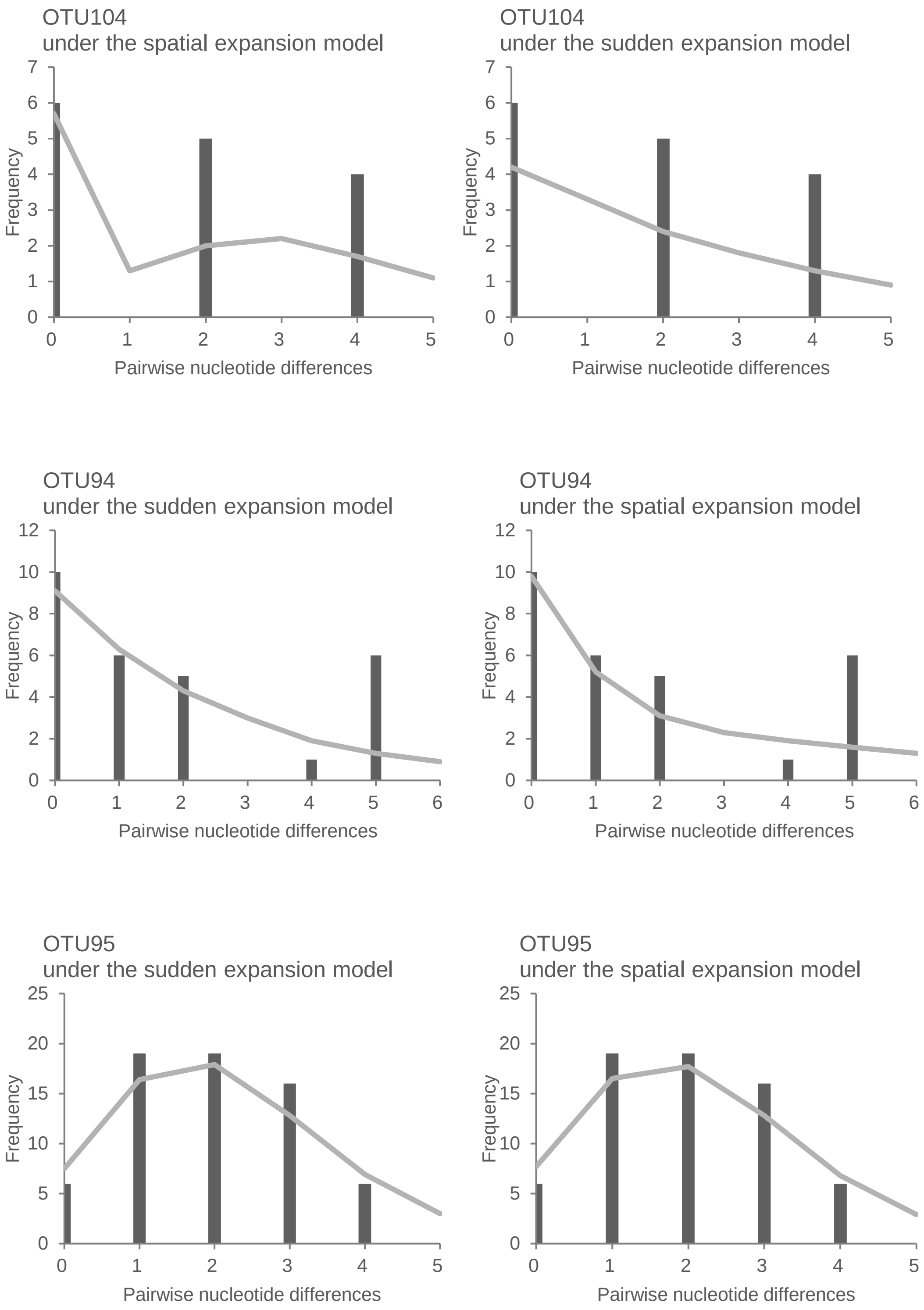
OTU104
under the spatial expansion model
OTU104
under the sudden expansion model
OTU94
under the sudden expansion model
OTU94
under the spatial expansion model
OTU95
under the sudden expansion model
OTU95
under the spatial expansion model
Frequency
Pairwise nucleotide differences

Fig. S3. Grouping from SNP calling based on mitochondrial DNA (mtDNA) tree clades.

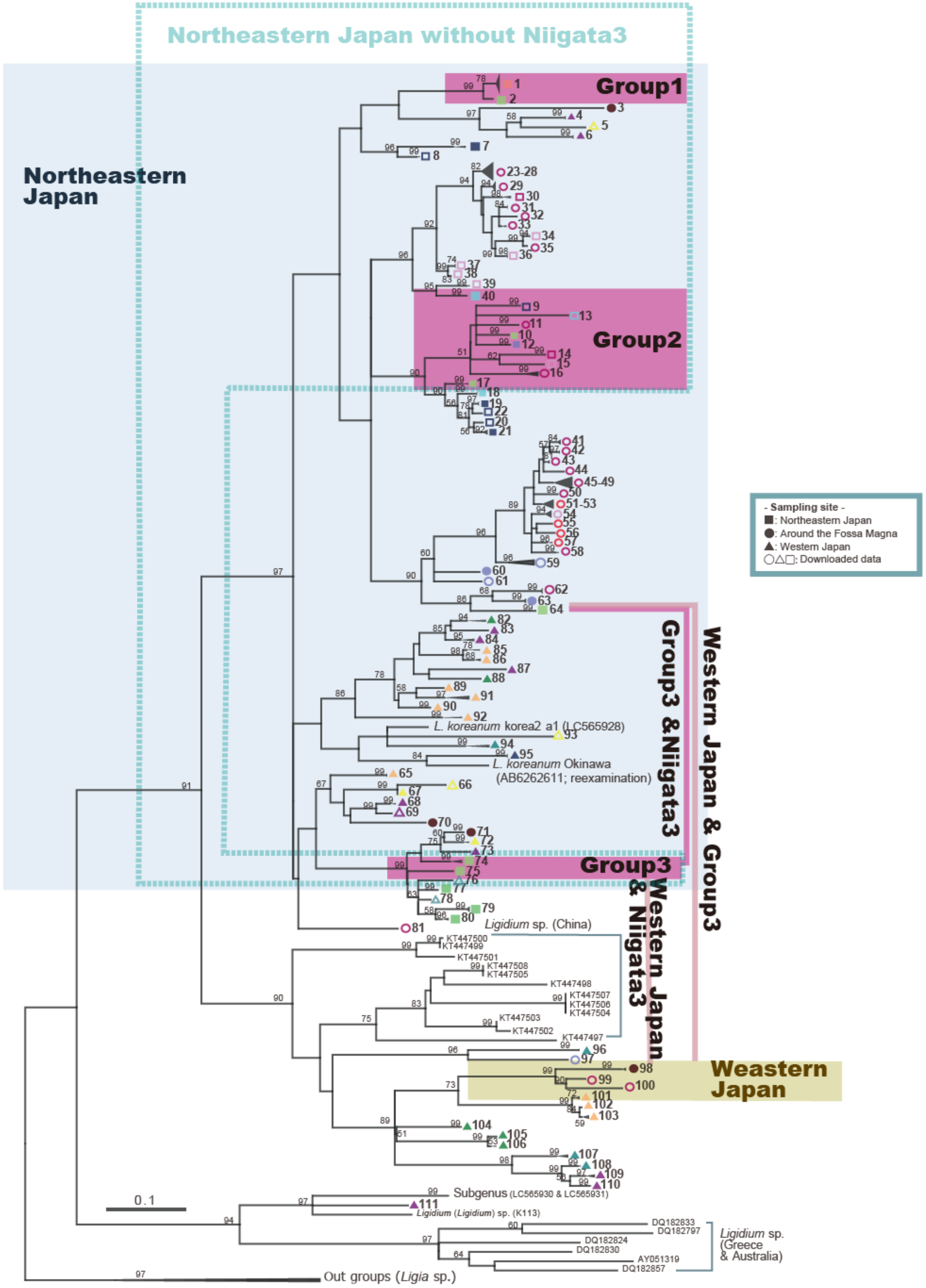

Supplementary Tables

Table S1 All results of the population genetic analyses.

(A) Results under the sudden expansion model.

MrDNA OTU ID	Sampling site ID	τ	Sum of Squared deviation	P (Sim. Ssd >= Obs. Ssd)	Harpending's Raggedness index	P (Sim. Rag. >= Obs. Rag.)	τ	Min	Max	Mean
7	50, 57	0.76	0.02	0.296	0.25	0.356	0.76	0.00	1.76	0.80
19	55, 57, Amigasamori-Forest, Aomori	2.59	0.06	0.058	0.18	0.076	2.59	0.88	4.15	2.63
21	48, 50, 54, 55	0.98	0.01	0.169	0.13	0.125	0.98	0.48	1.78	1.01
40	ST01	1.93	0.01	0.712	0.10	0.919	1.93	0.00	4.30	2.15
18	ST02	4.96	0.02	0.725	0.06	0.893	4.96	0.13	30.0	5.89
77	NS2	2.97	0.03	0.183	0.50	0.420	2.97	0.00	45.59	5.07
79	NS3	4.74	0.08	0.050	0.19	0.051	4.74	0.43	8.65	4.62
80	NS4	3.00	0.08	0.062	0.47	0.535	3.00	0.41	3.13	2.16
62	Gunma	4.06	0.03	0.328	0.10	0.357	4.06	1.19	7.16	3.91
34	Tochigi/Ibaraki	2.79	0.05	0.401	0.20	0.610	2.79	0.00	5.3	3.0
36	Tsukuba-shi, Ibaraki	3.00	0.15	0.034	0.67	0.600	3.00	0.42	3.16	2.10
37	Kashima-shi, Ibaraki	1.74	0.01	0.659	0.13	0.645	1.74	0.00	3.6	1.9
54	Chiba/Ibaraki	11.2	0.05	0.237	0.07	0.558	11.18	0.02	100.18	15.45

23	Boso peninsula, Chiba	2.86	0.03	0.264	0.10	0.297	2.86	0.73	5.19	2.77
24	Boso peninsula, Chiba	8.07	0.06	0.234	0.14	0.558	8.07	4.02	11.5	8.1
29	Boso peninsula, Chiba	4.85	0.01	0.670	0.02	0.850	4.85	1.39	8.11	4.60
31	Boso peninsula, Chiba	3.27	0.06	0.292	0.22	0.557	3.27	0.00	52.93	4.69
41	Boso peninsula, Chiba	11.2	0.14	0.201	0.19	0.460	11.19	0.07	94.19	19.70
42	Boso peninsula, Chiba	1.73	0.01	0.746	0.06	0.988	1.73	0.00	4.0	1.7
43	Boso peninsula, Chiba	4.03	0.24	0.017	0.91	0.103	4.03	1.17	7.6	5.8
46	Boso peninsula, Chiba	0.73	0.02	0.527	0.19	0.761	0.73	0.00	2.16	0.93
48	Boso peninsula, Chiba	0.67	0.05	0.165	0.17	0.782	0.67	0.00	1.7	0.7
16	Chiba/Saitama/Tokyo	11.0	0.11	0.268	0.29	0.314	11.04	4.41	86.04	15.27
51	Kawasaki-shi, Kanagawa	8.28	0.12	0.037	0.28	0.052	8.28	3.16	12.11	7.92
63	NK	0.00	0.72	0.000	0.03	1.000	0.00	0.00	0.5	0.0

98	SK01, SH01, Hamamatsu (Shizuoka)	10.5	0.16	0.084	0.34	0.090	10.49	0.00	93.49	18.13
59	Yamanashi/Shizuoka	38.07	0.15	0.051	0.35	0.038	38.07	0.37	155.1	45.6
72	O1	3.00	0.15	0.035	0.67	0.498	3.00	0.41	3.00	2.16
4	Y23, T66, S58	0.87	0.01	0.427	0.09	0.566	0.87	0.00	1.77	0.89
6	Shi61, Shi66, Shi122, Shi124, Tr80, Y21, Hrs19, Y41, Oym15	0.00	0.36	0.000	0.10	1.000	0.00	0.00	0.00	0.00
109	Shi124, Shi05, Shi115, Y23, Y25, Sugiura (Shimane)	23.3	0.17	0.017	0.25	0.634	23.3	14.5	474	47.5
84	Y23, Y25, Y41, Y45	23.3	0.17	0.017	0.25	0.634	23.3	14.5	474	47.5
107	Oki93, Oki95, Oki98	1.84	0.19	0.184	0.22	0.475	1.84	0.00	86.8	6.81
65	T11, S58	0.78	0.06	0.042	0.05	0.909	0.78	0.03	1.72	0.83
91	E6, E7, E8	33.8	0.08	0.047	0.16	0.363	33.8	22.4	40.5	32.2
101	E8	7.16	0.03	0.475	0.07	0.886	7.16	2.83	11.0	7.29

103	E13, E14, E15, E19, Kumakogen-cho (Ehime)	0.63	0.23	0.009	0.14	0.802	0.63	0.00	1.6	0.67
86	E15, E20	0.00	0.36	0.000	0.36	0.954	0.00	0.00	0.49	0.03
92	K11	0.00	0.33	0.000	0.32	1.000	0.00	0.00	0.00	0.00
82	F03, SG01	0.87	0.04	0.162	0.32	0.131	0.87	0.00	1.8	0.97
96	FI01	1.47	0.00	0.925	0.04	0.933	1.47	0.00	2.95	1.51
104	F03, F161, F147	4.35	0.13	0.130	0.52	0.194	4.35	0.01	76	6.5
94	TS03, TS28	6.14	0.04	0.572	0.13	0.748	6.14	0.00	59	9.78
95	OK11	2.15	0.01	0.707	0.07	0.530	2.15	0.55	3.65	2.18

(B) Results under the spatial expansion model.

MtDNA OTU ID	Sampling site ID	τ	Sum of Squared deviation	P (Sim. Ssd >= Obs. Ssd)	Harpending's Raggedness index	P(Sim. Rag. >= Obs. Rag.)	τ	Min	Max	Mean
7	50, 57	0.76	0.02	0.135	0.25	0.342	0.76	0.00	85.8	5.90
19	55, 57, Amigasamori-Forest, Aomori	2.59	0.06	0.035	0.18	0.071	2.59	0.87	4.02	2.51

21	48, 50, 54, 55	0.99	0.01	0.094	0.13	0.127	0.99	0.31	1.65	1.02
40	ST01	1.41	0.01	0.729	0.10	0.926	1.41	0.00	3.94	2.52
18	ST02	2.74	0.02	0.699	0.06	0.919	2.74	0.31	12.6	5.62
77	NS2	0.14	0.00	0.407	0.50	0.692	0.14	0.00	2.73	2.07
79	NS3	3.78	0.08	0.088	0.19	0.262	3.78	0.83	7.11	4.28
80	NS4	13.8	0.03	0.583	0.47	0.817	13.8	0.00	86.1	17.4
62	Gunma	3.0	0.04	0.247	0.10	0.363	3.0	0.98	6.2	3.5
34	Tochigi/Ibaraki	2.7	0.04	0.405	0.20	0.644	2.7	N.A.	N.A.	N.A.
36	Tsukuba-shi, Ibaraki	21.1	0.09	0.294	0.67	0.874	21.1	0.00	168.0	22.1
37	Kashima-shi, Ibaraki	1.74	0.01	0.645	0.13	0.645	1.74	0.42	3.57	2.25
54	Chiba/Ibaraki	1.76	0.05	0.086	0.07	0.472	1.76	0.30	81	8.25
23	Boso peninsula, Chiba	2.7	0.03	0.346	0.10	0.385	2.73	0.65	5	2.6
24	Boso peninsula, Chiba	8.05	0.06	0.288	0.14	0.616	8.05	3.64	12	8.2
29	Boso peninsula, Chiba	3.15	0.01	0.592	0.02	0.889	3.15	0.51	9.90	4.17
31	Boso peninsula, Chiba	2.71	0.03	0.592	0.22	0.728	2.71	0.00	86	8.7
41	Boso peninsula, Chiba	10.0	0.10	0.174	0.19	0.676	10.0	0.00	171	18.4

42	Boso peninsula, Chiba	1.45	0.01	0.828	0.06	0.992	1.4	0.0	4	2.5
43	Boso peninsula, Chiba	4.03	0.24	0.012	0.91	0.104	4.0	1.0	64	7.7
46	Boso peninsula, Chiba	0.73	0.02	0.325	0.19	0.772	0.73	0.00	2	2.13
48	Boso peninsula, Chiba	7.57	0.05	0.315	0.17	0.828	7.57	0.00	118	11.5
16	Chiba/Saitama/Tokyo	10.2	0.10	0.399	0.29	0.435	10.18	4.85	21	13.4
51	Kawasaki-shi, Kanagawa	7.76	0.12	0.076	0.28	0.129	7.76	3.87	11	7.7
63	NK	9.46	0.02	0.826	0.03	0.958	9.46	0.34	88	10.3
98	SK01, SH01, Hamamatsu (Shizuoka)	10.8	0.08	0.261	0.34	0.666	10.8	0.00	86	14.9
59	Yamanashi/Shizuoka	12.7	0.14	0.091	0.35	0.085	12.7	1.32	35	16.5
72	01	5.63	0.08	0.257	0.67	0.756	5.63	0.00	121	18.4
4	Y23, T66, S58	0.58	0.01	0.376	0.09	0.672	0.58	0.21	2.54	1.10
6	Shi61, Shi66, Shi122, Shi124, Tr80, Y21,	1.72	0.00	0.875	0.10	0.835	1.72	0.00	4	1.9

	Hrs19, Y41, Oym15									
109	Shi124, Shi05, Shi115, Y23, Y25, Sugiura (Shimane)	17.2	0.17	0.032	0.38	0.495	17.2	0.00	171	24.6
84	Y23, Y25, Y41, Y45	23.17	0.16	0.055	0.25	0.753	23.2	11.0	171	32.2
107	Oki93, Oki95, Oki98	24.6	0.14	0.127	0.22	0.633	24.6	0.00	193	29.4
65	T11, S58	0.79	0.01	0.564	0.05	0.790	0.79	0.36	4	2.0
91	E6, E7, E8	32.8	0.08	0.122	0.16	0.569	32.8	23.5	41	32.5
101	E8	7.03	0.03	0.543	0.07	0.916	7.03	1.94	11	7.1
103	E13, E14, E15, E19, Kumakogen-cho (Ehime)	0.63	0.05	0.277	0.14	0.452	0.63	0.31	9	3.3
86	E15, E20	12.1	0.05	0.723	0.36	0.685	12.15	0.00	19	12.4
92	K11	33.0	0.08	0.316	0.32	0.618	33.02	0.00	192	33.7
82	F03, SG01	0.87	0.04	0.078	0.32	0.143	0.87	0.13	85	6.1
96	FI01	1.44	0.00	0.922	0.04	0.925	1.44	0.43	3	1.6
104	F03, F161, F147	3.19	0.09	0.244	0.52	0.473	3.19	0.00	86	7.2
94	TS03, TS28	4.26	0.04	0.447	0.13	0.819	4.26	0.00	114	10.7
95	OK11	2.15	0.01	0.659	0.07	0.514	2.15	0.71	3.49	2.13

We calculated these values using Arlequin 3.5.1.2; the sum of square deviations (SSD) between the observed and the expected mismatch as a test statistic, the estimated expansion parameter τ (with 95% confidence intervals). To calculate the degree of deviation from neutral evolution and determine the presence or absence of natural selection, we used Tajima's D [19] and Fu's Fs [20] neutrality tests. Negative Tajima's D and Fu's Fs statistics indicate population expansion, zero values indicate a constant size, and positive values indicate a decreased population. Statistics were computed from 10,000 bootstrap pseudo-replicates within 95% confidence intervals (*$P < 0.05$, **$P < 0.01$). (A) Results under the sudden expansion model. (B) Results under the spatial expansion model. Table S1 lists the sampling site IDs corresponding with the Site IDs and underlined text indicates the sample sites in Yoshino and Kubota (2022) [14].

Table S2 List of Japanese *Ligidium* individuals in genome-wide analyses.

Sampling site ID	n	MtDNA-ID (n)	Groups of SNPs calling	
			stacks	ipyrad
South Hokkaido (44)	6	1	Northeastern Japan	Northeastern Japan
East Hokkaido (65, 66)	6	1	Northeastern Japan	Northeastern Japan
Central Hokkaido (9, 10, 25, 27)	4	1	Northeastern Japan	Northeastern Japan
Aomori (48)	6	1	Northeastern Japan	Northeastern Japan
Niigata				
inland (32)	16	1 (5), 10 (5), 74 (6)	Northeastern Japan	Northeastern Japan
Echigo plain (33)	11	1 (5), 17 (6)	Northeastern Japan	Northeastern Japan
Mt. Kakuda (31)	10	64 (5), 74 (5)	Northeastern Japan	Northeastern Japan
Coastline (30)	5	75	Northeastern Japan	Northeastern Japan
Sendai (ST01)	4	40	Northeastern Japan	Northeastern Japan
Shizuoka (SK01)	5	3	Western Japan	Western Japan
Ehime (E8)	5	101	Western Japan	Western Japan
Ligia sp. from Niigata coastline	4	Outgroup		

Sampling location, sample size (n), affiliated mtDNA-OTU ID, the grouping in SNP calling.

Table S3 Results of SNPs calling for each group.

(A) SNPs calling by stacks program, denovo_map.pl.

Group name	MtDNA-OTU	Sample size before SNPs sampling	Sample size after all filtering	denovo_map.pl				Number of variable loci passing filter of Tassel	Number of variable SNP passing filter of Tassel
				Number of populations (geographic range of sampling)	Number of reads used	Mean. depth of coverage	Number of loci		
Group1	1	33	28	7	66912118	37.6	485089	202	3472
Group2	40, 10, 17	11	11	3	15911214	39.9	263410	5154	28876
Group3	74, 75	16	8	3	39214555	40	263398	16324	82911
Western Japan	3, 101	10	10	2	33104623	42.8	287617	272	1661
North-eastern Japan	1, 40, 10, 17, 64, 74, 75	68	67	10	138087186	35.3	917118	78	961
Western Japan & group3	74, 75, 3, 101	26	25	5	72265881	35.9	547093	20	88
Northeastern Japan without group3	1, 40, 10, 17, 64	49	48	7	88956755	35.7	644108	85	926

Group3 & Niigata3	64, 74, 75	21	20	4	46121138	33	348137	130	993
Western Japan & Niigata3	64, 3, 101	15	13	3	40008784	40.8	377920	14	67

For the more subdivided groups (group 2, 3, and western group: Table S3A; Fig. S2), we raised the threshold for shared to 80%.

(B) **SNPs calling by ipyrad.**

				ipyrad					
**Group name**	**MtDNA-OTU**	**Sample size before SNPs sampling**	**Sample size after all filtering**	**Number of populations (geographic range of sampling)**	**Number of prefiltered loci**	**Mean. depth of coverage**	**Number of loci passing filter of ipyrad**	**Number of loci passing filter of Tassel**	**Number of variable SNP passing filter of Tassel**
Northeastern Japan	1, 9, 10, 17, 64, 74, 75	68	65	10	179738	126	135	134	3319
Northeastern and Western	1, 64, 74, 75, 9, 101, 3	83	33	5	179313	18	25	24	644
Western Japan	101, 3	10	10	2	179313	710	750	747	12866

Table S4 List of following data: accession numbers of all mitochondrial haplotypes including outgroups, RAD-seq data ID, morphology, sampling locations.

Mitochondrial Haplotype	Accession number of mtDNA	MtDNA-OTU ID	Genome-wide analyses ID	Morphology	Sampling site (ID)	Latitude	Longitude
NJEH (6617)	LC711207	1	112		East Hokkaido (66)	44°03'32.723"N	144°59'46.751"E
NJSH1 (s21)	LC711254	1			South Hokkaido (65)	42°08'44.66"N	140°02'06.28"E
NJA1	LC711204	1	111		Aomori (57)	41°02'19.83"N	140°25'41.67"E
NJA2	LC711205	1	106		Aomori (48)	41°31'39.88"N	140°56'13.98"E
NJA3	LC711206	1	107, 108, 109, 110	*L. japonicum* (n=2; 4811, 4817)	Aomori (48)	41°31'39.88"N	140°56'13.98"E
A1	LC711144	7		*L. japonicum* (n=1; 5014)	Aomori (50)	41°26'53.80"N	141°06'52.66"E
A2a	LC711145	7		*L. japonicum* (n=3; 5705, 5711, 5718)	Aomori (57)	41°02'19.83"N	140°25'41.67"E
A2b	LC711146	7		*L. japonicum* (n=1; 5719)	Aomori (57)	41°02'19.83"N	140°25'41.67"E
A3a	LC711147	21			Aomori (48)	41°31'39.88"N	140°56'13.98"E
				L. japonicum (n=2; 5005, 5012)	Aomori (50)	41°26'53.80"N	141°06'52.66"E

			L. japonicum (n=2; 5402, 5412)	Aomori (54)	41°09'22.21"N	141°17'29.87"E
				Aomori (55)	40°55'38.62"N	140°59'51.41"E
A3b	LC711148	21		Aomori (50)	41°26'53.80"N	141°06'52.66"E
A3c	LC711149	21		Aomori (50)	41°26'53.80"N	141°06'52.66"E
A3d	LC711150	21		Aomori (54)	41°09'22.21"N	141°17'29.87"E
A3e	LC711151	21	*L. japonicum* (n=6; 5504, 5508, 5515, 5516, 5517, 5520)	Aomori (55)	40°55'38.62"N	140°59'51.41"E
A3f	LC711152	21	*L. japonicum* (n=1; 5506)	Aomori (55)	40°55'38.62"N	140°59'51.41"E
A3g	LC711153	21		Aomori (50)	41°26'53.80"N	141°06'52.66"E
A3h	LC711154	21		Aomori (48)	41°31'39.88"N	140°56'13.98"E
A4a	LC711155	19		Aomori (48)	41°31'39.88"N	140°56'13.98"E
A4b	LC711156	19	*L. japonicum* (n=1; 5708)	Aomori (57)	41°02'19.83"N	140°25'41.67"E
A4c	LC711157	19		Aomori (55)	40°55'38.62"N	140°59'51.41"E
A4d	LC711158	19		Aomori (55)	40°55'38.62"N	140°59'51.41"E
A4e	LC711159	19	*L. japonicum* (n=1; 5519)	Aomori (55)	40°55'38.62"N	140°59'51.41"E
A4f	LC711160	19		Aomori (55)	40°55'38.62"N	140°59'51.41"E
A5a	LC711161	9		Aomori (54)	41°09'22.21"N	141°17'29.87"E

A5b	LC711162	9		*L. japonicum* (n=1; 5707)	Aomori (57)	41°02'19.83"N	140°25'41.67"E
ST0101	LC711275	9			Sendai (ST01)	38°14'21.012"N	140°48'30.995"E
ST0102	LC711276	40	167		Sendai (ST01)	38°14'21.012"N	140°48'30.995"E
ST0108	LC711277	40	169		Sendai (ST01)	38°14'21.012"N	140°48'30.995"E
ST011	LC711278	40	170	*L. japonicum* (n=1; ST0103)	Sendai (ST01)	38°14'21.012"N	140°48'30.995"E
ST0202	LC711279	18			Sendai (ST02)	38°14'03.983"N	140°48'15.011"E
ST0204	LC711280	18			Sendai (ST02)	38°14'03.983"N	140°48'15.011"E
ST0206	LC711281	18		*L. japonicum* (n=1; ST0206)	Sendai (ST02)	38°14'03.983"N	140°48'15.011"E
ST0208	LC711282	18		*L. japonicum* (n=1; ST0208)	Sendai (ST02)	38°14'03.983"N	140°48'15.011"E
ST021	LC711283	18		*L. japonicum* (n=2; ST0205, ST0207)	Sendai (ST02)	38°14'03.983"N	140°48'15.011"E
NS021	LC711220	77		*L.* sp. NIIGATA1 (n=1; NS021)	Sado (NS02)	37°51'29.073"N	138°19'56.082"E
NS0211	LC711221	77		*L.* sp. NIIGATA1 (n=1; NS021)	Sado (NS02)	37°51'29.073"N	138°19'56.082"E
NS0213	LC711222	77			Sado (NS02)	37°51'29.073"N	138°19'56.082"E
NS0301	LC711223	79		*L.* sp. NIIGATA1 (n=1; NS0301)	Sado (NS03)	38°03'40.021"N	138°21'24.059"E

NS0306	LC711224	79		Sado (NS03)	38°03'40.021"N	138°21'24.059"E
NS0309	LC711225	79		Sado (NS03)	38°03'40.021"N	138°21'24.059"E
NS031	LC711226	79	*L.* sp. NIIGATA1 (n=2; NS0302, NS0311)	Sado (NS03)	38°03'40.021"N	138°21'24.059"E
NS0314	LC711227	79		Sado (NS03)	38°03'40.021"N	138°21'24.059"E
NS0316	LC711228	79	*L.* sp. NIIGATA1 (n=1; NS0316)	Sado (NS03)	38°03'40.021"N	138°21'24.059"E
NS0317	LC711229	79	*L.* sp. NIIGATA1 (n=1; NS0317)	Sado (NS03)	38°03'40.021"N	138°21'24.059"E
NS0318	LC711230	79		Sado (NS03)	38°03'40.021"N	138°21'24.059"E
NS032	LC711231	79	*L.* sp. NIIGATA1 (n=3; NS0304, NS0313, NS0315)	Sado (NS03)	38°03'40.021"N	138°21'24.059"E
NS0408	LC711232	80	*L.* sp. NIIGATA1 (n=1; NS0408)	Sado (NS04)	38°19'18.092"N	138°30'50.078"E
NS041	LC711233	80	*L.* sp. NIIGATA1 (n=10; NS0401, NS0405, NS0409, NS0410, NS0415, NS0417, NS0418, NS0420, NS0422, NS0423)	Sado (NS04)	38°19'18.092"N	138°30'50.078"E

NS0413	LC711235	80			Sado (NS04)	38°19'18.092"N	138°30'50.078"E
NS042	LC711234	80			Sado (NS04)	38°19'18.092"N	138°30'50.078"E
NK0101	LC711208	63			Nagano (NK)	36°49'31.943"N	138°09'12.600"E
NK0102	LC711209	63			Nagano (NK)	36°49'31.943"N	138°09'12.600"E
NK0103	LC711210	63			Nagano (NK)	36°49'31.943"N	138°09'12.600"E
NK0104	LC711211	63			Nagano (NK)	36°49'31.943"N	138°09'12.600"E
NK0106	LC711212	63			Nagano (NK)	36°49'31.943"N	138°09'12.600"E
NK0109	LC711213	12			Nagano (NK)	36°49'31.943"N	138°09'12.600"E
NK011	LC711214	63		*L. japonicum* (n=5; NK0105, NK0111, NK0115, NK0119, NK0120)	Nagano (NK)	36°49'31.943"N	138°09'12.600"E
NK0112	LC711215	60			Nagano (NK)	36°49'31.943"N	138°09'12.600"E
NK0114	LC711216	63			Nagano (NK)	36°49'31.943"N	138°09'12.600"E
NK0118	LC711217	12			Nagano (NK)	36°49'31.943"N	138°09'12.600"E
NK0121	LC711218	12			Nagano (NK)	36°49'31.943"N	138°09'12.600"E
NK0122	LC711219	63			Nagano (NK)	36°49'31.943"N	138°09'12.600"E
SK011	LC711273	3	171 & 188, 1172 & 189 , 174 & 191, 173		Shizuoka (SK01)	34°44'14.712"N	138°04'45.155"E

			& 190 ,<br>175 & 192				
SK0119	LC711274	70			Shizuoka (SK01)	34°44'14.712"N	138°04'45.155"E
SK012	LC711272	98			Shizuoka (SK01)	34°44'14.712"N	138°04'45.155"E
SH012	LC711263	71		*L.* sp. NIIGATA1 (n=1; SH0105)	Shizuoka (SH01)	33°26'01.247"N	130°22'10.991"E
SH011	LC711262	71			Shizuoka (SH01)	34°46'25.273"N	137°44'46.751"E
SH0102	LC711261	3			Shizuoka (SH01)	34°46'25.273"N	137°44'46.751"E
O011	LC711236	72		*L.* sp. NIIGATA1 (n=2; O0101, O0107)	Osaka (O1)	34°49'19.038"N	135°31'31.015"E
O012	LC711237	72			Osaka (O1)	34°49'19.038"N	135°31'31.015"E
Wk1_bu36	LC711301	67		*L.* sp. NIIGATA1 (n=6; bu46, bu36, Che2293, Che2294, Che2294, Che2290)	Wakayama (Wk1)	34°09'35.387"N	135°14'00.060"E
STY231	LC711285	4		*L. japonicum* (n=3; Y2314, Y2316, Y2301)	Yamaguchi (Y23)	34°21'06.389"N	132°03'12.958"E
					Tottori (T66)	35°15'20.535"N	134°18'41.410"E

					Shimane (S58)	35°22'02.496"N	133°11'02.430"E
STY233	LC711286	4			Yamaguchi (Y23)	34°21'06.389"N	132°03'12.958"E
					Tottori (T66)	35°15'20.535"N	134°18'41.410"E
					Shimane (S58)	35°22'02.496"N	133°11'02.430"E
Tr-Sh-Ym-Hr (Tottori-Shimane-Yamaguchi-Hiroshima)	LC711296	6		*L. japonicum* (n=3; Che-02081, Che-02082, Che-02083)	Shimane (Shi61)	35°14'42.757"N	132°42'41.024"E
				L. japonicum (n=2;Che-02085, Che-02086)	Shimane (Shi66)	34°52'37.424"N	132°04'44.273"E
				L. japonicum (n=4; Che-02087, Che-02089, Che-02090, Che-02091)	Yamaguchi (Y21)	34°09'44.356"N	132°03'24.612"E
				L. japonicum (n=2;Che-02169, Che-02170)	Hiroshima (Hrs19)	34°33'26.751"N	132°45'55.904"E
				L. japonicum (n=1;Che-02242)	Yamaguchi (Y45)	34°16'57.320"N	131°38'59.413"E

			L. japonicum (n=2; Che-02274, Che-02278)	Shimane (Shi122)	35°10'15.692"N	132°51'07.393"E
			L. japonicum (n=2; Che-02291, Che-02292)	Tottori (Tr80)	35°28'19.983"N	134°08'30.564"E
Ym21-2088	LC712390	6	*L. japonicum* (n=1; Che-02291, Che-2088)	Yamaguchi (Y21)	34°09'44.356"N	132°03'24.612"E
Y23-22-1	LC711303	84	*L. koreanum* (n=2; Che2092, Y2315)	Yamaguchi (Y23)	34°21'06.389"N	132°03'12.958"E
				Yamaguchi (Y21)	34°09'44.356"N	132°03'24.612"E
Shi66-Che2084	LC711271	6	*L. japonicum* (n=18; Che2084)	Shimane (Shi66)	34°52'37.424"N	132°04'44.273"E
Shi5-19_Che2199	LC711270	73	*L.* sp. NIIGATA1 (n=2; Che2220, Che2199)	Shimane (Shi19)	35°19'02.005"N	132°54'46.555"E
				Shimane (Shi05)	35°26'10.222"N	132°52'46.819"E
Shi122_Che2275	LC711266	6	*L. japonicum* (n=1; Che-02275)	Shimane (Shi122)	35°10'15.692"N	132°51'07.393"E
Shi122_Che2276	LC711264	6	*L. japonicum* (n=1; Che-02298)	Shimane (Shi122)	35°10'15.692"N	132°51'07.393"E
Shi124_Che2298	LC711267	6		Shimane (Shi124)	34°45'13.348"N	132°03'11.846"E

Shi124_Che2300	LC711268	109	*L.* sp. CHUGOKU1 (n=1; Che2300)	Shimane (Shi124)	34°45'13.348"N	132°03'11.846"E
Shi124_Che2302	LC711269	83	*L. koreanum* (n=1; Che2302)	Shimane (Shi124)	34°45'13.348"N	132°03'11.846"E
Shi115_Che2306	LC711265	109	*L.* sp. CHUGOKU1 (n=2; Che2306, Che2308)	Shimane (Shi115)	35°05'47.491"N	132°50'17.790"E
S5807	LC711255	4		Shimane (S58)	35°22'02.496"N	133°11'02.430"E
S5809	LC711256	4		Shimane (S58)	35°22'02.496"N	133°11'02.430"E
S5816	LC711257	4	*L. japonicum* (n=1; 5816)	Shimane (S58)	35°22'02.496"N	133°11'02.430"E
Oki29_bu241	LC711245	108		Oki (29)	36°06'07.167"N	133°07'08.198"E
Oki93-Che2140	LC711246	107	*L.* sp. CHUGOKU1 (n=1; Che2140)	Oki (93)	36°16'15.216"N	133°19'18.700"E
Oki94-95-Che2143	LC711247	107	*L.* sp. CHUGOKU1 (n=1; Che2143)	Oki (94)	36°16'46.773"N	133°20'10.283"E
			L. sp. CHUGOKU1 (n=1; Che2144)	Oki (95)	36°16'30.320"N	133°19'59.059"E

Oki95_Che2145	LC711248	107	*L.* sp. CHUGOKU1 (n=1; Che2145)	Oki (95)	36°16'30.320"N	133°19'59.059"E
Oki98_Che2147	LC711249	107	*L.* sp. CHUGOKU1 (n=1; Che2147)	Oki (98)	36°13'10.191"N	133°11'54.473"E
Oki98_Che2148	LC711250	107	*L.* sp. CHUGOKU1 (n=1; Che2148)	Oki (98)	36°13'10.191"N	133°11'54.473"E
Oki99_Che2151	LC711251	108	*L.* sp. CHUGOKU1 (n=1; Che2151)	Oki (99)	36°15'57.053"N	133°16'16.343"E
T6613	LC711293	111	*L. japonicum* (n=1; T6613)	Tottori (T66)	35°15'20.535"N	134°18'41.410"E
Tr77_Che2258	LC711294	68		Tottori (Tr77)	35°21'32.473"N	134°24'51.009"E
Tr80_Che2293	LC711295	68		Tottori (Tr80)	35°28'19.985"N	134°08'30.566"E
Oym15_Che2182	LC711252	6	*L. japonicum* (n=1; Che-02182)	Okayama (Oym15)	34°44'16.195"N	133°24'23.564"E
Oym15_Che2183	LC711253	87	*L. japonicum* (n=1; Che2183)	Okayama (Oym15)	34°44'16.195"N	133°24'23.564"E
Ym25_Che778	LC711304	109	*L.* sp. CHUGOKU1 (n=1; Che2227)	Yamaguchi (25)	34°11'23.850"N	131°45'53.735"E

Y232	LC711302	4		Yamaguchi (Y23)	34°21'06.389"N	132°03'12.958"E
Ym41_Che2227	LC711305	110	*L*. sp. CHUGOKU1 (n=1; Che778)	Yamaguchi (Y41)	34°00'30.675"N	131°49'07.186"E
Ym41_Che2228	LC711306	110	*L*. sp. CHUGOKU1 (n=1; Che2228)	Yamaguchi (Y41)	34°00'30.675"N	131°49'07.186"E
Ym41_Che2229	LC711307	84	*L. koreanum* (n=1; Che2229)	Yamaguchi (Y41)	34°00'30.675"N	131°49'07.186"E
Ym41_Che2230	LC711308	84	*L. koreanum* (n=1; Che2230)	Yamaguchi (Y41)	34°00'30.675"N	131°49'07.186"E
Ym45_Che2244	LC711309	84	*L. koreanum* (n=1; Che2240)	Yamaguchi (Y45)	34°16'57.320"N	131°38'59.413"E
S-T112	LC711284	65		Tokushima (T11)	33°47'57.617"N	134°25'50.688"E
		65	*L. japonicum* (n=1; S5817)	Shimane (S58)	35°22'02.496"N	133°11'02.430"E
T1101	LC711287	65	*L*. sp. NIIGATA1 (n=1; T1101)	Tokushima (T11)	33°47'57.617"N	134°25'50.688"E
T1102	LC711288	65		Tokushima (T11)	33°47'57.617"N	134°25'50.688"E
T111	LC711289	65	*L*. sp. NIIGATA1 (n=3; T1104, T1105, T1110)	Tokushima (T11)	33°47'57.617"N	134°25'50.688"E

T113	LC711292	65		*L*. sp. NIIGATA1 (n=2; T1103, T1107)	Tokushima (T11)	33°47'57.617"N	134°25'50.688"E
T1115	LC711290	102			Tokushima (T11)	33°47'57.617"N	134°25'50.688"E
T1116	LC711291	65			Tokushima (T11)	33°47'57.617"N	134°25'50.688"E
Eh6_Che2309	LC711185	91			Ehime (E6)	34°00'04.577"N	133°36'26.421"E
Eh7_Che2310	LC711186	91			Ehime (E7)	33°52'20.516"N	133°09'46.280"E
E0801	LC711164	101		*L*. sp. EHIME1 (n=1; E0801)	Ehime (E8)	33°53'16.994"N	133°00'40.686"E
E0802	LC711165	91			Ehime (E8)	33°53'16.994"N	133°00'40.686"E
E0803	LC711166	91		*L. koreanum* (n=1; E0803)	Ehime (E8)	33°53'16.994"N	133°00'40.686"E
none			177		Ehime (E8)	33°53'16.994"N	133°00'40.686"E
E0805	LC711167	91			Ehime (E8)	33°53'16.994"N	133°00'40.686"E
E0806	LC711168	91			Ehime (E8)	33°53'16.994"N	133°00'40.686"E
E0807	LC711169	101	194		Ehime (E8)	33°53'16.994"N	133°00'40.686"E
E0810	LC711170	101	179	*L*. sp. EHIME1 (n=1; E0810)	Ehime (E8)	33°53'16.994"N	133°00'40.686"E
E0812	LC711171	101	176	*L*. sp. EHIME1 (n=1; wa04425)	Ehime (E8)	33°53'16.994"N	133°00'40.686"E
E088	LC711163	101	180		Ehime (E8)	33°53'16.994"N	133°00'40.686"E
Eh13_Che2079	LC711176	103			Ehime (E13)	33°35'12.645"N	132°46'30.000"E

Eh14_Che2080	LC711177	103		Ehime (E14)	33°32'15.268"N	132°53'27.232"E
Eh14_Che2317	LC711178	103		Ehime (E14)	33°32'15.268"N	132°53'27.232"E
Eh14_Che2318	LC711179	103		Ehime (E14)	33°32'15.268"N	132°53'27.232"E
Eh14_Che2320	LC711180	103		Ehime (E14)	33°32'15.268"N	132°53'27.232"E
E1509	LC711172	86		Ehime (E15)	33°29'36.996"N	132°32'14.629"E
E151	LC711173	86	*L. koreanum* (n=3; wa04427 (E1512), wa04428 (E1513), E1507)	Ehime (E15)	33°29'36.996"N	132°32'14.629"E
E1510	LC711174	103	*L. japonicum* (n=1; Che2183)	Ehime (E15)	33°29'36.996"N	132°32'14.629"E
E152	LC711175	86	*L. koreanum* (n=2; E1501, E1511)	Ehime (E15)	33°29'36.996"N	132°32'14.629"E
Eh18_Che2314	LC711181	85		Ehime (E18)	33°34'01.264"N	132°27'06.817"E
Eh19_Che2322	LC711183	85		Ehime (E19)	33°28'43.280"N	132°18'01.548"E
Eh19_Che2312	LC711182	103		Ehime (E19)	33°28'43.280"N	132°18'01.548"E
Eh20_Che2313	LC711184	103		Ehime (E20)	33°21'15.486"N	132°38'26.832"E
Ko14_Che2327	LC711202	90		Kouchi (Ko14)	33°12'55.674"N	132°50'57.625"E
Ko14_Che2315	LC711203	90		Kouchi (Ko14)	33°12'55.674"N	132°50'57.625"E

K111	LC711197	92	*L. japonicum* (n=5; K1104, K1105, K1106, K1107, K1108)	Kagawa (K11)	34°07'21.572"N	134°06'08.099"E
K112	LC711198	92		Kagawa (K11)	34°07'21.572"N	134°06'08.099"E
K1123	LC711199	92		Kagawa (K11)	34°07'21.572"N	134°06'08.099"E
Fuk20_Ag229	LC711195	106	*L.* sp. CHUGOKU1 (n=2; Ag229, Ag230)	Ohita (F20)	33°34'51.527"N	130°59'55.248"E
Fuk21_Ag231	LC711196	88	*L. koreanum* (n=2; Ag231, Ag232)	Ohita (F20)	33°34'51.527"N	130°59'55.248"E
Fuk147_Che11	LC711193	104	*L.* sp. CHUGOKU1 (n=1; Che11)	Fukuoka (F147)	33°48'40.535"N	130°35'45.419"E
Fuk147_Che12	LC711194	104	*L.* sp. CHUGOKU1 (n=1; Che12)	Fukuoka (F147)	33°48'40.535"N	130°35'45.419"E
F031	LC711187	82	*L. koreanum* (n=1; F3001)	Fukuoka (F03)	33°47'11.903"N	130°35'17.664"E
F032	LC711188	104	*L* sp. CHUGOKU1 (n=2; Ag227, Ag228)	Fukuoka (F03)	33°47'11.903"N	130°35'17.664"E

				Fukuoka (F161)	33°47'46.211"N	130°36'21.492"E
SG01	LC711258	82	*L. koreanum* (n=1; SG0101)	Saga (SG01)	33°26'01.247"N	130°22'10.991"E
SG02	LC711259	82	*L* sp. CHUGOKU1 (n=2; SG0104, SG0108)	Saga (SG01)	33°26'01.247"N	130°22'10.991"E
SG03	LC711260	105	*L. koreanum* (n=1; SG0114)	Saga (SG01)	33°26'01.247"N	130°22'10.991"E
Kg3_bu51	LC711201	106	*L.* sp. CHUGOKU1 (n=1; bu51)	Kagoshima (Kg3)	31°18'43.379"N	130°31'39.791"E
FI0104	LC711189	96		Fukue (FI01)	32°40'28.676"N	128°42'31.031"E
FI011	LC711190	96	*L.* sp. FUKUE1 (n=2; FI0109, FI0112)	Fukue (FI01)	32°40'28.676"N	128°42'31.031"E
FI012	LC711191	96	*L.* sp. FUKUE1 (n=3; FI0101, FI0102, FI0111)	Fukue (FI01)	32°40'28.676"N	128°42'31.031"E
FI013	LC711192	96	*L.* sp. FUKUE1 (n=2; FI0106, FI0110)	Fukue (FI01)	32°40'28.676"N	128°42'31.031"E
TS031	LC711299	94	*L. koreanum* (n=1; TS0302)	Tsushima (TS03)	34°09'06.487"N	129°12'36.710"E

TS0307	LC711297	94		Tsushima (TS03)	34°09'06.487"N	129°12'36.710"E
TS0309	LC711298	94		Tsushima (TS03)	34°09'06.487"N	129°12'36.710"E
Tsm28_Che21	LC711300	94	*L. koreanum* (n=2; Che21, Che22)	Tsushima (TS28)	34°19'20.907"N	129°21'23.036"E
OK0104	LC711238	95	*L. koreanum* (n=1; OK0104)	Okinawa (OK11)	26°43'52.680"N	128°12'34.452"E
OK0110	LC711239	95		Okinawa (OK11)	26°43'52.680"N	128°12'34.452"E
OK0112	LC711240	95		Okinawa (OK11)	26°43'52.680"N	128°12'34.452"E
OK02	LC711241	95	*L. koreanum* (n=1; OK0103)	Okinawa (OK11)	26°43'52.680"N	128°12'34.452"E
OK03	LC711242	95	*L. koreanum* (n=1; OK0102)	Okinawa (OK11)	26°43'52.680"N	128°12'34.452"E
OK04	LC711243	95	*L. koreanum* (n=1; OK0101)	Okinawa (OK11)	26°43'52.680"N	128°12'34.452"E
OK05	LC711244	95		Okinawa (OK11)	26°43'52.680"N	128°12'34.452"E
K113 (*Ligidium* [*Ligidium*] sp.)	LC711200	Outgroups	*Ligidium* (*Ligidium*) sp.	Kagawa (K11)	34°07'21.572"N	134°06'08.099"E
Ligia sp. Niigata2	LC711142	Outgroups		Niigata coastline		
Ligia sp. Niigata3	LC711143	Outgroups		Niigata coastline		

(B) Data list from GeneBank.

Sequence ID	Accession number	Location	Mt DNA-OTU
*Ligidium ryukyuense**	AB626261.1	Amami-Oshima, Kagoshima, Japan	*Ligidium ryukyuense**
Ligidium sp. SHIZU2	LC496507.1	Hamamatsu, Shizuoka, Japan	OTU3
Ligidium sp. SHIZU1	LC496506.1	Hamamatsu, Shizuoka, Japan	OTU98
Ligidium beieri	DQ182830.1	Greece	*Ligidium* sp. (Greece & Austria)
	DQ182857.1	Greece	*Ligidium* sp. (Greece & Austria)
Ligidium werneri	DQ182830.1	Greece	*Ligidium* sp. (Greece & Austria)
Ligidium cycladicum	DQ182833.1	Greece	*Ligidium* sp. (Greece & Austria)
Ligidium ghigii	DQ182824.1	Greece	*Ligidium* sp. (Greece & Austria)
Ligidium germanicum	DQ182797.1	Greece	*Ligidium* sp. (Greece & Austria)
Ligidium hypnorum	AY051319.1	Nr. Fugen, Austria	*Ligidium* sp. (Greece & Austria)
Oniscidea sp. JXAUM-L14104	KT447508.1	China	*Ligidium* sp. (China)
Oniscidea sp. JXAUM-L14103	KT447508.1	China	*Ligidium* sp. (China)
Oniscidea sp. JXAUM-L14102	KT447507.1	China	*Ligidium* sp. (China)
Oniscidea sp. JXAUM-L14101	KT447506.1	China	*Ligidium* sp. (China)
Oniscidea sp. JXAUM-L14100	KT447505.1	China	*Ligidium* sp. (China)
Oniscidea sp. JXAUM-L14098	KT447504.1	China	*Ligidium* sp. (China)
Oniscidea sp. JXAUM-L14111	KT447503.1	China	*Ligidium* sp. (China)
Oniscidea sp. JXAUM-L14110	KT447502.1	China	*Ligidium* sp. (China)
Oniscidea sp. JXAUM-L14085	KT447501.1	China	*Ligidium* sp. (China)
Oniscidea sp. JXAUM-L14083	KT447500.1	China	*Ligidium* sp. (China)

Oniscidea sp. JXAUM-L14082	KT447499.1	China	*Ligidium* sp. (China)
Oniscidea sp. JXAUM-L14115	KT447498.1	China	*Ligidium* sp. (China)
Oniscidea sp. JXAUM-L14114	KT447497.1	China	*Ligidium* sp. (China)
Ligia perkinsi	AY051337.1	Hawaiian Islands, USA	Outgroups
Ligia italica	DQ182861.1		Outgroups
Gose-shi_Nara1567_a1	LC565854	Gose-shi, Nara1567	OTU5
Fukaura-cho_a1	LC565849	Fukaura-cho, Akita	OTU8
Fukaura-cho_Akita_b2	LC565850	Fukaura-cho, Akita	
Yuzawa-shi_a1	LC566056	Yuzawa-shi, Akita	OTU9
Saitamashi1	LC565995	Saitama-shi, Saitama	OTU11
	LC565996	Saitama-shi, Saitama	
Miharu-cho_2	LC565933	Miharu-cho, Fukushima	OTU13
Gunma1	LC566050	Gunma	OTU14
	LC566051	Gunma	
	LC566052	Gunma	
Hitachi-shi2	LC565863	Hitachi-shi, Ibaraki	OTU15
Itabashi-ku_b2	LC565887	Itabashi-ku, Tokyo	OTU16
Saitama1	LC565912	Saitama	
	LC565913	Saitama	
Mt. Kanozan_h8	LC565946	Mt. Kanozan	
Owasi_i9	LC565990	Kimitsu-shi, Chiba	
Amigasamori-Forest_a1	LC565844	Hirosaki-shi, Aomori	OTU19
Dakigaeri-Valley_Akita_a1	LC565848	Dakigaeri-Valley, Akita	OTU22

Yuzawa-shi_b2	LC566057	Yuzawa-shi, Akita	OTU20
Owasi_c3	LC565984	Kimitsu-shi, Chiba	OTU23
Owasi_e5	LC565987	Kimitsu-shi, Chiba	
Tozaki_3	LC566043	Kimitsu-shi, Chiba	
Tozaki_c5	LC566046	Kimitsu-shi, Chiba	
Owasi_Kanozan1	LC565985	Kimitsu-shi, Chiba	
	LC565941	Kimitsu-shi, Chiba	
	LC565944	Kimitsu-shi, Chiba	
	LC565945	Kimitsu-shi, Chiba	
Chiba_Owasi1	LC565982	Kimitsu-shi, Chiba	
	LC565983	Kimitsu-shi, Chiba	
	LC565988	Kimitsu-shi, Chiba	
	LC565989	Kimitsu-shi, Chiba	
	LC565991	Kimitsu-shi, Chiba	
Higashinosawa_4	LC565857	Kamogawa-shi, Chiba	OTU24
Naraihara_1	LC565964	Kamogawa-shi, Chiba	
Okawazura2	LC565974	Kamogawa-shi, Chiba	
	LC565976	Kamogawa-shi, Chiba	
Minamiboso-shi_a1	LC565934	Minamiboso-shi, Chiba	
Okawazura_d6	LC565978	Kamogawa-shi, Chiba	
Toyoka1	LC566039	Futtsu-shi, Chiba	OTU25
	LC566041	Futtsu-shi, Chiba	
Toyoka_b2	LC566040	Futtsu-shi, Chiba	

Kiwadahata_i9	LC565925	Kimitsu-shi, Chiba	
Chiba-Hiroka1	LC565859	Kimitsu-shi, Chiba	OTU26
	LC565860	Kimitsu-shi, Chiba	
	LC565861	Kimitsu-shi, Chiba	
	LC565862	Kimitsu-shi, Chiba	
Chiba-Tozaki c5	LC566046	Kimitsu-shi, Chiba	OTU27
Chiba-Mt. Nokogiriyama1	LC565949	Futtsu-shi, Chiba	OTU28
	LC565950	Futtsu-shi, Chiba	
	LC565951	Futtsu-shi, Chiba	
Takataki_i9	LC566033	Ichihara-shi, Chiba	OTU29
Kawayatu_k11	LC565909	Kimitsu-shi, Chiba	
Kawayatu_f6	LC565906	Kimitsu-shi, Chiba	
Kawayatu_h8	LC565908	Kimitsu-shi, Chiba	
Kiwadahata_e5	LC565921	Kimitsu-shi, Chiba	
Kiwadahata_h8	LC565924	Kimitsu-shi, Chiba	
Kiwadahata1	LC565917	Kimitsu-shi, Chiba	
	LC565918	Kimitsu-shi, Chiba	
	LC565923	Kimitsu-shi, Chiba	
	LC565926	Kimitsu-shi, Chiba	
Kiwadahata2	LC565927	Kimitsu-shi, Chiba	
	LC565919	Kimitsu-shi, Chiba	
	LC565920	Kimitsu-shi, Chiba	
	LC565922	Kimitsu-shi, Chiba	

Kiwadahata_g7	LC565923	Kimitsu-shi, Chiba	
Ishigami_c3	LC565878	Ichihara-shi, Chiba	
Ishigami_k11	LC565886	Ichihara-shi, Chiba	
Ishigami_i9	LC565884	Ichihara-shi, Chiba	
Katori1	LC565904	Katori-shi, Chiba	OTU30
Katori-shi_e5	LC565905	Katori-shi, Chiba	
Katori-shi_a1	LC565903	Katori-shi, Chiba	
Sanmu-shi1	LC565999	Sanmu-shi, Chiba	OTU31
	LC566000	Sanmu-shi, Chiba	
	LC566003	Sanmu-shi, Chiba	
	LC566004	Sanmu-shi, Chiba	
Sanmu-shi2	LC566001	Sanmu-shi, Chiba	
	LC566002	Sanmu-shi, Chiba	
Chiba-Sosa c3	LC566020	Sosa-shi, Chiba	OTU32
Kawayatu_g7	LC565907	Kimitsu-shi, Chiba	OTU33
Kawayatu_h8	LC565908	Kimitsu-shi, Chiba	
Mutsuzawa-cho_b2	LC565960	Mutsuzawa-cho, Chiba	
Ii_Tochigi_2	LC565865	Motegi-cho, Tochigi	OTU34
	LC565866	Motegi-cho, Tochigi	
Ii_Tochigi_1	LC565864	Motegi-cho, Tochigi	
	LC566011	Shimotsuma, Ibaraki	
	LC566013	Shimotsuma, Ibaraki	
Gunma-Shimohino	LC566006	Fujioka-shi, Gunma	OTU35

	LC566007	Fujioka-shi, Gunma	
Tsukuba1	LC565952	Tsukuba-shi, Ibaraki	OTU36
	LC565953	Tsukuba-shi, Ibaraki	
	LC565954	Tsukuba-shi, Ibaraki	
	LC565957	Tsukuba-shi, Ibaraki	
	LC565958	Tsukuba-shi, Ibaraki	
Tsukuba_c4	LC565955	Tsukuba-shi, Ibaraki	
Kashimajingu-Station1	LC565897	Kashima-shi, Ibaraki	OTU37
Kashima1	LC565898	Kashima-shi, Ibaraki	
	LC565901	Kashima-shi, Ibaraki	
Kashima2	LC565899	Kashima-shi, Ibaraki	
	LC565902	Kashima-shi, Ibaraki	
Kashimajingu-Station4	LC565900	Kashima-shi, Ibaraki	
Shimotsuma-shi4	LC566014	Shimotsuma-shi, Ibaraki	OTU38
Shimotsuma-shi2	LC566012	Shimotsuma-shi, Ibaraki	
Kamimiyakawauchi_a1	LC565891	Hitatiota-shi, Ibaraki	OTU39
Kamimiyakawauchi_b2	LC565892	Hitatiota-shi, Ibaraki	
Tabina_3	LC566021	Kimitsu-shi, Chiba	OTU41
Tabina_b4	LC566022	Kimitsu-shi, Chiba	
Okawazura1	LC565973	Kamogawa-shi, Chiba	
	LC565975	Kamogawa-shi, Chiba	
	LC565977	Kamogawa-shi, Chiba	
	LC565979	Kamogawa-shi, Chiba	

	LC565980	Kamogawa-shi, Chiba	
	LC565981	Kamogawa-shi, Chiba	
Tozaki_f8	LC566049	Kimitsu-shi, Chiba	OTU42
Tozaki1	LC566042	Kimitsu-shi, Chiba	
	LC566044	Kimitsu-shi, Chiba	
	LC566045	Kimitsu-shi, Chiba	
	LC566047	Kimitsu-shi, Chiba	
	LC566048	Kimitsu-shi, Chiba	
Mt. Kanozan_a3	LC565939	Kimitsu-shi, Chiba	OTU43
Mt. Kanozan_d4	LC565942	Kimitsu-shi, Chiba	
Mt. Kanozan_b1	LC565940	Kimitsu-shi, Chiba	
Mt. Kanozan1	LC565943	Kimitsu-shi, Chiba	
	LC565947	Kimitsu-shi, Chiba	
Chiba-Kawayatu l12	LC565910	Kimitsu-shi, Chiba	OTU44
Midori-ku_c3	LC565932	Chiba-shi, Chiba	OTU45
Ishigami_a1	LC565876	Ichihara-shi, Chiba	OTU46
	LC565877	Ichihara-shi, Chiba	
	LC565880	Ichihara-shi, Chiba	
	LC565881	Ichihara-shi, Chiba	
	LC565882	Ichihara-shi, Chiba	
	LC565885	Ichihara-shi, Chiba	
Ishigami_d4	LC565879	Ichihara-shi, Chiba	
Takataki3	LC566025	Ichihara-shi, Chiba	OTU47

	LC566036	Ichihara-shi, Chiba	
Takataki1	LC566026	Ichihara-shi, Chiba	OTU48
	LC566028	Ichihara-shi, Chiba	
	LC566030	Ichihara-shi, Chiba	
	LC566031	Ichihara-shi, Chiba	
	LC566032	Ichihara-shi, Chiba	
	LC566035	Ichihara-shi, Chiba	
Takataki2	LC566027	Ichihara-shi, Chiba	
	LC566029	Ichihara-shi, Chiba	
Takataki_j10	LC566034	Ichihara-shi, Chiba	
Sosa-shi_a1	LC566019	Sosa-shi, Chiba	OTU49
Tateyama1	LC566037	Tateyama-shi, Chiba	OTU50
	LC566038	Tateyama-shi, Chiba	
Kanagawa_Ikuta1	LC565867	Kawasaki-shi, Kanagawa	OTU51
	LC565868	Kawasaki-shi, Kanagawa	
Ikuta-Ryokuchi-Park_d4	LC565870	Kawasaki-shi, Kanagawa	
Kanagawa_Ikuta3	LC565871	Kawasaki-shi, Kanagawa	
	LC5658734	Kawasaki-shi, Kanagawa	
Ikuta-Ryokuchi-Park_g7	LC565873	Kawasaki-shi, Kanagawa	
Ikuta2	LC565869	Kawasaki-shi, Kanagawa	
	LC565872	Kawasaki-shi, Kanagawa	
Yokohama1	LC565969	Yokohama-shi, Kanagawa	OTU52
	LC565970	Yokohama-shi, Kanagawa	

Niharu_Yokohama_b2	LC565971	Yokohama-shi, Kanagawa	
Shinbayashi-park_b2	LC566015	Fujisawa-shi, Kanagawa	OTU53
Shinbayashi-park_f6	LC566016	Fujisawa-shi, Kanagawa	
Shinbayashi-park_g7	LC566017	Fujisawa-shi, Kanagawa	
Shinbayashi-park_h8	LC566018	Fujisawa-shi, Kanagawa	
Chibaraki1	LC565997	Chiba/Ibaraki	OTU54
	LC565914	Chiba/Ibaraki	
	LC565915	Chiba/Ibaraki	
	LC565916	Chiba/Ibaraki	
	LC565967	Chiba/Ibaraki	
	LC565968	Chiba/Ibaraki	
	LC565998	Chiba/Ibaraki	
Narita-shi_a1	LC565965	Narita-shi, Chiba	
Narita-shi_b2	LC565966	Narita-shi, Chiba	
Funabashi1	LC565851	Funabashi-shi, Chiba	
	LC565853	Funabashi-shi, Chiba	
	LC565852	Funabashi-shi, Chiba	
Tokyo-Akiruno b1	LC565843	Akiruno-shi, Tokyo	OTU55
Hamura-shi_b1	LC565856	Hamura-shi, Tokyo	OTU56
Takao-cho_2	LC566023	Hachioji-shi, Tokyo	OTU57
Takao-cho_a1	LC566024	Hachioji-shi, Tokyo	
Kanra-cho_Gunma_1	LC565894	Kanra-cho, Gunma	OTU58
Kanra-cho_Gunma_3	LC565896	Kanra-cho, Gunma	

Kawazu-cho_1	LC565911	Kawazu-cho, Shizuoka	OTU59
Yamanashi1	LC566053	Yamanashi	
	LC566054	Yamanashi	
	LC566055	Yamanashi	
Izukuni1	LC565888	Izunokuni-shi, Shizuoka	
	LC565889	Izunokuni-shi, Shizuoka	
	LC565890	Izunokuni-shi, Shizuoka	
Hirayu_Gifu_a1	LC565858	Takayama-shi, Gifu	OTU61
Gunma1	LC565962	Gunma	OTU62
	LC566008	Gunma	
	LC565961	Gunma	
	LC566010	Gunma	
Nakanojo-machi_3	LC565963	Nakanojo-machi, Gunma	
Shimonita-cho_2	LC566009	Shimonita-cho, Gunma	
Miyazawa-cho_1	LC565935	Takasaki-shi, Gunma	
Miyazawa1	LC565937	Takasaki-shi, Gunma	
	LC565938	Takasaki-shi, Gunma	
Miyazawa-cho_2	LC565936	Takasaki-shi, Gunma	
Gose-shi_b2	LC565855	Gose-shi, Nara	OTU66
Bizen-shi_Okayama_1	LC565846	Bizen-shi, Okayama	OTU69
Notojima-Island_Ishikawa_a1	LC565972	Notojima-Island, Ishikawa	OTU76
Anamizu-cho_Ishikawa_a1	LC565845	Anamizu-cho, Ishikawa	OTU78
Chichibu-shi_Saitama_3	LC565847	Chichibu-shi, Saitama	OTU81

Isato-cho_Mie_a1	LC565875	Isato-cho_Mie_a1	OTU93
Kamioka-cho-do_Gifu_a1	LC565893	Hida-shi, Gifu	OTU97
Sahama-cho_Hamamatsu_1	LC565994	Hamamatsu-shi, Shizuoka	OTU98
Kanra-cho_Gunma_2	LC565895	Kanra-cho, Gunma	OTU99
Toyoka_Chiba_b2	LC566040	Futtsu-shi, Chiba	OTU100
Sagiura_Shimane_1	LC565992	Izumo-shi, Shimane	OTU109

*****This species was misidentified. Reexamination of the specimen revealed that it was close to *Ligidium koreanum*.

Supplementary Reference

1. Kozlov, A. M., Darriba, D., Flouri, T., Morel, B., & Stamatakis, A. RAxML-NG: a fast, scalable and user-friendly tool for maximum likelihood phylogenetic inference. *Bioinformatics* **35,** 4453-4455 (2019).
2. Darriba, D., Posada, D., Kozlov, A. M., Stamatakis, A., Morel, B., & Flouri, T. (2020). ModelTest-NG: a new and scalable tool for the selection of DNA and protein evolutionary models. *Mol. Bio. Evol.* **37,** 291-294.
